# A New Notosuchian from the Late Cretaceous of Brazil and the Phylogeny of Advanced Notosuchians

**DOI:** 10.1371/journal.pone.0093105

**Published:** 2014-04-02

**Authors:** Diego Pol, Paulo M. Nascimento, Alberto B. Carvalho, Claudio Riccomini, Ricardo A. Pires-Domingues, Hussam Zaher

**Affiliations:** 1 CONICET, Museo Paleontológico Egidio Feruglio, Trelew, Chubut, Argentina; 2 Museu de Zoologia da Universidade de São Paulo, São Paulo, Brazil; 3 Programa de Pós-Graduação em Zoologia, Instituto de Biociências, Universidade de São Paulo, São Paulo, Brazil; 4 Instituto de Geociências, Universidade de São Paulo, São Paulo, Brazil; College of the Holy Cross, United States of America

## Abstract

A new notosuchian crocodyliform from the Late Cretaceous Bauru Group found in the southeastern State of São Paulo (Brazil) is described here. The new taxon, *Caipirasuchus stenognathus*, is referred as a new species of the recently erected genus *Caipirasuchus* within the clade Sphagesauridae based on a phylogenetic analysis of basal mesoeucrocodylians. *Caipirasuchus stenognathus* is represented by an almost complete skull and lower jaw that has autapomorphic characters that distinguish it from other species of Sphagesauridae. These autapomorphies include: maxilla forming part of the orbital margin (absence of lacrimal-jugal contact), nasal with smooth depressions on the posterior region close to the contact with the maxilla and lacrimal, postorbital with posterior palpebral facet that extends posteriorly underneath the ear-flap groove, and a distinct anterior process of the medial flange of the retroarticular process. Additionally, the new taxon lacks autapomorphic features described in other sphagesaurids. The phylogenetic analysis results in a monophyletic genus *Caipirasuchus*, that is the sister group of a clade fomed by *Sphagesaurus huenei*, *Caryonosuchus pricei*, and *Armadillosuchus arrudai*. Sphagesaurids also include a basal clade formed by *Adamantinasuchus navae* and *Yacarerani boliviensis*. Other notosuchian taxa, such as *Mariliasuchus amarali*, *Labidiosuchus amicum*, *Notosuchus terrestris*, and *Morrinhosuchus luziae* are successive sister taxa of Sphagesauridae, forming a clade of advanced notosuchians that are restricted to the Late Cretaceous of South America. These results contrast with most previous phylogenetic hypotheses of the group that depicted some members of Sphagesauridae as more closely related to baurusuchids, or found Asian (e.g., *Chimaerasuchus*) or African (*Malawisuchus*, *Pakasuchus*) forms nested within advanced notosuchians that are, according to our analysis, endemic of the Late Cretaceous of South America.

## Introduction

Notosuchian crocodyliforms were extremely diverse in the Cretaceous of Gondwana and are particularly abundant in some Late Cretaceous deposits of South America [1]. Members of this group have been interpreted as terrestrial organisms, given the absence of widely recognized adaptations to the aquatic environment present in other crocodyliforms [2], [3]. Notosuchians are usually characterized by the presence of a heterodont dentition and a broad range of morphologies has been reported for their posterior teeth (including multicusped crowns). Several authors [3]–[5] suggested the presence of a fore-aft pattern of jaw motion that contrasts with those of other crocodyliforms (including living crocodylians).

Sphagesauridae is a group nested within Notosuchia that was, until recently, only known from poorly preserved remains from the Late Cretaceous Bauru Group [5]. A set of unique apomorphic features characterizes sphagesaurid dentition, including the presence of posterior teeth that are obliquely oriented (also present in *Notosuchus*) that bear strongly developed and well spaced apicobasal ridges, a wrinkled enamel surface, and a distal (maxillary) or mesial (dentary) keel with large tuberous denticles [6], [7].

The first sphagesaurid remains described were two isolated teeth (type material of *Sphagesaurus huenei*; [8]) that had all these characters and led [9] to erect a monotypic family for this taxon. Over 50 years later, an almost complete skull (but with only a partially preserved dentition) was described and referred to this taxon [5]. Subsequently, a second taxon was described and referred to this genus (*Sphagesaurus montealtensis*) based on a relatively complete skull and mandible but that lacked most of the enamel surface of the teeth. This new taxon shared most of the dental features described above, as well as other derived craniomandibular characters [10], with the skull referred to *S. huenei* [5].

More recently, the generic diversity of Sphagesauridae increased with the description of new taxa. A new taxon was described [7] based on a well-preserved skull and partially preserved postcranium, and for which the genus *Armadillosuchus* was erected. Despite the completeness and overall excellent preservation of its holotype, the palate and toothrow of *Armadillosuchus arrudai* was incompletely preserved but the available tooth remains shared the above-mentioned dental features of *S. huenei*. A third genus of Sphagesauridae, *Caipirasuchus*, was erected [11] for the new species *C. paulistanus*, based on an almost complete skull and mandible that include a complete toothrow (although several details of its dentition are not well preserved). The holotype of *Caipirasuchus paulistanus* is remarkably similar to that of *Sphagesaurus montealtensis* and, although the two taxa can be distinguished based on a few characters (see below), their similarities have led Iori *et al.* [12] to consider them cogeneric (erecting the new combination *Caipirasuchus montealtensis*, which is adopted here). A fourth genus, *Caryonosuchus*, was also recently erected [13] based on a rostrum and symphyseal mandibular region that differs from other sphagesaurids by the presence of notorious protuberances and other ornamentations on the dorsal surface of the rostrum. The dentition of *Caryonosuchus* is incompletely preserved, but several teeth have remarkable similarities with that of *S. huenei* and *Armadillosuchus*, corroborating the referral of this taxon to Sphagesauridae.

Furthermore, *Adamantinasuchus navae* [14] and *Yacarerani boliviensis* [15] are two recently described notosuchians that share with sphagesaurids some (but not all) apomorphic dental features noted above. The similarities between the dentition of *Adamantinasuchus navae* and *Sphagesaurus huenei* have been recognized and the former taxon was included in Sphagesauridae [6], a position criticized by other authors [16]. *Yacarerani* is characterized by a highly apomorphic dentition but also shares the similarities noted [6] for *Adamantinasuchus* and sphagesaurids. Therefore, the more inclusive list of taxa that could be referred to Sphagesauridae currently includes seven different taxa recognized in six different genera.

Despite the set of apomorphic features in the dentition shared by the above-mentioned taxa, monophyly of Sphagesauridae have been repeatedly questioned in recent phylogenetic analyses, probably due to limited character sampling. The first phylogenetic studies of the affinities of *Sphagesaurus huenei* [5], [17] depicted the then only known sphagesaurid as more closely related to baurusuchids than to other notosuchians (e.g., *Mariliasuchus*, *Notosuchus*). The second described sphagesaurid (*Caipirasuchus montealtensis*) was depicted as the sister group of *Sphagesaurus huenei* [10]. However, subsequent phylogenetic studies that included newly discovered sphagesaurids (e.g., *Armadillosuchus*, *Adamantinasuchus*, *Yacarerani*, *Caipirasuchus paulistanus*) rejected the monophyly of a clade clustering all the taxa referred to Sphagesauridae [11], [12], [15], [18], [19]. In these studies, some of these taxa (e.g., *Adamantinasuchus*, *Yacarerani*, *Armadillosuchus*) were depicted as more closely related to baurusuchids [18] or to other notosuchians (e.g., *Mariliasuchus*; [11], [12], [15], [19]) than to *Sphagesaurus*. Furthermore, in these studies a couple of non-South American taxa were depicted as the sister group of different species referred to Sphagesauridae, such as the sister group relationship of *Pakasuchus* and *Adamantinasuchus* [19] or *Chimaerasuchus* and *Sphagesaurus* [5], [11], [15], [17]–[18]. The only exceptions to the inclusion of non-South American taxa as closely allied to some sphagesaurids were the phylogenetic analyses of Andrade and collaborators [10], [20], [21] and Riff and Kellner [20], that depicted at least some of the sphagesaurid species known by then in a monophyletic group, as the sister group of either baurusuchids [21] or a clade formed by *Notosuchus* and *Mariliasuchus* [10], [20].

The debated phylogenetic relationships of sphagesaurids are an important part of the topological differences obtained for the large clade Notosuchia in recent phylogenetic studies. Most recent authors agree on the monophyly of a large clade composed by taxa traditionally classified as notosuchians (e.g., *Libycosuchus*, *Simosuchus*, *Candidodon*, *Malawisuchus*, *Notosuchus*, *Mariliasuchus*) and baurusuchids [5], [10], [17], [20]–[24]. However, the notosuchian affinities of some taxa have been debated during the last decade and different studies have either included or excluded Sebecidae and the speciose genus *Araripesuchus* from the clade that clusters all other notosuchians [5], [21]–[23], [25]. Moreover, recent studies [18], [20], [26] retrieved peirosaurids as closer to notosuchians than to neosuchians, being the sister group of Uruguaysuchidae (i.e., *Araripesuchus*+*Uruguaysuchus*) [26].

Here, we describe a new sphagesaurid crocodyliform from the Late Cretaceous Adamantina Formation (Bauru Group). The new taxon is represented by an almost complete skull and lower jaw with a remarkably well-preserved and complete dentition, increasing our knowledge on the cranial anatomy and dentition of sphagesaurid crocodyliforms. The relationships of this taxon are evaluated through a phylogenetic analysis based on an expansion of previously published datasets, improving both the taxon and character sampling of the craniomandibular anatomy of notosuchian crocodyliforms.

## Materials and Methods

Materials of fossil crocodyliforms used for comparisons were listed in Table 1, which detailed the most relevant taxa used for comparisons and the collection number of the most informative specimen material. Unless noted otherwise, comparisons for the taxa mentioned in the description were based on these specimens. Most genera are currently monotypic and therefore only the genus name was used for the species listed in Table 1. In order to avoid any confusion, only the genus name for the species of *Araripesuchus*, *Baurusuchus*, *Caipirasuchus*, and *Sphagesaurus* were abbreviated in the text. The terminology used for crocodyliform clades used in this contribution follows the recognition of the following groups as in recent phylogenetic studies. For major clades, such as Crocodyliformes, Mesoeucrocodylia and Neosuchia we follow the usage of Clark [27]. Notosuchia is a major clade of terrestrial crocodyliforms but its taxonomic content has been in a state of flux in recent years (see above). Here we follow the broadened taxonomic content of Notosuchia retrieved in recent studies [18], [20], [26] and in our extended phylogenetic analysis that includes both *Araripesuchus* and peirosaurids as basal members of this clade. The usage of this term is also congruent with the phylogenetic definitions given by Sereno *et al.* [28]. Within notosuchians several clades are recognized, including Ziphosuchia [22], Baurusuchidae [21], [29], [30], Sebecidae [22], Mahajangasuchidae [24], Uruguaysuchidae [31], and Peirosauridae. The latter included forms recorded in the Late Cretaceous of South America (*Lomasuchus*, *Gasparinisuchus*, *Montealtosuchus*, *Uberabasuchus*) and *Hamadasuchus*, but not *Stolokrosuchus* and the clade formed by *Mahajangasuchus* and *Kaprosuchus* (for which Sereno & Larsson [24] recently coined the name Mahajangasuchidae). The term “advanced notosuchians” is used here for a group of derived forms retrieved in our phylogenetic analysis that is deeply nested within Notosuchia and includes *Notosuchus*, *Morrinhosuchus*, *Mariliasuchus*, *Labidiosuchus*, and Sphagesauridae (and possibly the fragmentary *Coringasuchus*). Based on our phylogenetic results Sphageauridae includes the genera *Caipirasuchus*, *Sphagesaurus*, *Armadillosuchus*, *Adamantinasuchus*, and *Yacarerani*, and our usage is consistent with the definition of Sphagesauridae given by Marinho & Carvalho [6]. We avoided the use of groups of disputed monophyly, such as Sebecosuchia [22] and Sebecia [23] given the unstable behavior of Sebecidae in recent phylogenetic analyses.

**Table 1:** List of taxa and specimens used for comparative purposes in the description.

Taxon	Specimen	Reference^1^
*Adamantinasuchus navae*	UFRJ 107R	[14]
*Anatosuchus minor*	MNN GAD603, GAD17	[22], [114]
*Araripesuchus buitreraensis*	MPCA-PV 235	[58]
*Araripesuchus gomesii*	AMNH 24450	[115]
*Araripesuchus patagonicus*	MUC-PV 269, 267	[20]
*Araripesuchus wegeneri*	MNN GAD19	[22], [116]
*Armadillosuchus arrudai*	UFRJ 303R	[7]
*Baurusuchus pachecoi*	DGM 299-R	[117]
*Baurusuchus salgadoensis*	MPMA 62-0001-02	[118]
*Bretesuchus bonapartei*	PVL 4375	[130]
*Caipirasuchus montealtensis*	MPMA 15-001/90, 68-0003/12	[10], [12]
*Caipirasuchus paulistanus*	MPMA 67-0001/00	[11], [12]
*Campinasuchus dinizi*	CPP 1235	[106]
*Candidodon itapecurense*	UFRJ 114R	[119]
*Caryonosuchus pricei*	DGM 1411-R	[13]
*Comahuesuchus brachybuccalis*	MOZ 6131P MUC-PV 202	[55], [78]
*Fruitachampsa callinsoni*	LACM 120455a	[120]
*Gasparinisuchus peirosauroides*	MOZ 1750P	[121], [122]
*Hamadasuchus reboulli*	ROM 52620	[21]
*Hsisosuchus chowi*	ZDM 0146	[123]
*Kaprosuchus saharicus*	MNN IGU 12	[22]
*Labidiosuchus amicum*	DGM 1480-R	[74]
*Libycosuchus brevirostres*	BSP 1912.VIII.574	[124]
*Lomasuchus palpebrosus*	MOZ 4084P	[121]
*Mahajangasuchus insignis*	UA 8654	[23], [56]
*Malawisuchus mwakasyungutiensis*	MAL 45, MAL 49	[112]
*Mariliasuchus amarali*	MZSP-PV 50, 51	[17], [64], [86]
*Montealtosuchus arrudacamposi*	MPMA 16-0007-04	[105]
*Morrinhosuchus luziae*	MPMA 07-0009/01	[77]
*Notosuchus terrestris*	MACN-RN 1037	[61], [86], [88], [91]
*Pakasuchus kapilimai*	RRBP 08631	[19]
*Pissarrachampsa sera*	LPRP/USP 0019	[30]
*Protosuchus richardsoni*	AMNH 3024, MCZ 6727, UCMP 130860, UCMP 131827	[125]
*Simosuchus clarki*	UA 8679	[79], [126]
*Sphagesaurus huenei*	RCL-100	[5], [8]
*Stolokrosuchus lapparenti*	MNN GDF 600	[127]
*Stratiotosuchus maxhechti*	DGM 1477-R, URC R73	[28], [108]
*Uberabasuchus terrificus*	CPP 630	[128]
*Uruguaysuchus aznarezi*	FC-DPV 2320	[31], [129]
*Yacarerani boliviensis*	MNK-PAL 5063	[15]
*Zosuchus davidsoni*	IGM 100/1304-1308	[54]

1 In addition to the number of the specimens studied for this contribution, the reader is also referred to the original reference of each taxon as well as additional detailed descriptions.

### Ethics statement

The material described as a new species in the present study was collected under the regulation of the Departamento Nacional de Produção Mineral (DNPM, Decreto-Lei 4146), for which no specific permits were required. Among authors, only HZ and ABC were involved in the collection of this specimen in the field. The specimen was deposited at the Museu de Zoologia da Universidade de São Paulo and received an appropriate collection number. We thank Mr. Aparecido Barbosa, landowner of the farm where the specimen was found, for allowing access to the site. The authors received permissions for the comparative material listed in Table 1. Geological information and repository numbers are given under species accounts. Institutional abbreviations are as follow: AMNH: American Museum of Natural History, New York, USA: BSP: Bayerische Staatssammlung für Paläontologie und Geologie, Münich, Germany; CPP: Centro de Pesquisas Paleontológicas L. I. Price, Peirópolis, Brazil; DGM: Departamento de Produção Mineral, Rio de Janeiro, Brazil; FC-DPV: Facultad de Ciencias, Colección de Vertebrados Fósiles, Montevideo, Uruguay; IGM: Mongolian Institute of Geology, Ulaan Bataar, Mongolia; IVPP: Institute of Vertebrate Paleontology and Paleoanthropology, Beijing, People's Republic of China; LACM: Los Angeles County Museum, Los Angeles, USA; LPRP: Laboratório de Paleontologia de Riberão Preto, Universidade de São Paulo, Riberão Preto, Brazil; MACN: Museo Argentino de Ciencias Naturales, Buenos Aires, Argentina; MAL: Malawi Department of Antiquities, Malawi; MNK-PAL: Museo Noel Kempff Mercado, Santa Cruz de la Sierra, Bolivia; MOZ: Museo Profesor J. Olsacher, Zapala, Argentina; MPCA: Museo Paleontológico Carlos Ameghino, Cipoletti, Río Negro; MPMA: Museu de Paleontologia de Monte Alto, Monte Alto, Brazil; MNN: Muséum National du Niger, Niamey, Niger; MUC-PV: Museo de la Universidad Nacional del Comahue, Neuquén, Argentina; MZSP-PV: Museu de Zoologia, Universidade de São Paulo, São Paulo, Brazil; RCL: Museu de Ciências Naturais, Pontifícia Universidade Católica de Minas Gerais, Brazil; ROM: Royal Ontario Museum, Toronto, Canada; UA: University of Antananarivo, Madagascar; UFRJ: Museu de Paleontologia e Estratigrafia, Universidade Federal de Rio de Janeiro, Rio de Janeiro, Brazil; URC: Universidade Estadual Paulista, Rio Claro, Brazil; ZDM: Zigong Dinosaur Museum, Zigong, China.

### Nomenclatural acts

The electronic edition of this article conforms to the requirements of the amended International Code of Zoological Nomenclature, and hence the new names contained herein are available under that Code from the electronic edition of this article. This published work and the nomenclatural acts it contains have been registered in ZooBank, the online registration system for the ICZN. The ZooBank LSIDs (Life Science Identifiers) can be resolved and the associated information viewed through any standard web browser by appending the LSID to the prefix “http://zoobank.org/”. The LSID for this publication is: urn:lsid:zoobank.org:pub:32AF6BF2-7D3A-480D-AA35-070BF84380C1. The electronic edition of this work was published in a journal with an ISSN, and has been archived and is available from the following digital repositories: PubMed Central, LOCKSS.

## Results

### Systematic paleontology

Crocodylomorpha Walker, 1970 (*sensu* Clark, 1986)

Crocodyliformes Hay, 1930 (*sensu* Clark, 1986)

Sphagesauridae Khun, 1968

*Caipirasuchus* Iori & Carvalho, 2011 [11]

#### Type species

*Caipirasuchus paulistanus* Iori & Carvalho, 2011 [11]

#### Emended diagnosis

Sphagesaurid crocodyliforms that differ from other members of the clade by the following unique combination of characters (unambiguous synapomorphies of the genus not found in other crocodyliforms are marked with an asterisk): presence of antorbital fenestra and fossa, relatively narrow and elongated rostrum, antorbital process of jugal with a moderately developed longitudinal ridge that lacks a distinct depression underneath it, transversely oriented groove on dorsal surface of postorbital interrupting the anterolateral rim of the supratemporal fossa, jugal anteroventral end with a short triangular process that wedges between the ectopterygoid and maxilla at the level of the orbits*, highly developed crest on posterior surface of the quadrate, anterior half of the symphyseal region lateromedially narrow and anteroposteriorly elongated (three times as long as wide*), implantation of lower incisiviforms in a continuous alveolar groove, smooth enamel surface in anterior teeth and posterior teeth with well developed apicobasal carinae and rugose enamel surface (‘pebbled enamel’ *sensu* [8]), small diastema in dentary between 5^th^ and 6^th^ dentary tooth. Modified from Iori *et al.* [12] (see Comments below).

#### Occurrence

All the known material comes from the Bauru Group, in different localities in the state of São Paulo, Brazil. The remains come from sediments referred to the Adamantina Formation although the different outcrops that produced remains of the different species of *Caipirasuchus* cannot be confidently correlated.

#### Taxonomic content

We include in this genus the type species *C. paulistanus* [11], the new species described in this contribution, and the taxon originally described as *Sphagesaurus montealtensis* [10]. The referral of the latter species to *Sphagesaurus* (a genus that was at that time monospecific and the only member of the family Sphagesauridae) was a reasonable choice by Andrade & Bertini [10]. However, in recent years five other taxa have been described and referred to the family Sphagesauridae (for which five different monotypic genera had been erected; see above). As recently noted by Iori *et al.* [12] *Caipirasuchus paulistanus* is remarkably similar in many features to *Sphagesaurus montealtensis* and both were retrieved as sister taxa in a recent phylogenetic analysis [12]. The new taxon described in this contribution also is remarkably similar to these two other taxa in its overall morphology and the three species form a monophyletic clade within Sphagesauridae in our phylogenetic analysis (see below), separated from the type species of *Sphagesaurus*. We therefore agree in the generic referral of “*Sphagesaurus*” *montealtensis* to the genus *Caipirasuchus* proposed by Iori *et al.* [12]. The monophyly of the clade formed by *Caipirasuchus paulistanus*, *Caipirasuchus montealtensis*, and the new taxon, coupled with the marked overall similarity of these three species are the basis for referring these three taxa to the same genus (which is the least disruptive nomenclatural change required to match the taxonomy with the phylogenetic results). The new information provided by the specimen described here requires revising the diagnosis of the three species. We provide below an emended diagnosis for the two previously published species (*C. paulistanus* and *C. montealtensis*) and then for the new species described in this contribution.

#### Comments

Iori *et al.* [12] have recently provided a diagnosis of the genus based on their phylogenetic analysis and the information for the two previously described species of this genus (*Caipirasuchus paulistanus* and *Caipirasuchus montealtensis*). The information provided by the new species and our phylogenetic analysis prompted us to revise the diagnosis of the genus, adding characters that were absent from their diagnosis [12] and deleting characters that are not diagnostic of the genus *Caipirasuchus*. Three characters regarded as synapomorphies by Iori *et al.* [12] have been excluded from the diagnosis given that the new species lack this morphology: 1) external nares bordered only by the premaxilla (see comments below on this character for *C. paulistanus*), 2) premaxilla with four teeth and a diastema between 3^rd^ and 4^th^ teeth, 3) nasal with a groove parallel to the suture with frontal. Furthermore, they regarded the presence of a diastema in the lower jaw between the 4^th^ and 5^th^ teeth as diagnostic of the genus, but this condition is also present in the closely related taxon *Yacarerani boliviensis*.

*Caipirasuchus paulistanus* Iori & Carvalho, 2011 [11]

(Figures 1–5)

**Figure 1:**
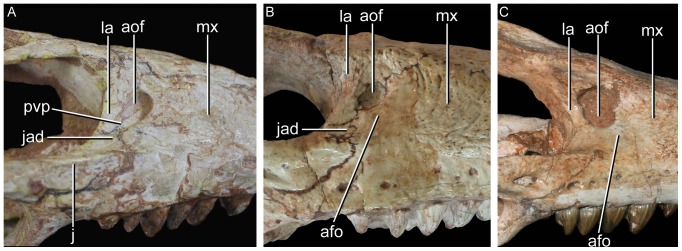
Antorbital region of the three species of *Caipirasuchus* in right lateral view. **A**, *Caipirasuchus paulistanus* MPMA 67-0001/00; **B**, *Caipirasuchus montealtensis* MPMA 15-001/90; **C**, *Caipirasuchus stenognathus* MZSP-PV 139. Abbreviations: **afo**, antorbital fossa; **aof**,antorbital fenestra; **j**, jugal; **jad**, jugal anterodorsal process; **la**, lacrimal; **mx**, maxilla; **pvp**, posteroventral projection of antorbital fenestra.

**Figure 2:**
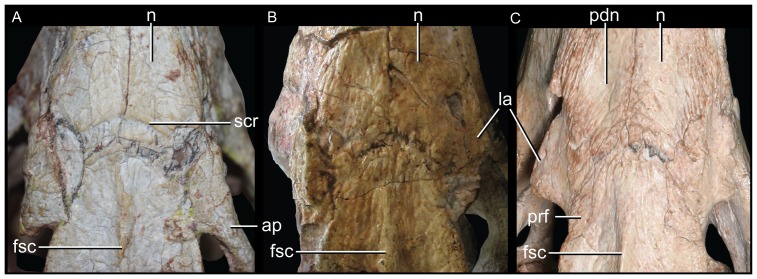
Nasal-frontal-prefrontal of the three species of *Caipirasuchus* in dorsal view. **A**, *Caipirasuchus paulistanus* MPMA 67-0001/00; **B**, *Caipirasuchus montealtensis* MPMA 15-001/90; **C**, *Caipirasuchus stenognathus* MZSP-PV 139. Abbreviations: **fr**, frontal; **fsc**, frontal sagittal crest; **la**, lacrimal; **n**, nasal; **ap**, anterior palpebral; **pdn**, posterolateral depression of nasals; **prf**, prefrontal; **scr**, semicircular ridge of nasals.

**Figure 3:**
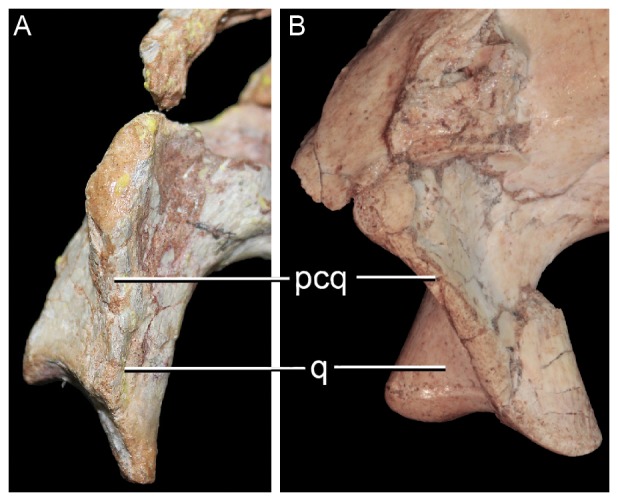
Distal body of quadrate of two species of *Caipirasuchus* in posterior view. **A**, *Caipirasuchus paulistanus* MPMA 67-0001/00; **B**, *Caipirasuchus stenognathus* MZSP-PV 139. Abbreviations: **fae**, foramen aerum; **pqc**, posterior quadrate crest; **q**, quadrate.

**Figure 4:**
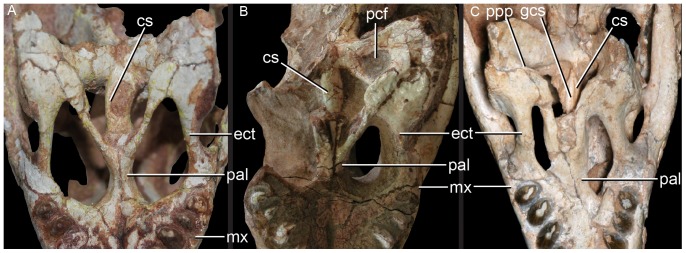
Choanal region of the three species of *Caipirasuchus* in ventral view. **A**, *Caipirasuchus paulistanus* MPMA 67-0001/00; **B**, *Caipirasuchus montealtensis* MPMA 15-001/90; **C**, *Caipirasuchus stenognathus* MZSP-PV 139. Abbreviations: **cs**, choanal septum; **ect**, ectopterygoid; **gcs**, groove of choanal septum; **mx**, maxilla; **pal**, palatine; **pcf**, parachoanal fossa; **ppp**, pterygoid platform for palatine; **pt**, pterygoid.

**Figure 5:**
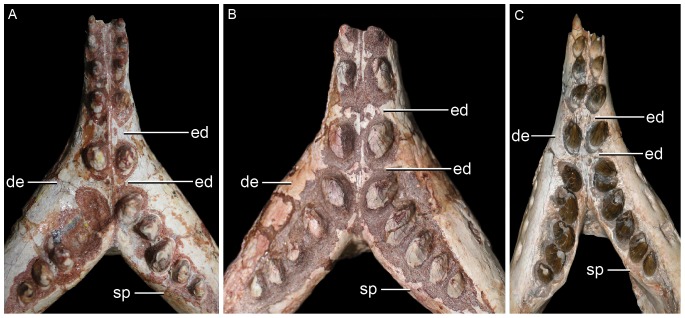
Mandibular symphysis of the three species of *Caipirasuchus* in dorsal view. **A**, *Caipirasuchus paulistanus* MPMA 67-0001/00; **B**, *Caipirasuchus montealtensis* MPMA 15-001/90; **C**, *Caipirasuchus stenognathus* MZSP-PV 139. Abbreviations: **de**, dentary; **ed**, edentulous spaces (diastemata sensu [11]); **spl**, splenial.

#### Emended diagnosis

Sphagesaurid crocodyliform that differs from other species of the genus by the following unique combination of characters (autapomorphies marked with asterisk): antorbital fenestra dorsoventrally elongated, with a distinct posteroventral projection*, posterior region of the nasal with posteriorly concave ridge*, frontal with sagittal crest reaching anterior end of the bone, elongated distal body of quadrates twice as dorsoventrally high as lateromedially broad (with medial condyle extremely elongated ventrally), anterior end of palatines markedly flared at its anterior contact with palatal branches of maxilla, absence of anteroposterior constriction at medial origin of pterygoid flanges (lateral to the choanal groove), absence of deep parachoanal fossa, well-developed edentulous spaces between first lower molariform and anterior set of incisiviforms and posterior set of molariforms. Modified from Iori *et al.* [12].

#### Holotype

MPMA 67-0001/00. Relatively complete skull, lacking only most of occipital region, and almost complete mandible with poorly preserved posterior region of mandibular rami.

#### Comments

The diagnoses provided by Iori & Carvalho [11] and Iori *et al.* [12] required modifications given that the new taxon shows some characters are no longer autapomorphic of *C. paulistanus* and/or because of they are shared with other advanced notosuchians. We have focused in our emended diagnosis on characters that distinguish *C. paulistanus* from the two other species of *Caipirasuchus*, as well as from other sphagesaurids. The modified diagnosis include the distinctive posteroventral projection of the antorbital fenestra (also noted by Iori *et al.* [12]; see Figure 1) and the posteriorly concave semicircular ridge on the posterior margin of the nasals (Figure 2), two autapomorphic characters that are absent in all other notosuchians. These and other features (see below) justify the validity of this species, as stated by Iori *et al.* [12] but contrasting the recent suggestion that this taxon is indistinguishable from *Sphagesaurus montealtensis* [3].

A conspicuous autapomorphic feature of *C. paulistanus* included in the original diagnosis [11] is the exclusion of the nasal from the dorsal margin of the external nares by an anterodorsal projection of the premaxilla. The type specimen of this taxon (MPMA 67-0001/00), however, provides only ambiguous information regarding the presence of this character. The right premaxilla lacks this anterodorsal process and the right nasal therefore forms part of the dorsal margin of the external nares. On the left side, there is a small piece of bone located on the left dorsolateral corner of the external nares. This fragment of bone was originally identified as the anterodorsal process of the premaxilla [11] but is actually separated from both the left nasal and the left premaxilla by a crack infilled with sediment. Therefore, we cannot confirm at the moment the existence of such premaxillary process and the consequent exclusion of the nasals from the external nares, which would be an autapomorphic feature for a notosuchian crocodyliform. Iori *et al.* [12] have also included three characters in the diagnosis of *C. paulistanus* that are not considered here as such. The rostral lateral wall with an abrupt transition to the dorsal surface and the ventrally projected pterygoid flanges are two features that are present in the new species described here (and also in other crocodyliforms). Finally, the lanceolate supraorbital fenestra (a name used by Iori *et al.* [12] to the space between the palpebral and the frontal orbital margin) is a generalized condition present in other notosuchian crocodyliforms.

Other features of the diagnosis distinguish *C. paulistanus* from *C. montealtensis* and/or the new taxon. The frontal sagittal crest reaches the anterior end of the bone in *C. paulistanus* whereas in the two other species the crest disappears along the anterior third of the frontal (Figure 2). The distal body of the quadrate (distal to the entrance for the cranioquadrate passage) is much more elongated in *C. paulistanus* than in the specimen described here in which this region is as dorsoventrally high as lateromedially broad (Figure 3). This condition is unknown in *C. montealtensis*. The anterior end of palatines are much more flared in *C. paulistanus* at its anterior contact with palatal branches of maxilla and the pterygoid flanges are unconstricted at its medial origin (Figure 4), whereas in the other species the medial origin of pterygoid flanges is markedly narrow lateral to the choanal groove with respect to the lateral end of the flanges. Finally, the edentulous spaces that separate the first lower molariform from the anterior set of incisiviforms and the posterior set of molariforms (diastemata *sensu* [11]) are more extensive in *C. paulistanus* than in the other species of the genus (Figure 5).

*Caipirasuchus montealtensis* (Andrade & Bertini, 2008) [10]

(Figures 1–2, 4–5)

#### Synonymy

*Sphagesaurus montealtensis* (Andrade & Bertini, 2008) [10]

#### Emended diagnosis

Sphagesaurid crocodyliform that differs from other species of the genus by the following unique combination of characters (autapomorphies marked with asterisk): longitudinal sagittal sulcation over the palate surface with palatal rami of maxilla inclined forming a V-shaped (ventrally concave) secondary palate, pterygoid flanges bearing a single, rounded, and deep parachoanal fossa*, anteroposterior constriction at the medial origin of pterygoid flanges (lateral to the choanal groove), anterior end of ectopterygoid not forming part of the posterior margin of last maxillary alveolus. Modified from Iori *et al.* [12].

#### Holotype

MPMA 15-001/90. Relatively complete skull lacking the anterior region of the premaxilla and posterior left region and most of the occiput; mandible preserved from the anterior region of the symphysis to the level of the external mandibular fenestra. The dentition has been damaged and most of the enamel coat has not been preserved in the upper and lower toothrows.

#### Comments

As also noted by Iori *et al.* [12] the description of this taxon as *Sphagesaurus montealtensis* [10] was done when the only other known sphagesaurid was *S. huenei*. Therefore, the original diagnosis of this species was mostly based to distinguish it from the skull material referred to *S. huenei* (RCL-100) [5]. Several characters identified as autapomorphies of “*Sphagesaurus*” *montealtensis* by Andrade & Bertini [10] turned out to be synapomorphic of the genus *Caipirasuchus*, whereas others were present in a broader group of advanced notosuchians. The emended diagnosis recently given by Iori *et al.* [12] included two characters. One of them is recognized here as autapomorphic (parachoanal fossa) but the other (small and subcircular antorbital fenestra) is present in the new species as well as in other notosuchians and therefore has been excluded from the emended diagnosis.

The most conspicuous character that distinguishes this taxon from the other two species of *Caipirasuchus* is a character noted by previous authors [10], [12]: the presence of a single, rounded, and deep parachoanal fossa located on the ventral surface of the pterygoid flanges, posteromedial to the palatine-pterygoid contact (Figure 4). The ventral surface of the pterygoid flanges of *C. paulistanus* and the new species described here is flat or only slightly concave, differing from the distinct and well-developed fossa present in *C. montealtensis*. This difference adds to the ones mentioned above, justifying the recognition of *C. montealtensis* and *C. paulistanus* as two distinct taxa (in agreement with [12] but *contra* [3]). The presence of a parachoanal fossa has been also noted for *S. huenei* (RCL-100) although in the latter taxon is located much more medially on the pterygoids, closer to the choanal groove [5], [10]. Additionally, baurusuchids commonly have multiple parachoanal fossae [30], but none of them has the same position and shape as in *C. montealtesis*.

Andrade & Bertini [10] also noted the presence of a longitudinal sulcation of the palatal rami of maxilla, which are set obliquely to the transversal plane of the skull and form a secondary palate. This condition is most notorious towards the posterior end of the palatal branches of the maxilla and in the two other species this sulcation is either absent or poorly developed. We have left this character in the diagnosis although it must be noted that it may be affected by preservational causes. Another difference found with respect to the two other species is that the anterior end of ectopterygoid does not form part of the posteriormost maxillary alveolus in *C. montealtesis* (as in *S. huenei*) but it reaches the alveolar margin in the two other species of *Caipirasuchus* (Figure 4).

*Caipirasuchus stenognathus* Pol, Nascimento, Carvalho, Riccomini, Pires-Domingues & Zaher sp. nov. urn:lsid:zoobank.org:act:6378E430-1749-4C0E-97ED-616439DC029A

(Figures 1–30)

**Figure 6:**
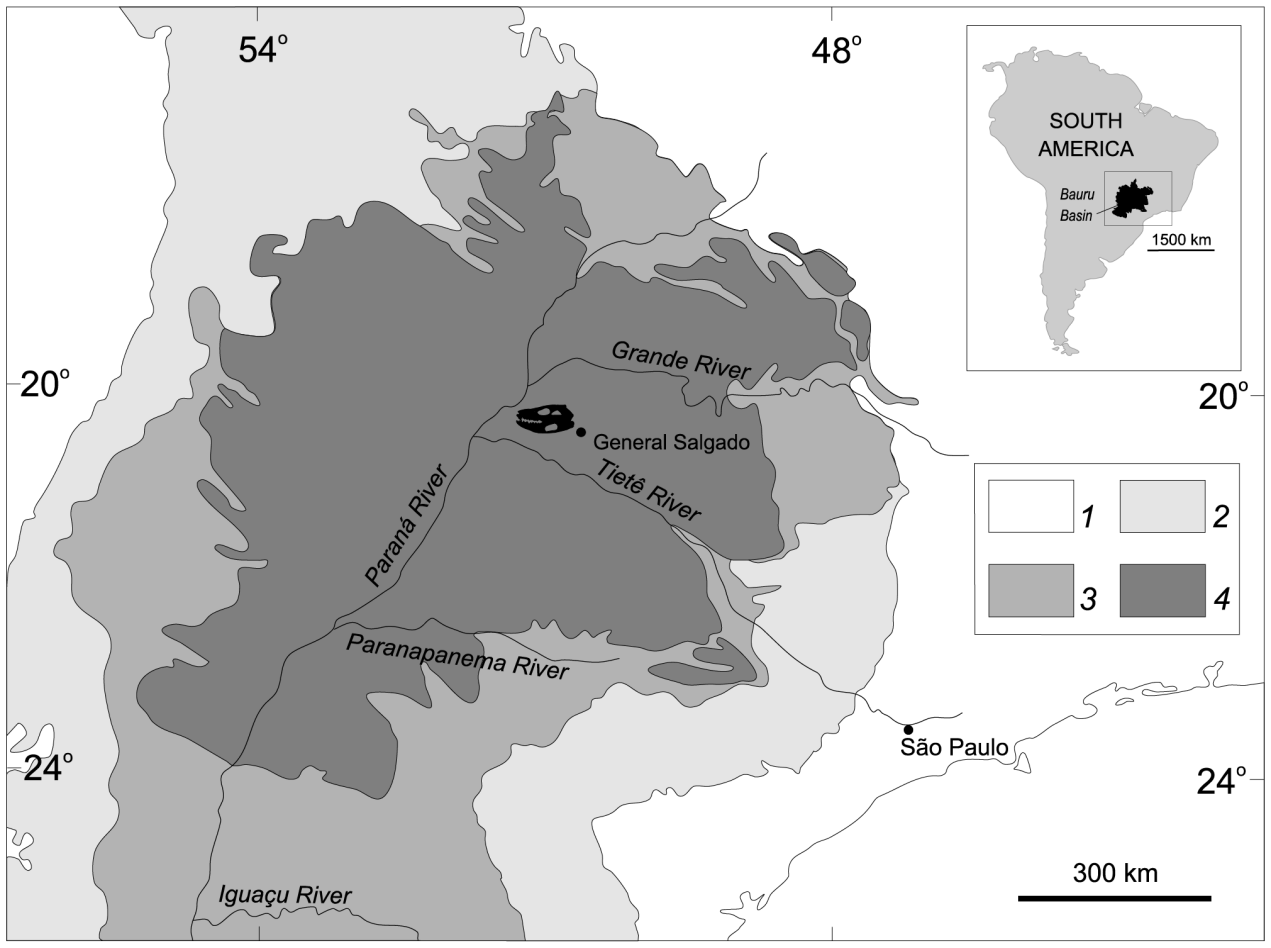
Location of the *Caipirasuchus stenognathus* occurrence in the Bauru Basin (after [35], modified). Legend for the numbers are as follow: 1 - Precambrian basement rocks; 2 - Paraná Basin (Ordovician to Triassic); 3 - Serra Geral Formation and Caiuá Basin (Early Cretaceous); 4 - Bauru Basin (Late Cretaceous).

**Figure 7:**
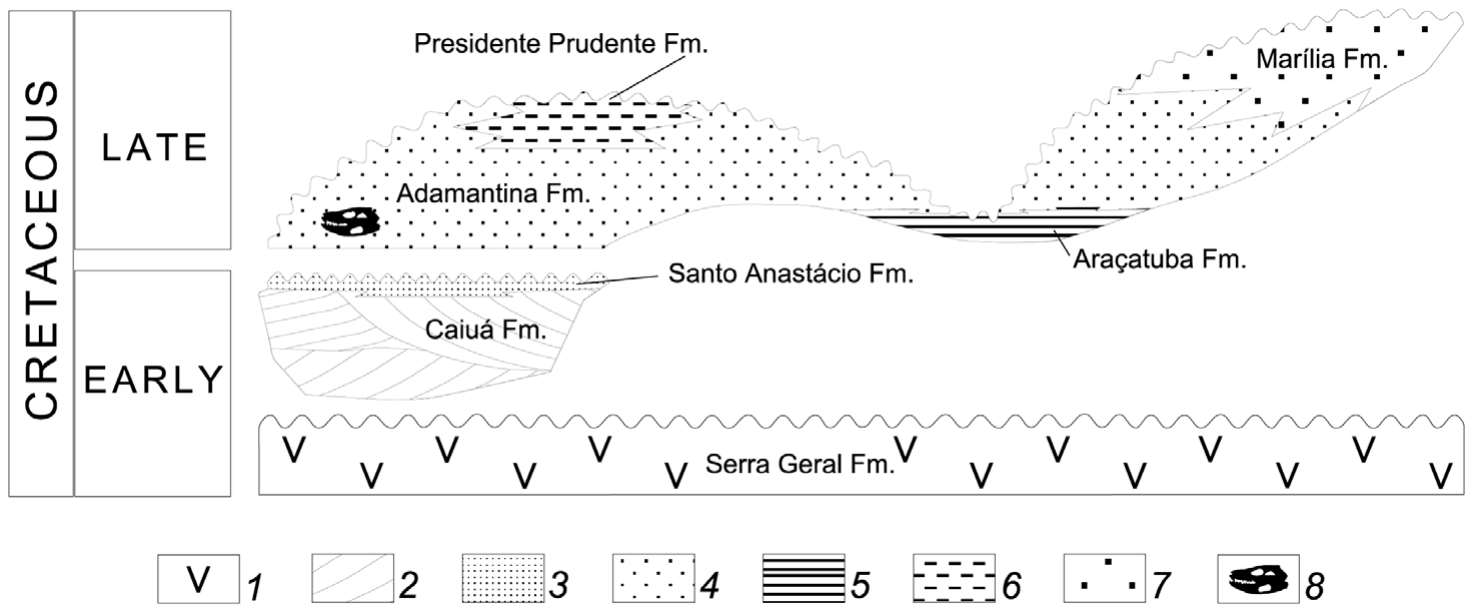
Stratigraphic relationships of the Bauru Group in the Bauru Basin (after [35], modified). Legend for the numbers are as follow: 1 - basaltic rocks; 2 - cross-bedded sansdstone; 3 - massive to slightly stratified sandstone; 4 - massive to slightly stratified sandstone interlayered with mudstones; 5 - sandstone, siltstone and mudstone; 6 - sandstone and mudstone; 7 - sandstone and conglomerate with calcite cement; 8 - position of *Caipirasuchus stenognathus* remains.

**Figure 8:**
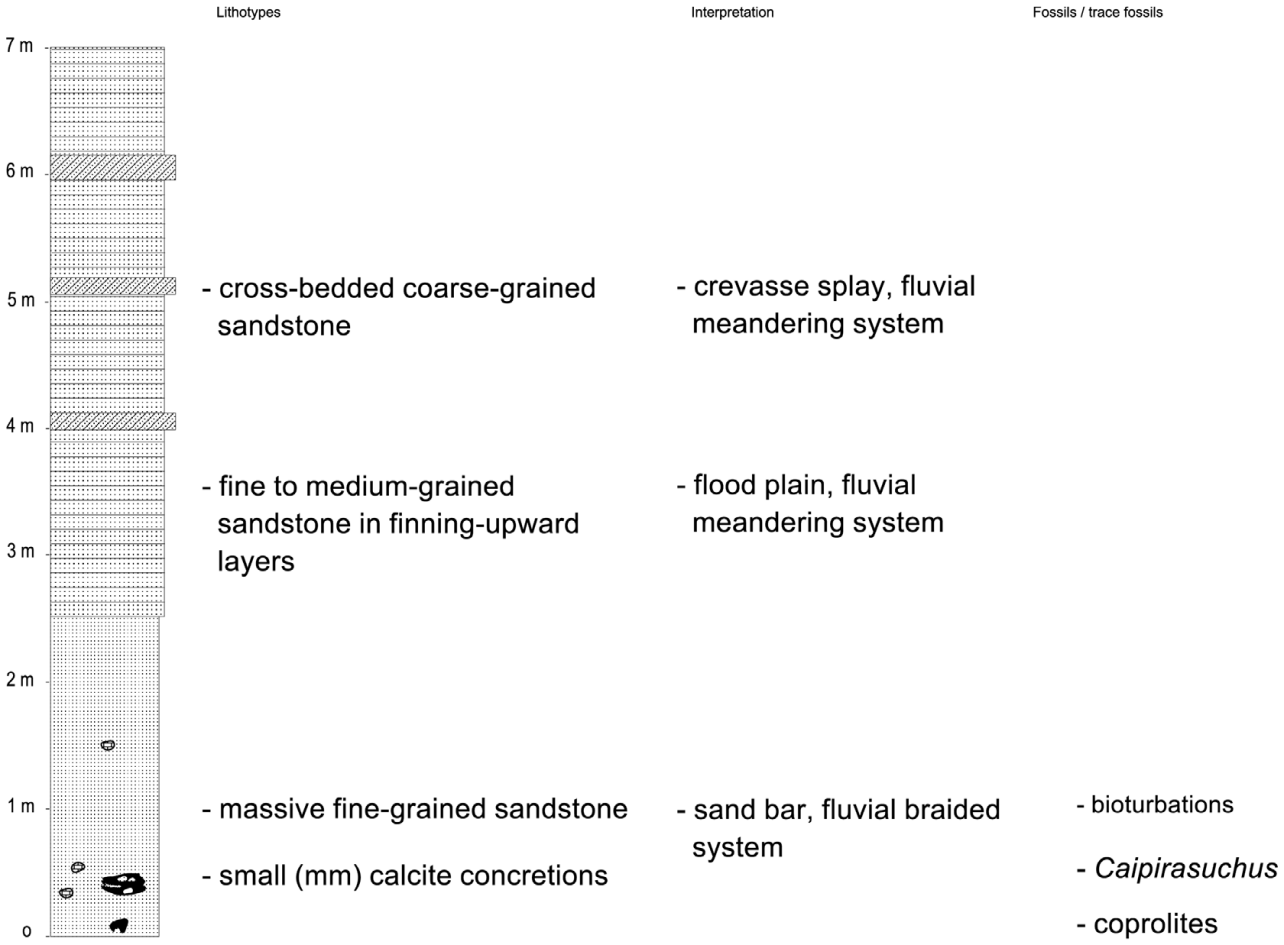
Composite columnar section of the site with fossil remains. Adamantina Formation, 13ão Paulo State, Brazil (coordinates: 20°34′1.44″S, 50°27′49.89″W).

**Figure 9:**
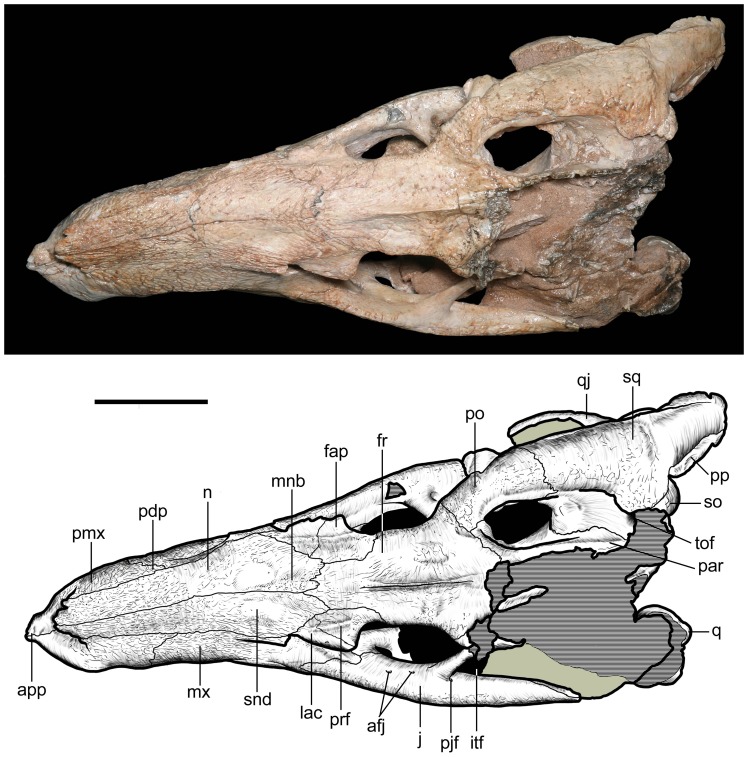
Skull of *Caipirasuchus stenognathus* (MZSP-PV 139) in dorsal view. Abbreviations: **afj**, anterior foramina on medial surface of jugal; **app**, anterodorsal process of premaxilla; **fap**, articular facet for anterior palpebral; **fr**, frontal; **itf**, infratemporal fenestra; **j**, jugal; **lac**, lacrimal; **mnb**, median nasal bulge; **mx**, maxilla; **n**, nasal; **par**, parietal; **pdp**, posterodorsal process of premaxilla; **pjf**, posterior jugal foramen; **pmx**, premaxilla; **po**, postorbital; **pp**, paroccipital process; **prf**, prefrontal; **q**, quadrate; **qj**, quadratojugal; **snd**, smooth nasal depression; **so**, supraoccipital; **sq**, squamosal; **tof**, temporo-orbital foramen. Scale bar equals 2.5 cm.

**Figure 10:**
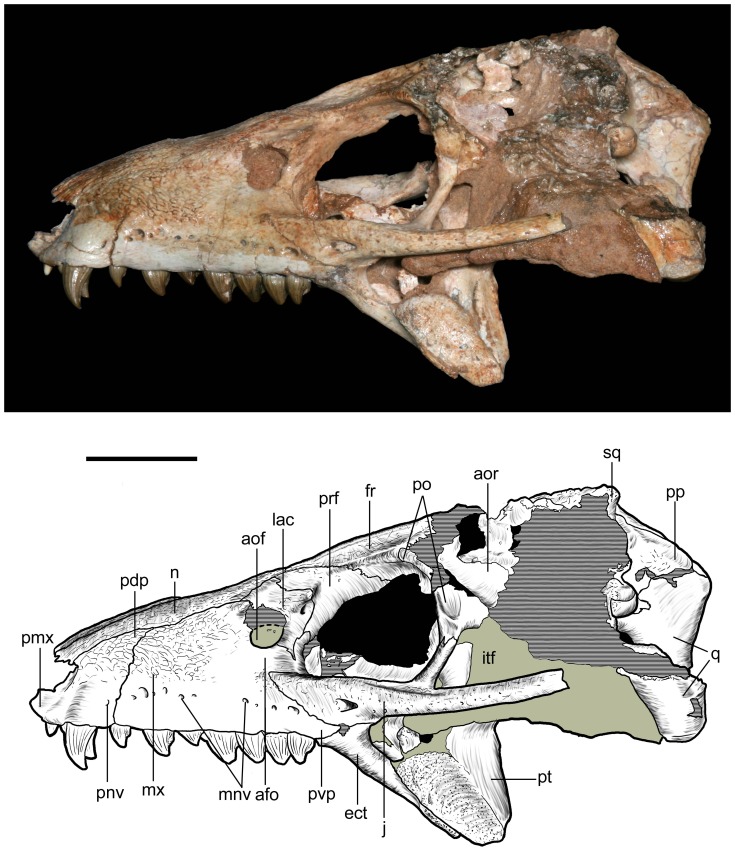
Skull of *Caipirasuchus stenognathus* (MZSP-PV 139) in left lateral view. Abbreviations: **afo**, antorbital fossa; **aof**, antorbital fenestra; **aor**, anterior end of otic recess; **ect**, ectopterygoid; **fr**, frontal; **itf**, infratemporal fenestra; **j**, jugal; **lac**, lacrimal; **mnv**, maxillary neurovascular foramina; **mx**, maxilla; **n**, nasal; **pdp**, posterodorsal process of premaxilla; **pmx**, premaxilla; **pnv**, premaxillary neurovascular foramen; **po**, postorbital; **pp**, paroccipital process; **prf**, prefrontal; **pt**, pterygoid; **pvp**, posteroventral process of maxilla; **q**, quadrate; **sq**, squamosal. Scale bar equals 2.5 cm.

**Figure 11:**
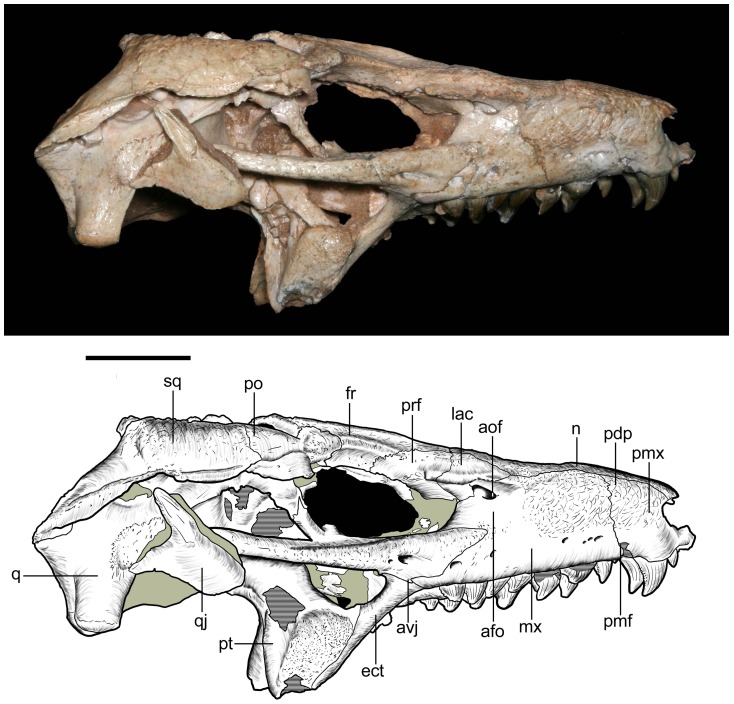
Skull of *Caipirasuchus stenognathus* (MZSP-PV 139) in right lateral view. Abbreviations: **afo**, antorbital fossa; **aof**, antorbital fenestra; **avj**, anteroventral process of jugal; **ect**, ectopterygoid; **fr**, frontal; **lac**, lacrimal; **mx**, maxilla; **n**, nasal; **pdp**, posterodorsal process of premaxilla; **pmf**, premaxillo-maxillary neurovascular foramen; **pmx**, premaxilla; **po**, postorbital; **prf**, prefrontal; **pt**, pterygoid; **q**, quadrate; **qj**, quadratojugal; **sq**, squamosal. Scale bar equals 2.5 cm.

**Figure 12:**
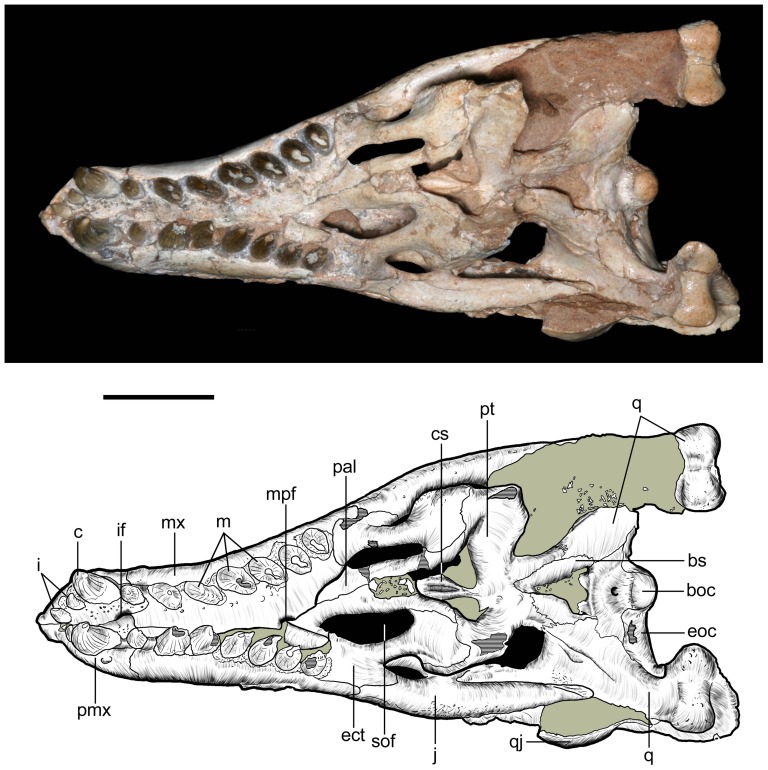
Skull of *Caipirasuchus stenognathus* (MZSP-PV 139) in ventral view. Abbreviations: **boc**, basioccipital; **bs**, basisphenoid; **c**, caniniform; **cs**, choanal septum; **ect**, ectopterygoid; **eoc**, exoccipital; **i**, incisiviform; **if**, incisive foramen; **j**, jugal; **m**, molariform; **mpf**, maxillo-palatine fenestra; **mx**, maxilla; **pal**, palatine; **pmx**, premaxilla; **pt**, pterygoid; **q**, quadrate; **qj**, quadratojugal; **sof**, suborbital fenestra. Scale bar equals 2.5 cm.

**Figure 13:**
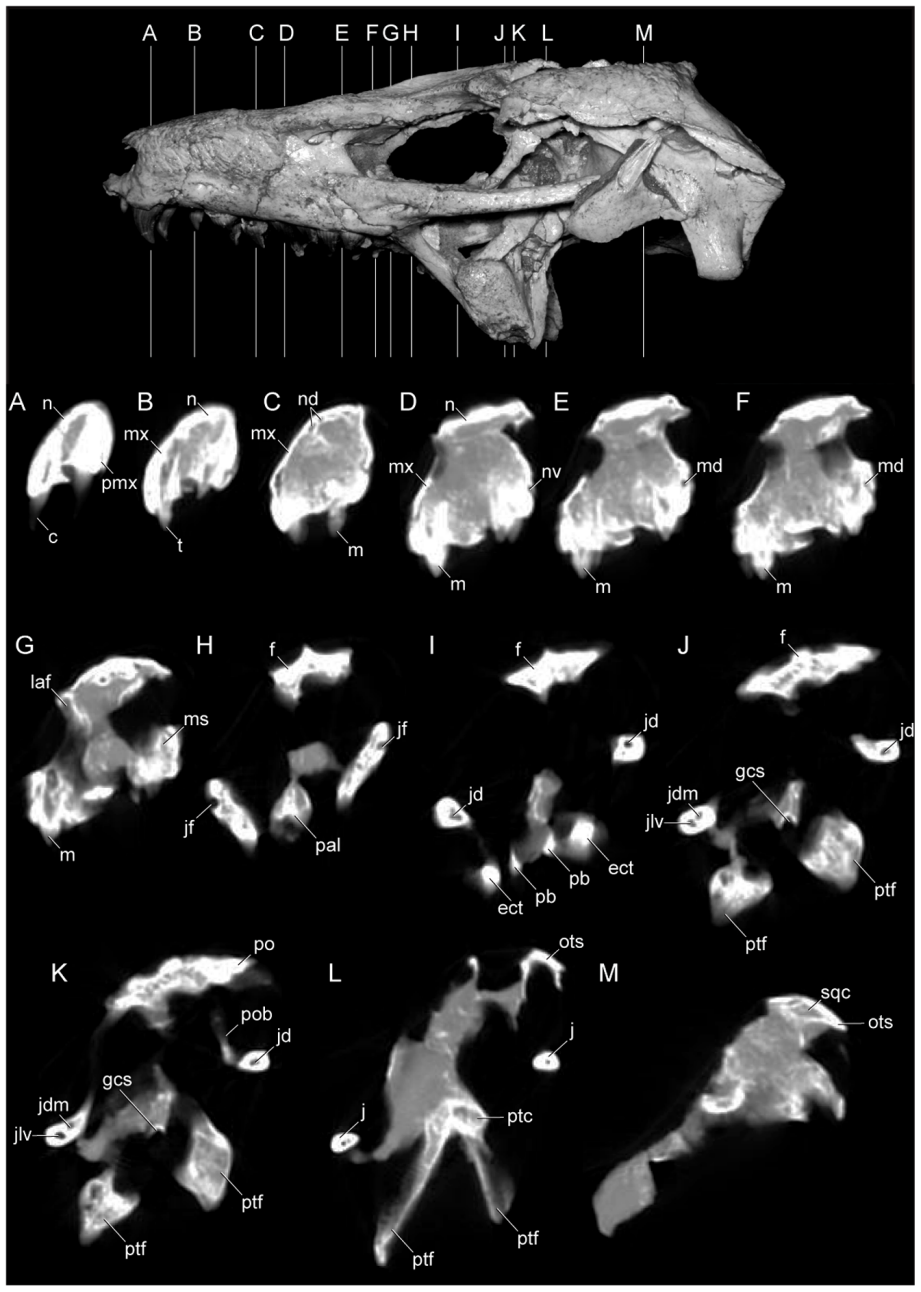
Transversal CT slices of the skull of *Caipirasuchus stenognathus* (MZSP-PV 139) with reference image of the skull showing the placement of the slices A–M. Abbreviations: **c**, caniniform tooth; **ect**, ectopterygoid; **f**, frontal; **gcs**, grooved choanal septum; **j**, jugal; **jd**, jugal neurovascular duct; **jdm**, jugal dorsomedial duct; **jf**, jugal foramen; **jlv**, jugal lateroventral duct; **laf**, lacrimal foramen; **m**, molariform tooth; **md**, maxillary neurovascular duct; **ms**, maxillary neurovascular sinus; **mx**, maxilla; **n**, nasal; **nd**, nasal neurovascular duct; **nv**, neurovascular foramen; **ots**, otic shelf; **pal**, palatine; **pb**, palatine bar; **pmx**, premaxilla; **po**, postorbital; **pob**, postorbital bar; **ptc**, pterygoid internal cavity; **ptf**, pterygoid flange; **sqc**, squamosal internal cavity; **t**, transitional tooth. Scale bar equals 2.5 cm.

**Figure 14:**
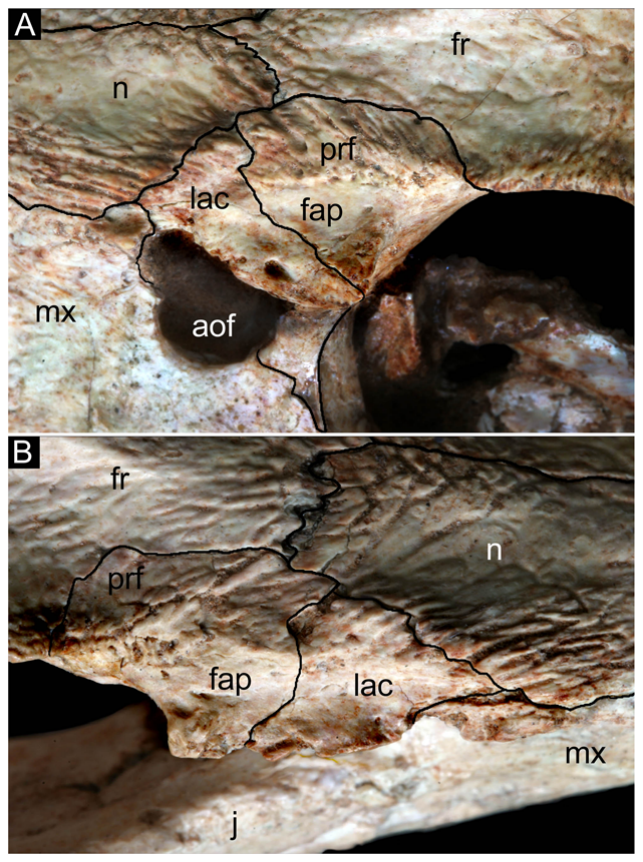
Posterolateral region of the dorsal surface of the rostrum of *Caipirasuchus stenognathus* (MZSP-PV 139). A , left side; **B**, right side. Abbreviations: **aof**, antorbital fenestra; **fap**, articular facet for anterior palpebral; **fr**, frontal; **j**, jugal; **lac**, lacrimal; **mx**, maxilla; **n**, nasal; **prf**, prefrontal.

**Figure 15:**
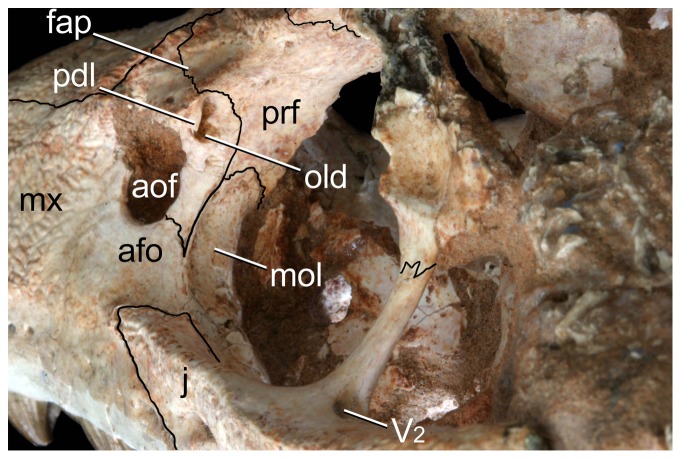
Anterodorsal region of the orbit of *Caipirasuchus stenognathus* (MZSP-PV 139) in posterolateral view. Abbreviations: **afo**, antorbital fossa; **aof**, antorbital fenestra; **fap**, articular facet for anterior palpebral; **j**, jugal; **lac**, lacrimal; **mol**, maxillary orbital lamina; **mx**, maxilla; **old**, posterior opening of lacrimal duct; **pdl**, posterodorsal process of lacrimal; **prf**, prefrontal; **V_2_**, foramen for the entrance of maxillary branch of the trigeminal cranial nerve.

**Figure 16:**
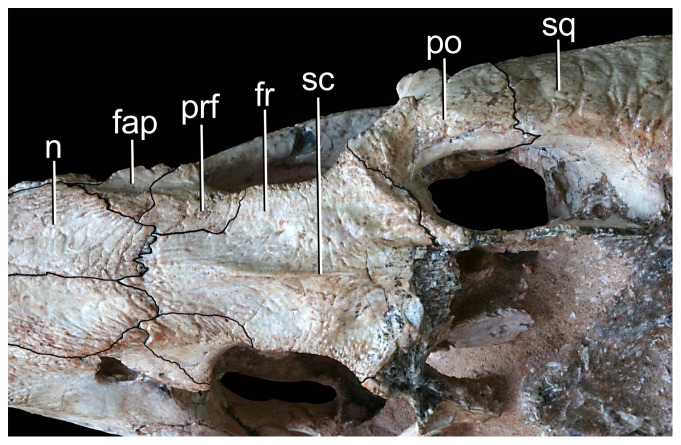
Interorbital region of *Caipirasuchus stenognathus* (MZSP-PV 139) in dorsal view. Abbreviations: **fap**, articular facet for anterior palpebral; **fr**, frontal; **n**, nasal; **po**, postorbital; **prf**, prefrontal; **sc**, sagittal crest; **sq**, squamosal.

**Figure 17:**
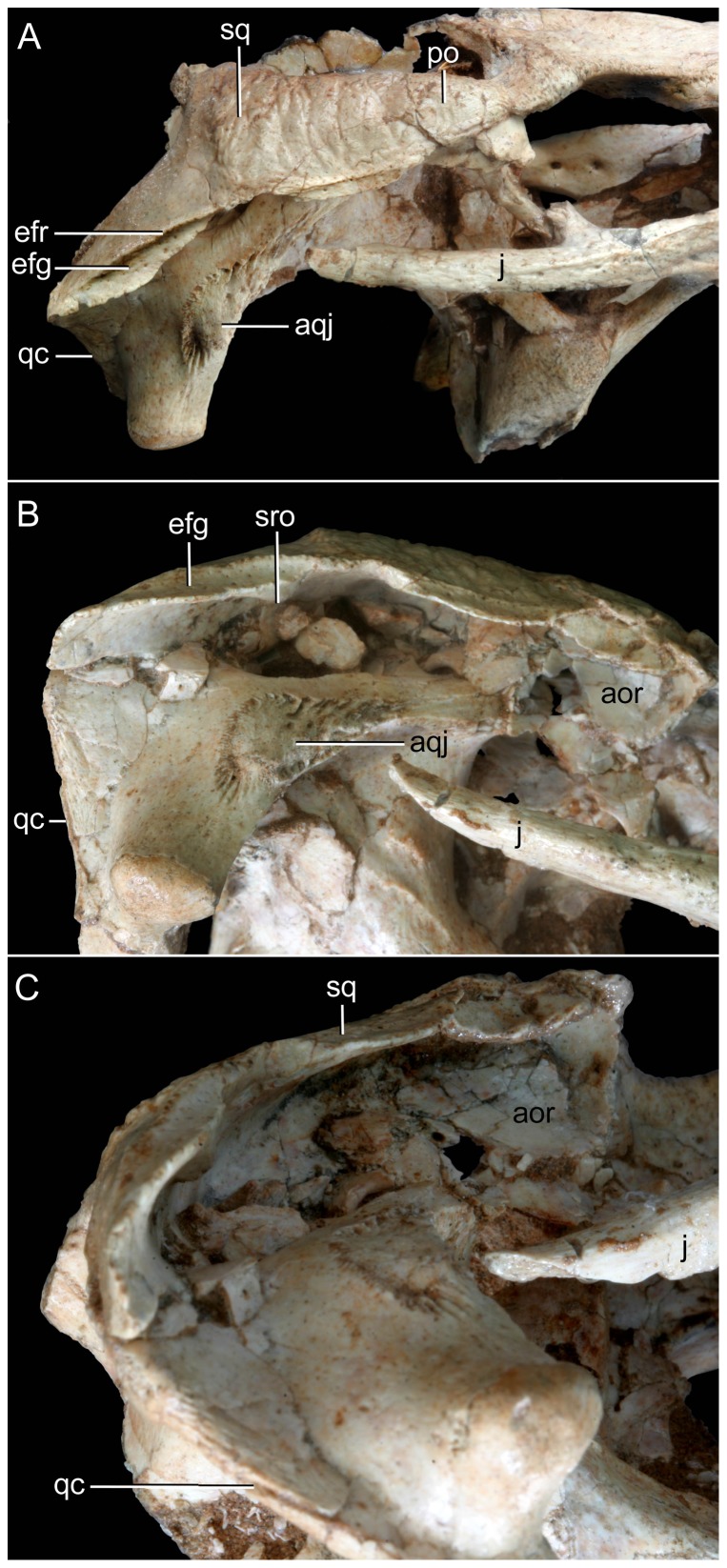
Right otic recess of *Caipirasuchus stenognathus* (MZSP-PV 139). A , lateral view; **B**, ventrolateral view; **C**, posteroventral view. Abbreviations: **aor**, anterior end of otic recess; **aqj**, articular facet for quadratojugal; **efg**, ear-flap groove; **efr**, ear-flap ridge; **j**, jugal; **ota**, otic aperture; **po**, postorbital; **pof**, periotic fossa; **qc**, quadrate posterior crest; **qj**, quadratojugal; **sq**, squamosal; **sro**, squamosal ridge of otic recess.

**Figure 18:**
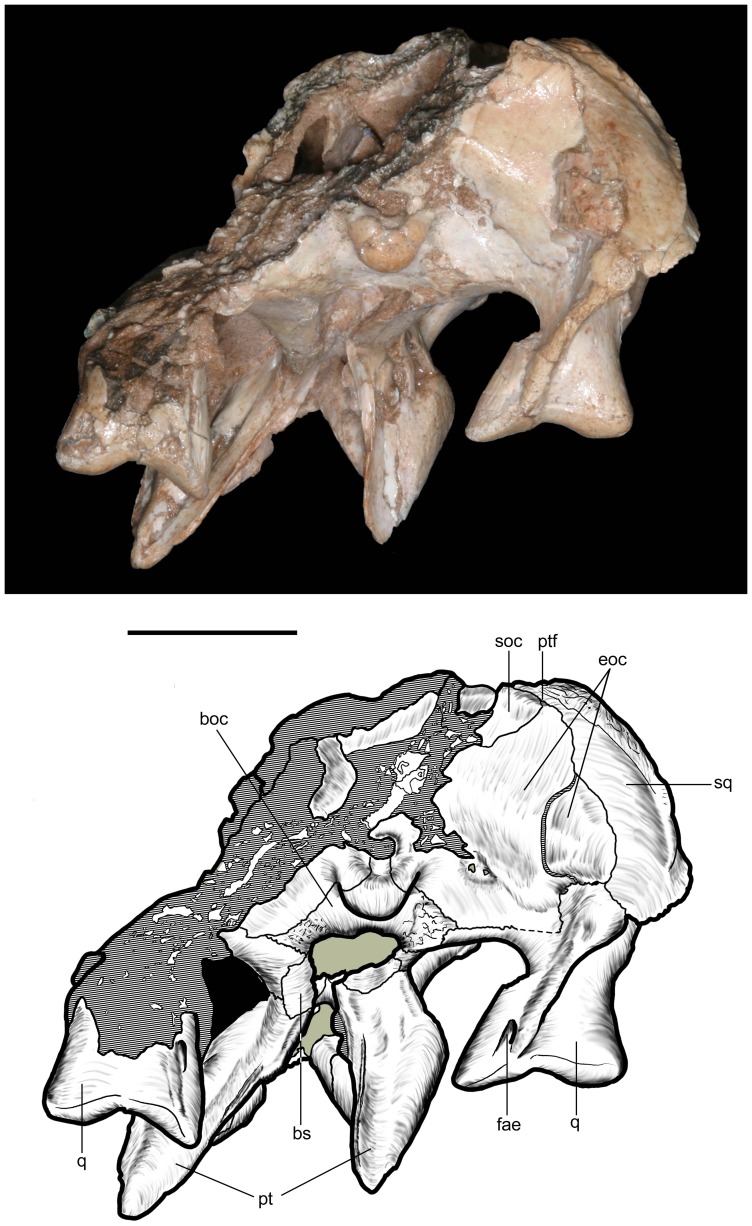
Occipital region of *Caipirasuchus stenognathus* (MZSP-PV 139). Abbreviations: **boc**, basioccipital; **bs**, basisphenoid; **eoc**, exoccipital; **fae**, foramen aërum; **pt**, pterygoid; **ptf**, post-temporal fenestra; **q**, quadrate; **soc**, supraoccipital; **sq**, squamosal. Scale bar equals 2.5 cm.

**Figure 19:**
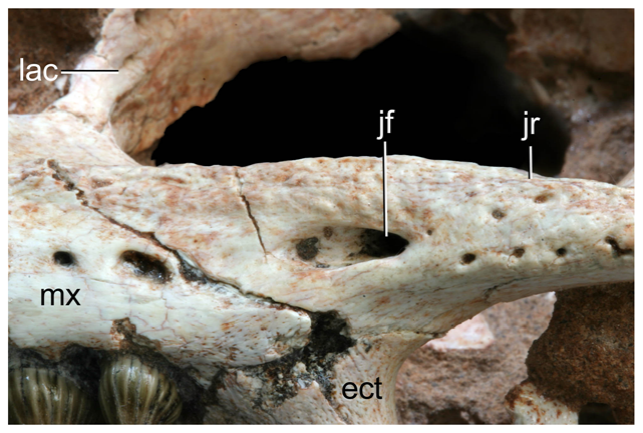
Anteroventral orbital region of *Caipirasuchus stenognathus* (MZSP-PV 139) in lateral view. Abbreviations: **ect**, ectopterygoid; **jf**, jugal foramen; **jr**, jugal ridge; **lac**, lacrimal; **mx**, maxilla.

**Figure 20:**
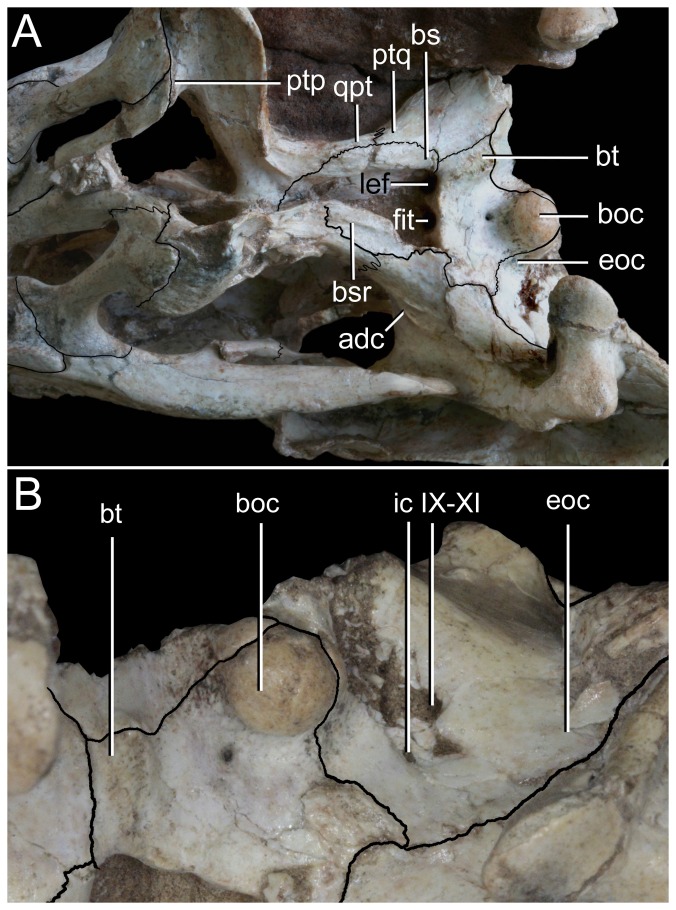
Basioccipital-basisphenoid region of *Caipirasuchus stenognathus* (MZSP-PV 139). **A**, ventral view; **B**, posteroventral view. Abbreviations: **adc**, adductor crests; **boc**, basioccipital; **bs**, basisphenoid; **bsr**, basisphenoid ridge; **bt**, basal tubera; **eoc**, exoccipital; **fit**, foramen intertympanicum; **ic**, internal carotid foramen; **IX-XI**, passage for the cranial nerves IX-XI (foramen vagi); **lef**, lateral Eustachian foramen; **ptp**, pterygoid platform for palatine bar; **ptq**, pterygoid process of the quadrate; **qpt**, quadrate process of pterygoid.

**Figure 21:**
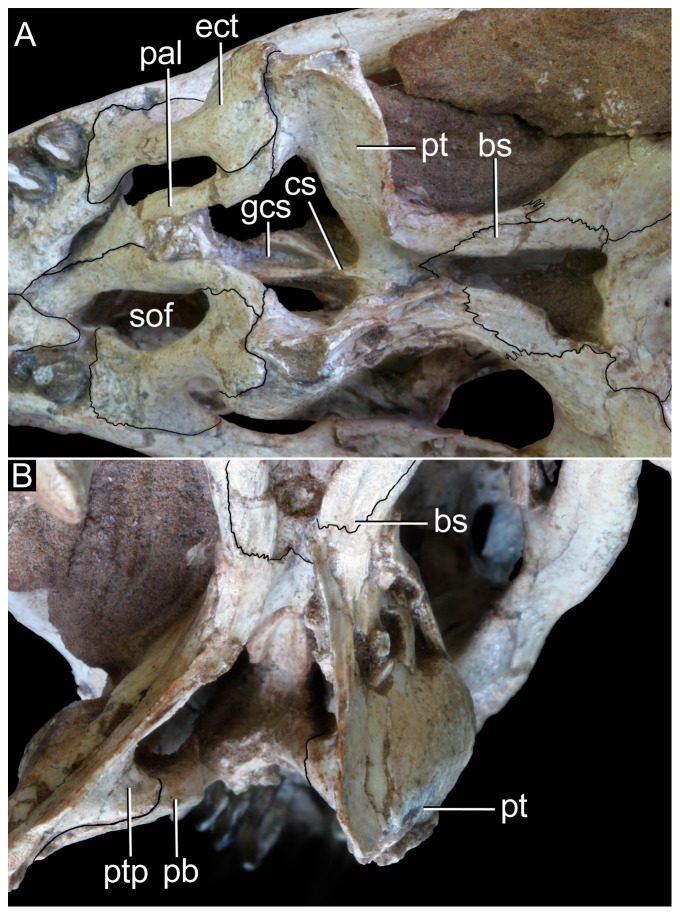
Choanal region of *Caipirasuchus stenognathus* (MZSP-PV 139). **A**, ventral view; **B**, posterior view. Abbreviations: **bs**, basisphenoid; **cs**, choanal septum; **ect**, ectopterygoid; **gcs**, groove on choanal septum; **pal**, palatine; **pb**, palatine bar; **pt**, pterygoid; **ptp**, pterygoid platform for palatine bar; **sof**, suborbital fenestra.

**Figure 22:**
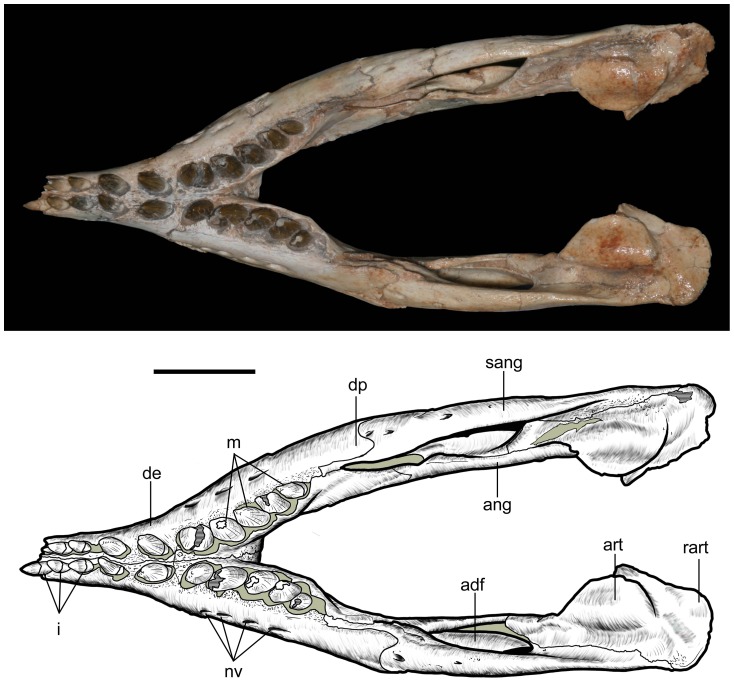
Mandible of *Caipirasuchus stenognathus* (MZSP-PV 139) in dorsal view. Abbreviations: **ang**, angular; **art**, articular; **de**, dentary; **dp**, dorsal process of posterodorsal branch of the dentary; **adf**, aductor mandibular fossa; **i**, incisiviform; **m**, molariform; **nv**, neurovascular foramina; **sang**, surangular; **rart**, retroarticular process. Scale bar equals 2.5 cm.

**Figure 23:**
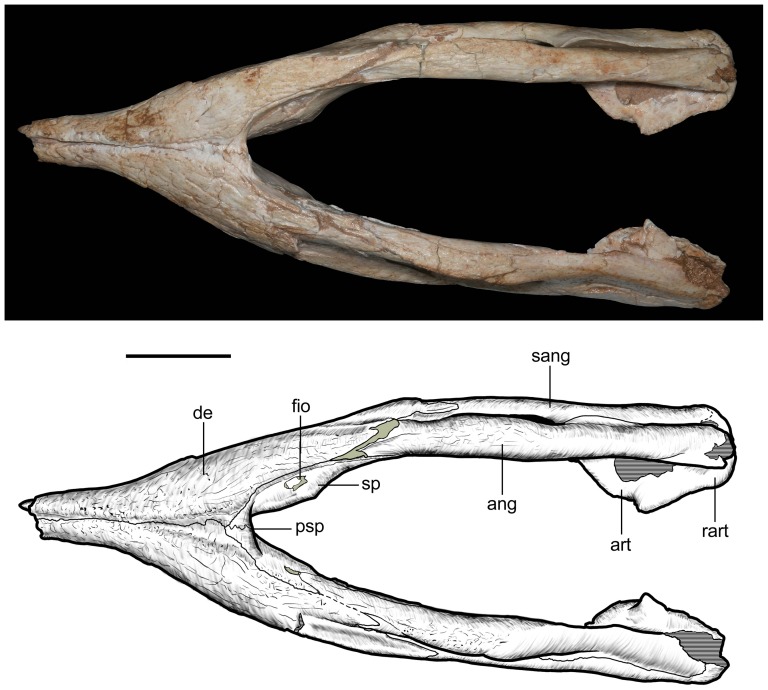
Mandible of *Caipirasuchus stenognathus* (MZSP-PV 139) in ventral view. Abbreviations: **ang**, angular; **art**, articular; **de**, dentary; **fio**, foramen intermandibularis oralis; **psp**, posterior spleniel peg; **sang**, surangular; **sp**, splenial; **rart**, retroarticular process. Scale bar equals 2.5 cm.

**Figure 24:**
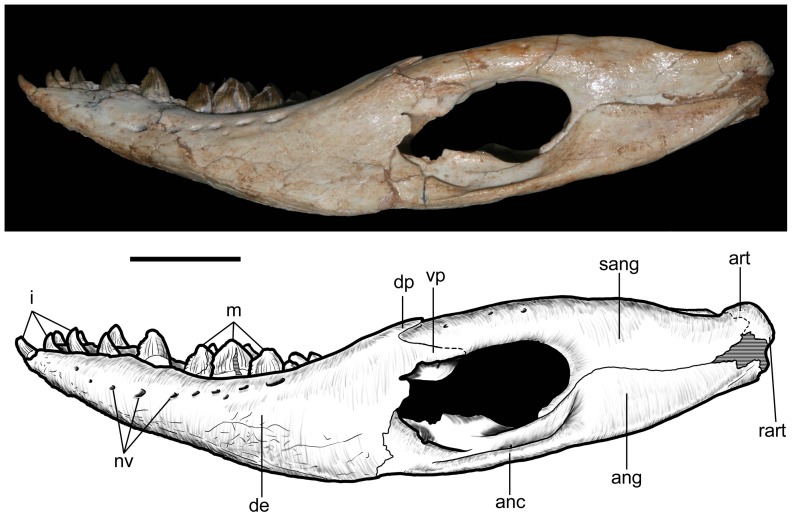
Mandible of *Caipirasuchus stenognathus* (MZSP-PV 139) in left lateral view. Abbreviations: **anc**, angular crest below external mandibular fenestra; **ang**, angular; **art**, articular; **de**, dentary; **dp**, dorsal process of posterodorsal branch of the dentary; **i**, incisiviform; **m**, molariform; **nv**, neurovascular foramina; **sang**, surangular; **rart**, retroarticular process; **vp**, ventral process of posterodorsal branch of the dentary. Scale bar equals 2.5 cm.

**Figure 25:**
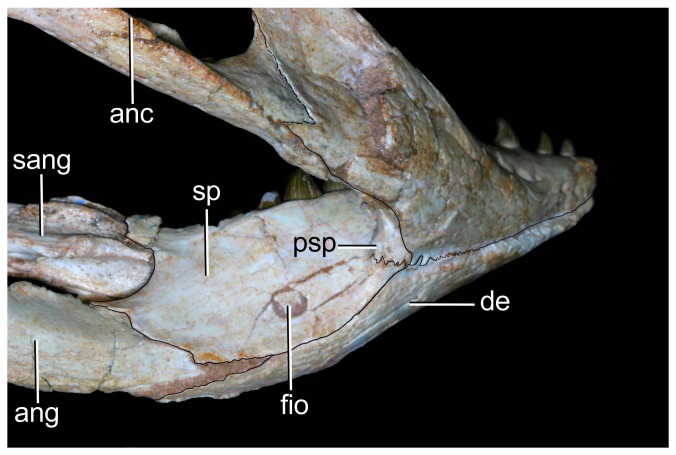
Anterior region of left mandibular ramus of *Caipirasuchus stenognathus* (MZSP-PV 139) in posteroventromedial view. Abbreviations: **anc**, angular crest below external mandibular fenestra; **ang**, angular; **de**, dentary; **fio**, foramen intermandibularis oralis; **sang**, surangular; **sp**, splenial; **psp**, posterior splenial peg.

**Figure 26:**
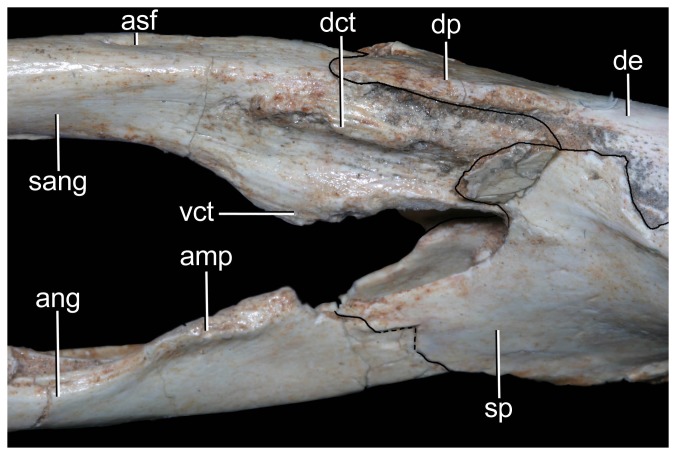
Left mandibular ramus of *Caipirasuchus stenognathus* (MZSP-PV 139) in medial view. Abbreviations: **amp**, ascending medial process of angular; **ang**, angular; **asf**, anterior surangular foramen; **dct**, dorsal coronoid tuberosity of surangular; **de**, dentary; **dp**, dorsal process of posterodorsal branch of the dentary; **sang**, surangular; **sp**, splenial; **vct**, ventral coronoid tuberosity.

**Figure 27:**
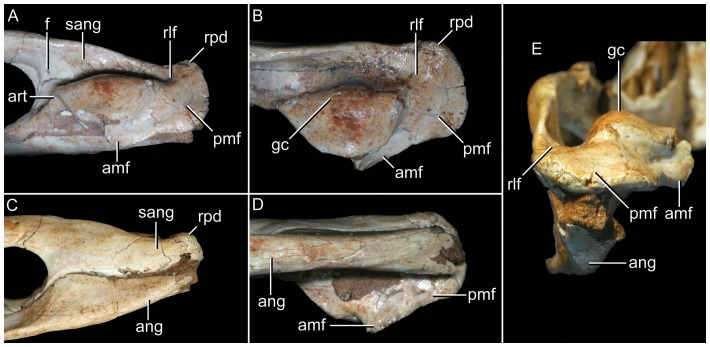
Articular region of left mandiblar ramus of *Caipirasuchus stenognathus* (MZSP-PV 139). **A**, medial; **B**, dorsomedial; **C**, lateral; **D**, ventral; **E**, posterior views. Abbreviations: **amf**, anterior surface of retroarticular medial flange; **ang**, angular; **art**, articular; **f**, foramen; **sang**, surangular; **gc**, glenoid crest; **pmf**, posterior surface of retroarticular medial flange; **rlf**, retroarticular lateral flange; **rpd**, retroarticular posterodrosal process.

**Figure 28:**
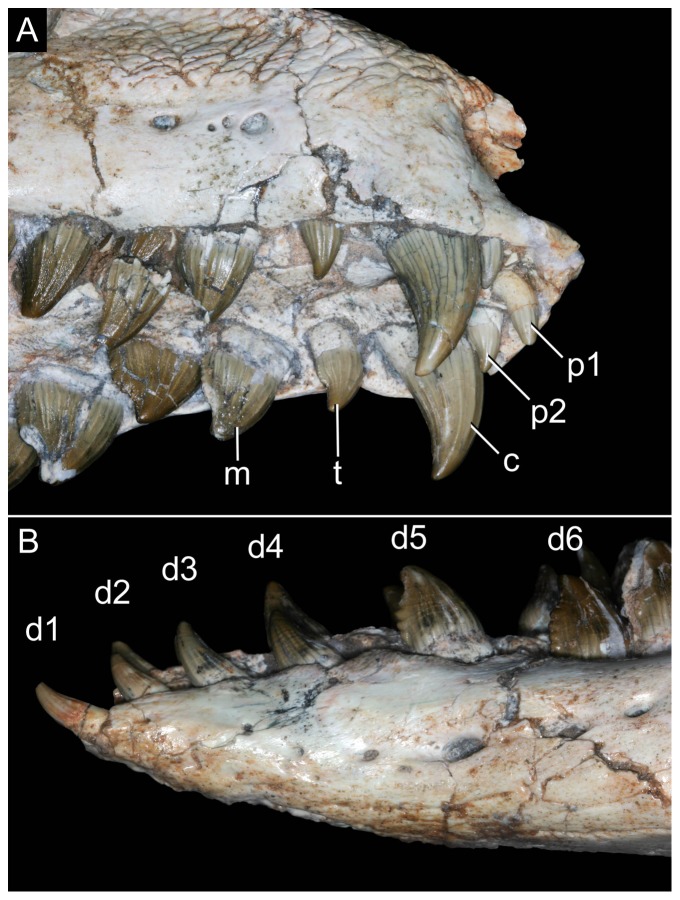
Incisiviform and caniniform teeth of *Caipirasuchus stenognathus* (MZSP-PV 139). **A**, right premaxillary and anterior maxillary teeth in anterolateral view; **B**, anterior region of left toothrow in lateral view. Abbreviations: **c**, caniniform tooth; **d1-6**, dentary teeth; **m**, maxillary molariform; **p1-2**, premaxillary teeth; **t**, transitional tooth.

**Figure 29:**
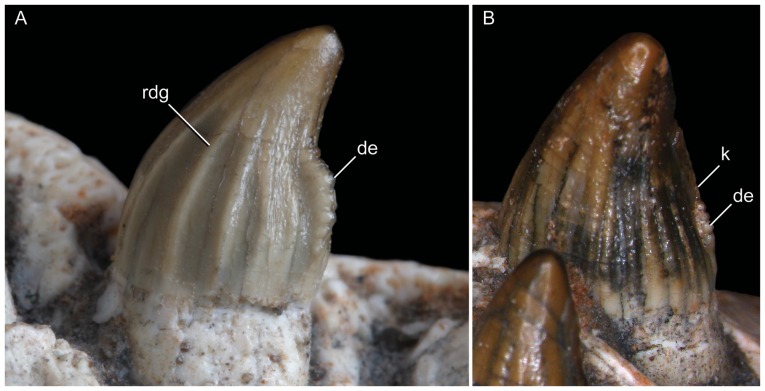
Transitional teeth of *Caipirasuchus stenognathus* (MZSP-PV 139). **A**, fourth premaxillary tooth in posterolingual view; **B**, fourth dentary tooth in posterolingual view. Abbreviations: **k**, keel; **den**, denticle; **rdg**, ridge.

**Figure 30:**
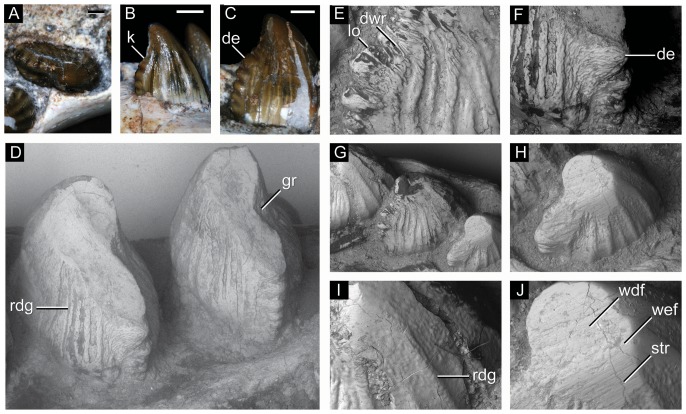
Molariform teeth of *Caipirasuchus stenognathus* (MZSP-PV 139). **A**, third maxillary tooth in occlusal view; **B**, fifth dentary tooth in buccal view (image flipped horizontally to better comparison with C); **C**, sixth dentary tooth in buccal view; **D–J**, SEM images: **D**, two posteriormost maxillary teeth in ventrolingual view; **E**, mesial keel of posterior lower tooth showing denticles and enamel wrinkling; **F**, basal denticles of posterior upper tooth; **G**, posterior lower tooth; **H**, posteriormost lower tooth with wear facet; **I**, detail of enamel surface in broad region of the crown; **J**, wear facet of posteriormost lower tooth. Abbreviations: **den**, denticle; **k**, keel; **lo**, loph; **dwr**, enamel wrinkles on denticles; **gr**, groove (lingual in lower and buccal in upper teeth); **rdg**, ridges; **wdf**, wear facet (dentine); **wef**, wear facet (enamel); **str**, striae. Scale bar equals 0.2 cm.

#### Diagnosis

Sphagesaurid crocodyliform that differs from other species of the genus by the following unique combination of characters (autapomorphies marked with asterisk): maxilla forming part of the orbital margin (absence of lacrimal-jugal contact), maxillo-palatine fenestra, nasal with smooth depressions on their posterior region (flanked by elevated and ornamented ridges located close to the contact with the maxilla and lacrimal)*, lacrimal with pointed posterodorsal process located at anterodorsal corner of orbit that overhangs posterior opening of lacrimal duct*, longitudinally oriented anterior region of prefrontal-frontal suture on dorsal surface of skull, postorbital with smooth and recessed facet for posterior palpebral that extends posteriorly underneath ear-flap groove*, anterolateral corner of the pterygoid flange with elevated platform that projects medially and is overlapped by posterior end of palatine bar*, anteroposterior constriction at the medial origin of pterygoid flanges (lateral to choanal groove), choanal septum of pterygoid tapering posteriorly and bearing marked groove on its ventral surface, external anterior branch of surangular enters into deep U-shaped concavity between ventral and dorsal process of posterodorsal branch of the dentary, distinct anterior process of medial flange of retroarticular process*.

#### Etymology

From the greek “stenos” meaning narrow and “gnathos” meaning jaw, a reference to the narrow anterior portion of the mandible.

#### Holotype

MZSP-PV 139. An almost complete skull, lacking the posterior left region and part of the occipital region, found in articulation with complete lower jaws (Figures 1–30).

#### Occurrence and Geological Setting

Fossil remains of *Caipirasuchus stenognathus* were collected in an outcrop at Boa Esperança farm, Prudêncio de Moraes District of General Salgado City, state of São Paulo. This locality has also produced the remains of *Baurusuchus albertoi* [32]. The specimen was found just above a small scarp (coordinates 20°34′01.44″S, 50°27′49.89″W) on a slope at the right margin of the Ribeirão Buritis (Buritis Creek), a tributary of the river São José dos Dourados, 13 km northwest of General Salgado, in the central-eastern part of the Bauru Basin.

The Bauru Basin [33] is a Late Cretaceous interior cratonic basin developed in the central-southeastern portion of the South American Platform over toleiitic basalts of the Serra Geral Formation and the sedimentary rocks of the Caiuá Basin, both of Early Cretaceous age.

The Bauru Basin has an area of about 370,000 sq. km (Figure 6) and encompasses the sedimentary rocks of the Bauru Group, a continental sandy succession with a maximum preserved thickness of more than 300 m and average around 100 m [34]. The basin was probably formed by slow and gradual subsidence in response to the loading of its substrate by Early Cretaceous volcanic rocks [35].

Many stratigraphic divisions were proposed for the Bauru Group in the São Paulo State (e.g. [34], [36], [37], [38]). In general, are recognized the Adamantina, Araçatuba, Presidente Prudente and Marília formations (Figure 7). The Adamantina Formation comprises massive to stratified sandstones and mudstones and covers the massive to slightly stratified sandstones of the Santo Anastácio Formation, the uppermost unit of the Early Cretaceous Caiuá Basin.

The Araçatuba Formation probably represents a paleoswamp [39] or a paleolake [34]. Deposits of the Adamantina Formation are presently related to an alluvial system comprising lacustrine deltas and fluvial braided and meandering deposits [34], probably related with cycles of flooding events in the General Salgado area [40]. Isotopic δ^18^O data from ostracod carapaces of lacustrine deposits in the Adamantina Formation suggest the predominance of freshwater conditions [41]. The Presidente Prudente Formation is also related to a fluvial meandering system [37], whereas the Marília Formation, at the top of the Bauru Group, includes alluvial fans related to the uplift and erosion of the eastern border of the Bauru Basin.

The Late Cretaceous age of the Bauru Group was established on the basis of its fossil content. The age varies from Turonian to Santonian (ostracods of the Adamantina Formation [41]), Coniacian to Maastrichtian (dinosaur bones of Adamantina and Marília Formations [42]), or Maastrichtian (vertebrate fossils [37]).

The specimen of *Caipirasuchus stenognathus*, comprising an almost complete skull and lower jaw, was found at the lower part of the Adamantina Formation. A composite section around the site of the fossil occurrence revealed the following strata: 1) a lower, 2.5 m thick package of fine-grained massive to slightly stratified sandstone with calcite cement, including calcite nodules, coprolites, and the *C. stenognathus* holotype, which probably represents exposed sand bars in a fluvial braided system; 2) an upper, about 4.5 m thick section of cyclic, fining-upward medium- to fine-grained sandstone in tabular decimetric layers, with cross-stratified coarse-grained sandstone in tabular interbeds up to 20 cm thick, which is probably related to a flood plain with interbedded crevasse deposits in a fluvial meandering system (Figure 8). The position of the fossil occurrence, at the lower part of the Adamantina Formation, led us to consider likely a Coniacian age for the studied stratigraphic section.

#### Comments

Although the skull has some lateromedial compression, this specimen is certainly the best-preserved material of *Caipirasuchus* known so far, with the dentition preserved almost without damage, which allows recognizing clear differences with other sphagesaurid genera and the two other species of *Caipirasuchus*.

The new taxon is diagnosed by several autapomorphies, including three characters that are absent in both *C. paulistanus* and *C. montealtensis*: the smooth depressed surface of the posterior region of the nasals (Figure 2), the narrow and pointed posterodorsal process of the lacrimal that overhangs the opening for the lacrimal duct, and the anterolateral corner of the pterygoid flange bearing a distinct platform that is overlapped by the posterior end of the palatine bar (Figure 4). Two other characters that are so far autapomorphic of this new species are the smooth facet for posterior palpebral on postorbital that extends posteriorly underneath the ear-flap groove and the distinct anterior process of the medial flange of the retroarticular process (see Description). These two characters are absent in other sphagesaurids but cannot be determined in the type specimens of *C. paulistanus* and *C. montealtensis*, and therefore may be autapomorphic of the new taxon or be synapomorphic of this genus.

A noteworthy feature that distinguishes *C. stenognathus* from the two other species is the maxilla forming part of the orbital margin, due to the absence of lacrimal-jugal contact (Figure 1). In *C. paulistanus* and *C. montealtensis* the jugal bears a distinct anterodorsal process that extends anterior to the orbit and broadly contacts the lacrimal, participating from the posteroventral corner of the antorbital fossa (Figure 1). Similarly, the choanal septum of *C. stenognathus* tapers posteriorly and bears a marked groove on its ventral surface, whereas *C. paulistanus* and *C. montealtensis* have a posteriorly broad choanal septum (Figure 4). The septum of *C. montealtensis* is ventrally convex (clearly lacking the groove) but this condition cannot be determined with certainty in *C. paulistanus* as the ventral surface of the choanal septum is damaged in the holotype.

Other difference that distinguishes *C. stenognathus* from both *C. paulistanus* and *C. montealtensis* is the longitudinally oriented anterior region of the prefrontal-frontal suture on the dorsal surface of the skull, which gives a tabular shaped anterior end to the frontal of *C. stenognathus* (Figure 2); resembling the condition of *Yacarerani* and *Mariliasuchus*. This suture is obliquely oriented in *C. paulistanus* and *C. montealtensis* so that the lateral margins of the frontal are set obliquely to each other (Figure 2), converging anteriorly (as in *Notosuchus* and most notosuchians). Furthermore, *C. stenognathus* differs from *C. montealtensis* in the absence of a deep parachoanal fossa (Figure 4) and the presence of a longitudinally oriented lacrimal-prefrontal suture (Figure 2), and from *C. paulistanus* in the presence of an anteroposterior constriction at the medial origin of pterygoid flanges (Figure 4).

## Description

### General features

The specimen MZSP-PV 139 has preserved the skull, mandibles, and the hyoids in natural position. The skull and mandibles are slightly crushed obliquely, from the left dorsolateral side to the right ventrolateral side. The upper left temporal region has not been preserved, including the left quadrate and occipital region.

The skull is oreinirostral (*sensu* [43]) with external nares facing anteriorly, a plesiomorphic feature of crocodyliforms interpreted as characteristic of terrestrial forms. The rostrum broadens gradually towards the orbit and seems to have a slight lateral expansion at the level of the large premaxillary caniniform (Figure 9). In lateral view, the skull tapers gradually anteriorly (Figures 10–11). The palpebrals have not been preserved but a large facet on the dorsal surface of the prefrontal-lacrimal suture denotes the presence of a large anterior palpebral and a small facet on the anterolateral region of the dorsal surface of the postorbital indicates the presence of a posterior palpebral. These features are consistent with the condition of most notosuchians that have a large anterior palpebral and a smaller posterior palpebral (e.g., *Notosuchus*, *A. gomesii*, baurusuchids).

The antorbital fenestra [44] is small and seems to be subcircular in shape although the crushing of the specimen precludes the determination of its precise shape (Figures 10, 11). Its ventral margin, however, is similar on both sides, being rounded and located at the level of the dorsoventral midpoint of the orbit. This condition resembles that of other notosuchians (e.g., *Notosuchus*) and *C. montealtensis* but differs from the ventral margin of the antorbital fenestra of *C. paulistanus* that is posteroventrally elongated and reaches the lower third of the dorsoventral height of the orbit (Figure 1). The orbits are large, rounded, and laterodorsally exposed (although if the deformation is corrected, the orbits mostly face laterally). Based on the preserved elements, the skull roof seems to be relatively narrow and elongated, bearing anteroposteriorly elongated and ovoid supratemporal fossae (*sensu* [30]), with a similar shaped fenestra displaced anteriorly within the fossa (Figure 9). The infratemporal fenestrae are triangular and face only dorsolaterally. Although none of them have been completely preserved, the infratemporal fenestrae seem to be anteroposteriorly longer than dorsoventrally high. The suborbital fenestrae are ovoid shaped, lateromedially narrow and anteroposteriorly long, with acute anterior ends. The external mandibular fenestra is large and subcircular with its anteroposterior axis slightly longer than its dorsoventral depth. The opening is remarkably large, occupying approximately 75% the dorosoventral height of the mandibular ramus. The dorsal surface of the skull table and rostrum, as well as the ventral surface of the mandibular symphysis, is ornamented by well-developed thin grooves that are separated by broad but poorly elevated ridges. The frontal bears a long sagittal crest as in the other species of *Caipirasuchus* and some notosuchians (e.g., *Notosuchus*). The mandiblar rami are straight and converge anteriorly to form an elongated symphyseal region with parallel sides oriented parasagitally as in all sphagesaurids [5], [10]–[12]. Cranial measurements are given in Table 2.

**Table 2:** Skull and mandibular measurements of *Caipirasuchus stenognathus* MZSP-PV 139 (in millimeters).

**Skull**	
Skull length (from the tip of the snout to the end of squamosal posterolateral process)	160.93
Basal skull length (from tip of snout to occipital condyle)	140.19
Rostrum length (from tip of the snout to anterior end of orbit)	63.76
Maximum skull width (across jugals)	60.29
Maximum frontal width	31.63
Minimum frontal width	11.01
Orbital anteroposterior length	34.13
Temporal height (from quadrate condyle to skull roof)	49.79
Maximum length of supratemporal fossa	33.29
Maximum length of supratemporal fenestra	20.92
**Mandible**	
Mandibular length	R 160.97 L 158.8
Maximum symphyseal length	53.68
Dentary length	R 97.79 L 99.1
Anteroposterior length of mandibular fenestra	39.13
Maximum height of mandibular ramus (measured at the mandibular fenestra)	R 36.13 L 32.47

#### Skull

The premaxilla forms the ventral and lateral edges of the external nares but does not participate on the dorsal margin of this opening. The anteroventral region of the premaxilla, below the external nares, has a small process that projects anterodorsally along medial contact of both premaxillae (Figure 9). A similar crest is found in most basal crocodyliforms that lack a complete osseous internarial bar, including most notosuchians (e.g., *Araripesuchus gomesii*, *Notosuchus*, *Mariliasuchus amarali*, *Adamantinasuchus*, *Yacarerani*). The premaxilla has only a shallow and poorly defined perinarial depression that extends along the lateral and ventral borders of the nares. In *C. stenognathus* (and most other sphagesaurids), however, the perinarial depression is not the distinct and anteriorly facing fossa of some notosuchians (e.g., *Simosuchus*, *Notosuchus*, *Mariliasuchus*). Posterior to the external nares the premaxilla is well exposed on the lateral surface of the rostrum, forming approximately 30% of the rostral length. The external surface of the premaxilla is laterally bulged posterior to the external nares, due to the presence of a large caniniform tooth. Along this region the premaxilla is divided in two different planes of exposure. A ventral region that is vertically oriented and smooth and a dorsal region that is dorsolaterally exposed and ornamented with thin grooves and ridges. The smooth ventral surface bears a relatively large neurovascular foramen that opens anteriorly and is located just rostrally to the suture with the maxilla (Figure 10), resembling the condition of *Araripesuchus* [24]. This foramen is poorly preserved on the right side of *C. stenognathus* (Figure 11) and has been artificially enlarged because of the oblique crushing of the specimen. The premaxilla-maxilla suture on the external surface of the rostrum runs posterodorsally along most of its length and then deflects posteriorly bordering a relatively short posterodorsal process of the premaxilla that wedges between the maxilla and the nasal (Figures 9, 10). A relatively short posterodorsal process of the premaxilla is also present in *Caipirasuchus montealtensis*, and *Adamantinasuchus navae*, but not in *Caipirasuchus paulistanus*, *Notosuchus*, and *Comahuesuchus*. The premaxilla-nasal suture runs posteriorly slightly diverging posteriorly and lacks the marked lateral concavity present in *Notosuchus*, *Morrinhosuchus*, and *Mariliasuchus*.

At the alveolar edge, the premaxilla-maxilla suture is located at the level of the fourth premaxillary teeth. On the palatal surface, this suture extends posteromedially from this tooth and then turns and is directed anteromedially. The sinuous premaxilla-maxilla suture on the palate creates a relatively large posterior extension of the palatal ramus of the premaxilla, which lodges a large part of the fourth upper alveolus. This morphology is similarly found in other advanced notosuchians but not in other notosuchians (e.g., uruguaysuchids, peirosaurids, baurusuchids, sebecids). The premaxilla-maxilla suture is interrupted by a foramen incisivum, the margins of which have been damaged due to the crushing of the specimen. Nonetheless, the anterolateral margin of the left side is well preserved and shows this opening was of moderate size (Figure 12) as most notosuchians, except for some sebecids in which this opening is usually absent. The palatal branch of the left premaxilla bears 10 neurovascular foramina along the medial edges of the premaxillary alveoli. Each premaxilla bears four teeth, the third being a large caniniform (see Dentition below).

The maxilla is tabular shaped and is longer than high with a straight buccal edge. As the premaxilla, the external surface of the maxilla is divided in two different regions. The ventral third of the maxilla is vertically oriented and its surface is smooth (above the alveolar edge). The dorsal two-thirds of the maxilla, instead, face dorsolaterally and are ornamented by grooves and ridges. The ventral smooth surface is pierced by numerous neurovascular foramina that are dorsoventrally aligned as in advanced notosuchians (e.g., *Notosuchus*, *Mariliasuchus*, *Yacarerani*, *S. huenei*, *C. montealtensis*). As noted by Turner & Sertich [18] for *S. huenei*, *C. montealtensis*, and *Adamantinasuchus* these foramina are not evenly distributed along the anteroposterior axis in *C. stenognathus*, and there is a gap between the anterior and posterior maxillary foramina. This feature is also present in *Yacarerani* and *C. paulistanus* and may characterize all sphagesaurids (although is unknown in the type material of *Armadillosuchus* and *Caryonosuchus*). There are eleven foramina on the left side but only six on the right side (Figures 10, 11), being the cluster of posterior foramina composed of three or four foramina, the posteriormost of which is the largest and located just anteriorly to the maxilla-jugal contact. All these foramina are openings of ducts that direct dorsomedially into the maxilla that can be observed in CT images of the snout (Figure 13D) and are interconnected by a neurovascular duct that runs longitudinally along the maxilla, located laterally to the alveoli at the level of the limit between the smooth ventral surface of the maxilla and the dorsal ornamented region of this bone (Figure 13E–F). Extant crocodylians have a similar duct through which passes the sensory maxillary nerve (V_2_, anterior branch of the trigeminal nerve; see [45] and references therein). This duct ends posteriorly in a relatively large sinus (Figure 13G) located posterior to the last maxillary tooth, at the level of the maxillary jugal suture and is likely connected to the entrance of the V_2_ nerve located in the orbital cavity (see below).

The anterior half of the dorsal region of the maxilla, as noted above, is ornamented and extends dorsally up to the maxilla-nasal suture. This region of the maxilla is a lateromedially thin lamina that contrasts with the lateromedially broad ventral region that lodges the roots of the maxillary teeth (Figure 13C). The maxilla-nasal suture runs anteroposteriorly, with a slight lateral deflection towards its posterior end. The maxilla-nasal suture ends at the triple contact between these two bones and the lacrimal (Figure 14). This contact precludes the maxilla-prefrontal contact on the posterolateral region of the dorsal surface of the rostrum, as in most mesoeucrocodylians. The triple contact between the maxilla, nasal, and lacrimal is located at the anterior tip of the triangular shaped facet for the anterior palpebral. The maxilla contributes to a minor part of the anterior tip of this facet (Figure 14). Posteriorly to this point, the dorsal edge of the maxilla is sutured to the lacrimal, along a contact that runs ventrally to the articular facet for the palpebral. Ventral to the triple suture (maxilla-nasal-lacrimal) the maxilla has a small and sharp lateral projection that is the anterior limit of the facet for the anterior palpebral. This maxilla-lacrimal suture extends posteriorly up to the dorsal margin of the antorbital fenestra. The maxilla forms at least half of the dorsal margin (Figure 11) and the entire anterior and ventral edges of this opening (Figure 10). The anterior and ventral margins of the antorbital fenestra are well preserved on the left side of *C. stenognathus* and evenly curved, indicating the fenestra was a small circular opening (as in *C. montealtensis* but in contrast to the posteroventrally elongated fenestra of *C. paulistanus*). The maxilla contacts again the lacrimal at the posteroventral corner of the antorbital fenestra along a posteroventrally directed suture that extends from this opening to the anterior margin of the orbit (Figure 10). The maxilla, therefore, limits the ventral projection of the lacrimal, forms part of the anteroventral margin of the orbit, and precludes a contact of the jugal with the lacrimal. This suite of characters resembles the condition of *Mariliasuchus*, *Adamantinasuchus*, and some specimens of *Comahuesuchus* (MACN-N 31) but differs from the condition found in other sphagesaurids (*C. montealtensis*, *C. paulistanus*, *Armadillosuchus*, *Yacarerani*, and *S. huenei*), as wel as other notosuchians (e.g., *Notosuchus*, *Simosuchus*, *Pakasuchus*, *Candidodon*, baurusuchids). The ornamented pattern of the dorsal region of the maxilla is absent in the antorbital region, which surrounds the antorbital opening. This area is smooth and is interpreted as a shallow and extensive antorbital fossa (Figures 10, 11), with poorly delimited margins that includes the entire maxilla-lacrimal contact. A similar type of fossa is found in other sphagesaurids that either have (*C. montealtensis*, C. *paulistanus*) or lack an antorbital fenestra (*S. huenei*, *Armadillosuchus*, *Yacarerani*), as well as in some baurusuchids (*B. pachecoi*). This morphology, however, differs from the well delimited, smaller, and deeper antorbital fossa of most notosuchians (e.g., *Notosuchus*, *Morrinhosuchus*, *Simosuchus*, *Araripesuchus*, peirosaurids, mahajangasuchids), which is the plesiomorphic condition for basal mesoeucrocodylians.

The maxilla is laterally overlapped by the anterior V-shaped process of the jugal, having the tip of the jugal located at the limit between the antorbital depression and the smooth ventral region of the maxilla (Figures 10, 11). The dorsal half of the maxilla-jugal suture extends posteriorly towards the orbital margin, bounding the ventral limit of the antorbital fossa, differing from the condition of both *C. montealtensis* and *C. paulistanus* (see below). The ventral half of the maxilla-jugal suture, instead, is posteroventrally directed delimiting an acute and elongated posteroventral process of the maxilla that reaches the level of the anteroposterior midpoint of the orbit. Such an elongated process is present in most notosuchians (including *C. montealtensis* [10] and *C. paulistanus* [11]) but not in *S. huenei* and in baurusuchids, in which a much shorter and blunter posteroventral process of the maxilla ends anteriorly to the rostral margin of the orbit due to the anteroventral expansion of the jugal. The posterior tip of the posteroventral process of the maxilla wedges between the jugal (dorsally) and the anterior branch of the ectopterygoid (ventrally).

The palatal branch of the left maxilla is well preserved and exposed in ventral view, as the crushing of the specimen displaced the right palatal branch dorsally to its left counterpart (Figures 12, 13F). The palatal branches are dorsoventrally thick and lateromedially narrow, so that the left and right toothrows are not as separated from each other as in *S. huenei* or other notosuchians (e.g., *Notosuchus*, *Mariliasuchus*, *Comahuesuchus*). The condition of MZSP-PV 139, however, is similar to the closely positioned upper toothrows of *C. montealtensis*, *C. paulistanus*, and *Yacarerani*. The ventral surface of the left palatal branch is slightly convex towards the midline, a feature that is more developed on its posterior half. The alveolar margin of the palatal branch bears numerous foramina and slightly developed rugosities and striations (Figure 12). These rugosities are absent in most notosuchians (e.g., *Araripesuchus*, *Notosuchus*, *Mariliasuchus*, *S. huenei*), except for *C. montealtensis*, *Comahuesuchus*, and baurusuchids (*B. pachecoi*, *Stratiotosuchus*). The rugosities of *B. pachecoi* and *Stratiotosuchus*, however, are much more developed than in the other taxa. The posteromedial region of the maxillary palatal branches receives the anterior process of the palatines. The palatines of *C. stenognathus*, however, are slightly displaced from their natural anterior contact with the maxilla (Figure 12). Nonetheless, three significant features can be noted on this region. First, the left maxilla clearly shows that this bone formed most of the anterolateral margin of the suborbital fenestra. This is the plesiomorphic condition for notosuchians (e.g., *Araripesuchus*, *Notosuchus*, *S. huenei*, *C. montealtensis*, *C. paulistanus*) but differs from the condition of *Mariliasuchus* [17] or *Yacarerani* [15] that have the maxilla almost or completely excluded from the margin of the suborbital fenestra, respectively. Second, the maxilla-palatine suture seems to extend anteromedially from the suborbital fenestra with the palatines wedging between the palatal branches of the maxilla. Third, the left maxilla has preserved the natural lateral margins of a maxillo-palatine fenestra, a derived feature otherwise only present in *Mariliasuchus* and *Notosuchus* [46], but differing from the condition of other sphagesaurids, including *C. paulistanus*, *C. montealtensis*, *S. huenei*, and *Yacarerani*. The posterior edge of the palatal branch of the maxilla contacts the ectopterygoid along a transversely oriented suture that forms a small part of the posterior alveolar margin of the last maxillary alveolus (Figure 12), as in *C. paulistanus*, *Mariliasuchus*, and *Yacarerani*. There are six maxillary teeth on each maxilla, which are described in detail below (see Dentition).

The maxilla of *C. stenognathus* extends from the suborbital opening, forming a posteriorly facing wall of the anteroventral corner of the orbital cavity. This vertical maxillary wall restricts the opening of the nasal cavity into the orbit and here we refer to it as the maxillary orbital lamina (Figure 15). The presence of this lamina is a derived feature described as autapomorphic of *S. huenei* [5] given its absence in other notosuchians (e.g., *Araripesuchus*, *Simosuchus*, *Notosuchus*). However, the morphology of *Mariliasuchus* (MZSP-PV 51, UFRJ 105R, URC R68) and *C. paulistanus* resemble the condition of *C. stenognathus* and *S. huenei*. The condition of other sphagesaurids (e.g., *C. montealtensis*, *Yacarerani*, *Adamantinasuchus*) is currently unknown, but the available evidence indicates this feature as a putative synapomorphy of *Mariliasuchus* and sphagesaurids. The ventral margin of the maxillary orbital lamina is sutured to the anteromedial process of the ectopterygoid. The maxilla-ecopterygoid suture encloses a relatively large foramen that likely represents the entrance of the maxillary nerve (V_2_). This foramen is completely enclosed in the maxillary orbital lamina in *S. huenei* and *Mariliasuchus* (URC-R68). The ventral region of the lateral margin of the maxillary orbital lamina is sutured to the medial surface of the jugal. Dorsally to this point the maxillary orbital lamina meets the lateral surface of the maxilla at a right angle forming the orbital margin (Figure 15). The dorsal third of the lateral margin of the maxillary orbital wall is sutured to the descending process of the lacrimal. The suture with the lacrimal runs along a ridge that forms the anterior edge of the orbit. The dorsal edge of the maxillary orbital lamina is sutured to the lateral region of the descending process of the prefrontal (Figure 15), as in *S. huenei* [5].

The nasals cover the entire dorsal surface of the rostrum and their anterior end overhang the narial opening forming an anteriorly tapering pointed process. Although the tip of this process is damaged, it does not seem to be as anteriorly projected as that of *A. gomesii*. The nasals gradually broaden posteriorly along their contact with the maxilla, up to the triple contact of these bones and the lacrimal and their lateral edges converge from this point along their suture with the prefrontals and frontal (Figure 9). The anterior half of the nasal is ornamented with thin grooves, most of which are anteroposteriorly aligned. The posterior half of each nasal, instead, bears a smooth and depressed central area that is flanked by two slightly elevated and ornamented areas (Figure 9). One of them is a small protuberance located just anteriorly to the nasal-frontal contact. The other elevated area is an elongated and lateromedially broad ridge that extends from the anterior tip of the palpebral facet to the anteroposterior midpoint of the nasal-nasal suture (Figures 9, 14). The ornamentation in this region is formed by narrow subparallel grooves that run in a posterolateral-anteromedial direction. In lateral view, the dorsal surface of the nasals is slightly concave posteriorly (at the level of the smooth depressions) and slightly convex along the ornamented anterior half (Figures 10, 11). The presence of a smooth depression flanked by these elevated and ornamented areas on each nasal seems to be an autapomorphic character of *Caipirasuchus stenognathus*, as these features are absent in *C. paulistanus* and *C. montealtensis* (and other sphagesaurids such as *Yacarerani* and *Armadillosuchus*), although this region is currently unknown in some members of this clade (e.g., *S. huenei*, *Caryonosuchus*, *Adamantinasuchus*).

The anterior fourth of the nasals (along their contact with the premaxilla) is dorsoventrally thick and the CT data does not show any signs of cavities or ducts (Figure 13B). However, more posteriorly, each nasal houses a longitudinal duct that bifurcates posteriorly into two ducts (a medial and a lateral duct) at the level of the first maxillary tooth (Figure 13C). The medial duct opens into the nasal cavity at the level of the second maxillary tooth, whereas the lateral duct becomes larger posteriorly and directs laterally running close to the nasal-maxilla contact. At the level of the third maxillary tooth this duct seems to open into the nasal cavity, although the low resolution of the CT data precludes determining this with certainty. Posterior, at the level of the smooth depressed area, the nasals lack any signs of duct passing along them and decrease in thickness (Figure 13D). At the moment it is not clear if the longitudinal ducts located on the anterior region of the nasals were in fact connected in some way to the lacrimal duct that is located much more posteriorly on the lacrimal (see below).

Posterior to its broadest point, at the anterior tip of the palpebral facet, the lateral edge of the nasal is sutured to the medial edge of the dorsal surface of the lacrimal, and directs posteromedially along the border of the palpebral facet (Figure 14). More posteriorly, the nasal-prefrontal suture continues posteromedially for approximately 4 mm on the dorsal surface of the snout to meet the frontal. The posterior edge of the nasals forms an interdigitated suture with the anterior process of the frontal (Figure 9). The morphology of the posterior region of the nasal closely resembles that of advanced notosuchians (e.g., *Notosuchus*, *Mariliasuchus*, *Yacarerani*, *C. montealtensis*, *C. paulistanus*) and *Comahuesuchus* (MUC-PV 202). In particular it shares with these taxa the broad lateral expansion at the anterior tip of the palpebral facet, the posteromedially directed suture with the lacrimal and prefrontal, and the posteriorly concave interdigitated suture with the frontal. The first of these features is also present in baurusuchids (*B. salgadoensis*, *Stratiotosuchus*) although in this group the nasal extends further, onto the lateral surface of the antorbital region of the rostrum. More basal notosuchians, instead, have much narrower nasals, the lateral margins of which are far from the palpebral facet (e.g., *A. gomesii*, *A. patagonicus*, *A. wegeneri*, *Simosuchus*, *Malawisuchus*, *Karprosuchus*, peirosaurids).

The lacrimal of *C. stenognathus* has a broad dorsal process that is exposed on the posterolateral region of the dorsal surface of the rostrum (Figures 9, 14) and an anteroposteriorly narrow and short descending process (Figures 10, 11). The descending process can be divided into a ventral half and a dorsal half, the former a narrow lamina of bone that faces laterally and the latter a broader surface that faces posterolaterally within the orbit.

The ventral half of the descending process is vertically directed and extends between the antorbital fenestra and the orbital margin. Only the posterior margin of the antorbital opening has been preserved on the left lacrimal (Figure 10), but the slightly crushed right lacrimal shows that its contribution to the dorsal margin of this opening was very short (Figure 11). The ventral end of the descending process of the lacrimal is sutured to the maxilla along a posteroventrally directed and straight suture (Figure 10) and does not broaden anteroposteriorly with respect to the rest of the descending process, as it does in *C. montealtensis*. The posteroventral end of the lacrimal extends ventrally as a narrow tapering process that fails to contact the jugal, resembling the condition of *Adamantinasuchus* and some specimens of *Comahuesuchus*. Most other notosuchians, including *C. paulistanus* and *C. montealtensis*, have a ventral process of the lacrimal that is extensively sutured to the anterodorsal corner of the jugal (e.g., *Araripesuchus*, *Notosuchus*, *S. huenei*, *Armadillosuchus*, *Yacarerani*). The condition of *Mariliasuchus* is intermediate, as only the acute tip of the posteroventral pointed process of the lacrimal contacts the jugal [17]. Along this region, the lacrimal does not enter into the orbit and is sutured to the posterior wall of the maxilla (see above) along a vertical ridge that forms the central portion of the anterior margin of the orbit (Figure 15).

The dorsal half of the descending process of the lacrimal extends into the anterior wall of the orbit and contacts the prefrontal. The dorsolateral corner of this surface bears the large posterior opening of the lacrimal duct. In contrast to the complex morphology of this opening in *S. huenei* [5], in *C. stenognathus* the lacrimal foramen is subcircular and mostly enclosed within the lacrimal (Figures 13G, 15). The CT slices show that this duct quickly opens anteriorly on the medial surface of the lacrimal. A pointed posterodorsal process located at the anterodorsal corner of the orbit overhangs the posterior opening of the lacrimal duct, a process that seems to be absent in *C. paulistanus*. The lacrimal has a longitudinal ridge that extends anteriorly from this process on its lateral surface, delimiting the descending process from the dorsal process of this bone (Figures 10, 15). The ridge separating the dorsal from the descending process is present in *C. paulistanus* and *C. montealtensis* but is located dorsally to the posterior opening of the lacrimal duct rather than at the level of this opening as in *C. stenognathus*.

In dorsal view, the dorsal process of the lacrimal is a triangular shaped surface that is obliquely oriented with respect to the longitudinal axis of the skull (Figure 14). This surface is limited by the longitudinal ridge described above (laterally), the suture with the nasal (anteromedially), and the suture with the prefrontal (posteromedially). Most of this surface is smooth and concave and forms anterolateral half of the anterior palpebral facet. A small foramen is located close to the lateral edge of the dorsal surface of the lacrimal (Figure 14A), which has not been preserved on the right side. The anteromedial edge of dorsal process of the lacrimal is sutured to the nasal, forming a slightly elevated and rugose margin of the palpebral facet (Figure 14). Posteriorly, this suture is placed medially to the palpebral facet and reaches the point of triple contact between these two bones and the prefrontal. At this area, the medialmost region of the lacrimal is ornamented and forms part of the dorsal surface of the rostrum (Figures 9, 14). The lacrimal-prefrontal suture extends posterolaterally from this point towards the pointed posterodorsal process of the lacrimal. In *C. paulistanus* this area is not visible given the anterior palpebral is in its natural position but in *C. montealtensis* the lacrimal-prefrontal suture runs posteriorly rather than posterolaterally so that the posterior end of the lacrimal contribution to the articular facet of the anterior palpebral is lateromedially broader than in *C. stenognathus*. This suture is sigmoid shaped and passes through the palpebral articular facet (Figure 14). Determining the precise sutural contacts in this area is difficult and has been the source of alternative interpretations in most notosuchian taxa due to: poor preservation (e.g., *A. patagonicus* [20], [47]), presence of anterior palpebral (e.g., *Stratiotosuchus*, *C. paulistanus*), or presence of an extensively ornamented surface (e.g., *Notosuchus*). However, MZSP-PV 139 shares with advanced notosuchians (*Mariliasuchus*, *Notosuchus*, *Yacarerani*) and *Comahuesuchus* the medial ornamented projection of the lacrimal onto the dorsal surface of the rostrum, which borders most of the anterior margin of the prefrontal and partly limits its anterior projection.

The prefrontal has a dorsal process exposed on posterolateral region of the rostrum and a descending process that extends on the anterodorsal corner of the orbit. The dorsal exposure of the prefrontal is an anteroposteriorly short and lateromedially broad triangle that is limited laterally by the sigmoid suture with the lacrimal, medially by the sutures with the nasal and frontal, and posteriorly by the anterodorsal corner of the orbit. A short and broad dorsal exposure of the prefrontal is also found in other sphagesaurids (*C. paulistanus*, *C. montealtensis*, *Adamantinasuchus*, *Yacarerani*), other advanced notosuchians (e.g., *Notosuchus*, *Mariliasuchus*), and *Comahuesuchus*, but contrasts with the narrow and elongated condition found in baurusuchids, sebecids, basal notosuchians (e.g., *A. gomesii*, *Simosuchus*, *Candidodon*, *Malawisuchus*, *Kaprosuchus*, peirosaurids), and other crocodyliforms.

The dorsal surface of the prefrontal is divided in two distinct regions. The lateral region is triangular shaped and forms the posteromedial half of the smooth facet for the anterior palpebral. The medial region of the dorsal surface of the prefrontal is rectangular shaped and ornamented with obliquely oriented grooves and ridges (Figure 14). The suture with the nasal is extremely short and runs for approximately 3 mm along the anteromedial edge of the dorsal surface of the prefrontal. The suture with the frontal is longitudinally along its anterior half and curves posterolaterally along its posterior half (Figures 9, 14B). In both *C. paulistanus* and *C. montealtensis* the prefrontal-frontal suture directs posterolaterally along its entire length, being evenly curved in the former and rather straight in the latter. The posterior margin of the dorsal surface of the prefrontal is posteriorly concave and extends from the tip of the posterodorsal process of the lacrimal to the point where the prefrontal-frontal suture meets the orbital margin (Figures 9, 14).

The descending process of the prefrontal extends ventrally from the orbital margin, forming a right angle with the dorsal surface of this bone. The prefrontal descending process is a thin lamina that faces posterolaterally and slightly ventrally, along the anterodorsal corner of the orbit (Figure 15). The oblique orientation of this process resembles the condition of other notosuchians (e.g., *Araripesuchus*, *Simosuchus*, *Notosuchus*, *Comahuesuchus*, *Mariliasuchus*, peirosaurids, sebecids, baurusuchids) and basal crocodyliforms (e.g., *Gobiosuchus*) but contrasts with the longitudinally broad lamina of some extant crocodylians. The descending lamina of the prefrontal extends posteriorly along the posteroventrally directed suture with the frontal within the orbital cavity. The posterior end of the descending process of the prefrontal is located at the anteroposterior midpoint of the orbit, where the prefrontal-frontal suture reaches the lateral margins of the *crista cranii*. The prefrontal pillars have not been preserved but the left prefrontal has preserved the base of this process, located between the posterior projection described above and the anterolateral region of the descending process of the prefrontal that meets the lacrimal and maxilla (Figure 15). The anterolateral margin is sutured to the lacrimal. This suture runs from the corner of the posterolateral process of the lacrimal to the point of triple contact between these two bones and the maxillary orbital wall. The prefrontal-lacrimal suture passes along the broad depression that surrounds the posterior opening of the lacrimal duct, as in most crocodyliforms.

The frontal is almost completely preserved except for the posterolateral region of the left side that has been damaged (Figures 9, 16). The frontal is unpaired as in all mesoeucrocodylians and has a long sagittal crest that extends along a large part of the dorsal surface of the frontal, being absent along the anterior fourth of its length. This resembles the condition of *C. montealtensis* but contrasts with the crest of *C. paulistanus* that reaches the anterior end of the frontal. The crest is narrow and sharp at its central region, whereas its anterior end is low and moderately broad and its posterior end is high and markedly broad (Figure 16). A similar crest is present in some notosuchians (e.g., *Notosuchus*, *Comahuesuchus*, *C. montealtensis*, *C. paulistanus*, *Simosuchus*, *Anatosuchus*, *Araripesuchus wegeneri*, sebecids, baurusuchids, *Kaprosuchus*) but is absent in others (e.g., *Mariliasuchus*, *Yacarerani*, *Armadillosuchus*). This crest is slightly more developed than in the above-mentioned taxa, but certainly not as much as in *Iberosuchus* and some sebecids [48]. The dorsal surface of the frontal is irregularly ornamented with grooves that are less developed than those of the dorsal surface of the nasal and maxilla (Figure 16).

The frontal has an anterior process with parallel edges that extends between the prefrontals and forms the anterior fourth of this element. This morphology of the anterior region of the frontal resembles the condition of some advanced notosuchians (*Mariliasuchus*, *Armadillosuchus*, and *Yacarerani*), but differs from the frontal of others (*Notosuchus*, *C. montealtensis*, and *C. paulistanus*) that have the frontal lateral margins converging anteriorly along the suture with the prefrontal. The rostral edge of the anterior process is convex and projects between the nasals and bears a small and narrow pointed process at its tip (Figure 16). Posteriorly the frontal broadens gradually along its suture with the prefrontals and reaches orbital margin, where the lateral margins of the frontal extend posteriorly and gradually deflect laterally to meet the postorbital. The orbital margin is not raised with respect to the dorsal surface of the frontal and is ornamented with slightly developed rugosities. This ornamented edge, however, does not seem to be a sutural contact for the anterior palpebral.

The frontal-postorbital suture runs posteromedially from the posterodorsal margin of the orbit towards the anterior edge of the elongated supratemporal fossa (Figure 16). The dorsal surface of the frontal is raised along this suture forming a slightly elevated and obliquely oriented crest, but is not as strongly developed as in baurusuchids in which an elevated crest posteriorly borders a broad basin-like depressed area of the frontals (e.g., *Stratiotosuchus* URC R73, *B. salgadoensis*, *Pissarrachampsa*). The posteromedial end of this crest forms part of the anteromedial corner of the supratemporal fossa so that the frontal barely enters into the fossa, as in most notosuchians (*Notosuchus*, *Comahuesuchus*, *Mariliasuchus*, *Yacarerani*, *C. paulistanus*, and *C. montealtensis*), except for some basal forms that have a much larger participation of the frontal in the supratemporal fossa (*Montealtosuchus*, *A. buitreraensis*). Posterior to this point, the frontal is sutured to the parietal along an interdigitated and obliquely oriented suture. Only the lateral most region of this suture has been preserved in the right side of MZSP-PV 139. The ventral surface of the frontal bears well developed *crista cranii* that bound the passage of the olfactory tract. This tract has its minimum width along the posterior half of the orbit and expands anteriorly (to house the olfactory bulbs) and posteriorly (to house the anterior part of the cerebral hemispheres).

Only fragments of the parietal have been preserved within the right lateral supratemporal fossa. The preserved right anterolateral end of the parietal indicates this element was extensively sutured to the postorbital within the fossa and therefore excluded the frontal from the supratemporal fenestra.

The right squamosal is almost completely preserved but the crushing suffered by this specimen precludes determining some details of its sutural contacts within the otic recess. As in most basal mesoeucrocodylians, the squamosal has three distinct processes in dorsal view: an anterior process that forms the lateral border of the supratemporal opening, a posteromedial process that forms the lateral half of the posterior edge of this opening, and a posterolateral process that is ventrally deflected (Figures 9, 11). The anterior process is relatively long and meets the postorbital along an interdigitated and transeversely oriented suture located at the anteroposterior midpoint of the supratemporal fenestra. The dorsal surface of the anterior branch of the squamosal is divided into a medial smooth region that forms the lateral surface of the supratemporal fossa and a lateral ornamented region that contributes to the skull roof (Figure 9). The lateral region is deflected ventrally towards its lateral margin so that the ornamented region of the anterior region of the squamosal faces dorsolaterally (Figures 9, 11, 17). This differs from the generalized condition of crocodyliforms, present in numerous notosuchians (e.g., *Notosuchus*, *Simosuchus*, *Araripesuchus*, *Candidodon*), which have a dorsally facing anterior branch of the squamosal that forms part of the flat and horizontal skull roof. This ventral deflection of the outer region of the squamosal covers most of the deep otic recess in lateral view (Figures 11, 17) that is described in further detail below. The CT slices show that the anterior branch of the squamosal encloses a single elongated cavity located anteriorly to the level of the otic aperture (Figure 13M).

The posteromedial process of the squamosal is short and transversely directed forming an angle of approximately 90 degrees with the anterior branch of the squamosal (Figure 9). This branch is divided into a posteriorly located region that is ornamented and dorsally exposed and an anteriorly located region that is smooth and forms the posterior surface of the supratemporal fossa. The ornamented posterior region of this process extends medially up to the lateromedial midpoint of the external margin of the supratemporal fossa. Although the parietal has not been preserved in this region, the medial edge of this process has an interdigitated sutural end that represents its contact with the parietal. The smooth anterior region of the posteromedial process of the squamosal that forms the posterior surface of the supratemporal fossa extends further medially, reaching the posteromedial corner of this fossa (Figure 9). This corner, however, is poorly preserved and its sutural contact with the parietal (as well as the position of the anterior opening for the temporo-orbital artery) cannot be determined with certainty. A small notch on the posterior end of the smooth surface of the squamosal may represent a temporo-orbital foramen (Figure 9), which would be completely enclosed by the squamosal, a derived condition present in all sphagesaurids that preserve this region (i.e., *C. montealtensis*, *Yacarerani*, *Armadillosuchus*) as well as in *Mariliasuchus* and some baurusuchids (*B. salgadoensis*, *Stratiotosuchus*). The posterior margin of the posteromedial process of the squamosal is sutured to the supraoccipital and exoccipital along the dorsal margin of the occipital table (Figure 18).

At the level of the occipital margin of the skull, the ornamentation on the dorsal surface of the squamosal ends abruptly (Figures 9, 17). The posterolateral process of the squamosal originates at this point, being slightly recessed with respect to the level of the ornamented skull roof in lateral view (Figure 17A). The posterolateral process directs posteroventrally forming an angle of approximately 45 degrees with the longitudinal axis (Figure 11). A similar orientation is present in most notosuchians, including some peirosaurids (*Lomasuchus*, *Montealtosuchus*). In *C. montealtensis* (but not in *C. paulistanus*) as well as in most basal mesoeucrocodylians, however, the posteroventral processes is slightly less ventrally deflected (e.g., *A. wegeneri*, *A. patagonicus*, *Anatosuchus*). Baurusuchids have this process directed almost ventrally, forming an angle of approximately 90 degrees with the longitudinal axis of the skull [29]. The lateral deflection of this process is minimal in *C. stenognathus* (Figure 9), although this feature might have been affected by the crushing of the specimen.

The dorsal surface of the posterolateral process is relatively broad and long, being approximately as broad as the anterior branch of the squamosal (Figure 9). Its external surface is smooth and slightly convex along the lateromedial axis, as in several advanced notosuchians (*Mariliasuchus*, *Notosuchus*, *Yacarerani*, *C. montealtensis*) and baurusuchids. This morphology contrasts with the ornamented posterolateral process of *Armadillosuchus*, which is the generalized condition found in more basal notosuchians (e.g., *A. wegeneri*, *A. gomesii*, *Anatosuchus*, *Simosuchus*, peirosaurids), sebecids, and basal crocodyliforms (e.g., *Zosuchus*, *Fruitachampsa*). The dorsal surface of the posterolateral process bears a slightly marked and curved ridge that extends obliquely along the anterior half of this process. This ridge originates at the anteromedial corner of this process and ends at the anteroposterior midpoint of its lateral margin (Figure 17A). The distal half of the posterolateral process of the squamosal is almost as broad as its anterior region, ending in a broad and rounded distal end in dorsal view (Figure 9) and located caudally to the level of the quadrate condyles in lateral view (Figure 17A). The latter character seems to be present in several notosuchians, except for baurusuchids that have a highly modified posterolateral process of the squamosal [29]. In other mesoeucrocodylians the posterolateral process of the squamosal does not exceed the caudal margins of the quadrate condyles, such as neosuchians, mahajangasuchids, peirosaurids, and more basal forms (e.g., *Zosuchus*, *Fruitachampsa*). The medial margin of the posterolateral process of the squamosal is sutured to the dorsal margin of the exoccipital up to the distal end of the paroccipital process (Figure 18). At the distal end of posterolateral process of the squamosal, its medial edge dorsally overlaps the prominent crest of the posterior surface of the quadrate (Figure 18; see below).

The morphology of the squamosal at the otic recess area bears some derived similarities with some advanced notosuchians and baurusuchids. First, as noted above, the lateral margin of the squamosal is ventrally deflected, especially along the posterolateral process of this bone. Therefore, the posterior region of the otic recess of MZSP-PV 139 is hidden in lateral view (Figure 11). The oblique crushing of the specimen could have accentuated this condition but, nonetheless, a similar morphology is present in *Baurusuchus* and several advanced notosuchians (e.g., *Mariliasuchus*, *Yacarerani*, *Notosuchus*), and to a lesser degree in other notosuchians (e.g., *Armadillosuchus*, *Araripesuchus*, peirosaurids). This region is damaged in the type specimens of *C. montealtensis* and *C. paulistanus*. Neosuchians usually lack a ventrally deflected outer region of squamosal and the posterior region of the otic recess is completely exposed in lateral view.

Second, the outer margin of the squamosal has a sinuous outline in lateral view (Figure 11). This margin has a convex outline along the extent of the posterolateral process of the squamosal, a short and markedly concave region at the level of the otic aperture, and a convex outline that extends up to the anterior end of the squamosal. This condition has been noted as a derived feature shared by *Mariliasuchus* and *Yacarerani* [15]. A similar morphology, however, is also present in baurusuchids (*Stratiotosuchus*, *B. salgadoensis*, *B. albertoi*) and incipiently developed in *Notosuchus* and *Armadillosuchus*. The condition of other sphagesaurids such as *S. huenei*, *C. montealtensis*, *C. paulistanus*, and *Adamantinasuchus* is currently unknown for this character.

Third, a thin ridge extends longitudinally close to the sinuous outer margin of the squamosal. This ridge is straight to slightly curved and runs from the posterior end of the posterolateral process up to the anterior end of the squamosal (Figures 11, 17). A smooth and slightly concave surface extends between this ridge and the sharp edge of the squamosal. The marginal concave surface bounded dorsally by a ridge suggests this area was the attachment of the ear-flap musculature, as in extant crocodylians [49]. The dorsal ridge is more prominent along the posterolateral process of the squamosal, where the ear-flap groove is deepest and broadest. The groove disappears at the level of the otic aperture (where the outer edge of the squamosal is concave) and reappears anteriorly, being dorsoventrally shorter and shallower than the posterior region of the groove (Figure 17A). Along this anterior region the ridge forms the ventrolateral limit of the ornamented surface of the squamosal. The morphology of the ear-flap groove of *C. stenognathus* resembles the condition of advanced notosuchians (*Notosuchus*, *Mariliasuchus*, *Yacarerani*, *Armadillosuchus*) in being broad, shallow, and facing dorsolaterally. This differs from the generalized condition of most crocodyliforms (including sebecids, baurusuchids, peirosaurids, mahajangasuchids, *A. wegeneri*, *Anatosuchus*, *Simosuchus*, *Candidodon*), in which the groove is thin and deep and faces laterally rather than dorsolaterally. The condition of *C. montealtensis*, *C. paulistanus*, *S. huenei*, and *Adamantinasuchus*, however, is currently unknown.

Fourth, the ventral surface of the lateral region of the squamosal is markedly concave and forms a vaulted trough-shaped roof of the otic recess (Figure 17B). This morphology extends along the entire length of the squamosal, from its posterolateral end to its anterior contact with the postorbital. This concave roof of the otic recess, in fact, continues into the ventral surface of the postorbital (see below; Figures 13L–M). The ventral surface of the squamosal bears a broad ridge that extends anteromedially from its lateral margin (at the level anteroposterior midpoint of the posterolateral process) towards the otic aperture (Figure 17B–C). Medially from this ridge the ventral surface of the squamosal has faint longitudinal striations. In ventral view, the squamosal ridge located on the roof of the otic recess delimits the lateral extent of a distinct groove that broadens posteriorly and opens at the distal end of the posterolateral process of the squamosal (Figure 17C). Thus, the otic recess opens posteroventrally, well below the level of the otic aperture (Figure 17). A similar posteroventral extension of the otic recess is present in most notosuchians (including *Lomasuchus*, *Uberabasuchus*, *Montealtosuchus*) and in *Fruitachampsa*, all of which have posterolateral processes of the squamosal that extends the otic recess posteroventrally from the otic aperture. In some of these taxa (e.g., *A. wegeneri*, *Anatosuchus*, *Simosuchus*) the posterolateral process of the squamosal is not as ventrally deflected as in *C. stenognathus* (or baurusuchids). However, all of them clearly differ from the condition of *Mahajangasuchus*, *Kaprosuchus*, *Hamadasuchus*, *Stolokrosuchus*, and neosuchians, in which the posterior opening of the otic recess is located more dorsally, at the same level of the otic aperture (e.g., *Shamosuchus*, *Allodaposuchus*).

The postorbital has a dorsal surface contributing to the skull roof and an inset descending process that forms part of the otic recess and the postorbital bar (Figures 9, 11). The dorsal surface of the postorbital is slightly sculpted and forms the anterolateral margins of the supratemporal fossa and fenestra. Within the fossa the postorbital is smooth and sutured to the parietal medially and squamosal posteriorly. The sculpted dorsal surface of the postorbital is narrow, but not as reduced as in *Mariliasuchus* and *Yacarerani*. The dorsal ornamented surface is interrupted at the level of the anterolateral margin of the supratemporal fenestra by a transversely oriented groove (Figures 16, 17), so that the anterolateral rim of the supratemporal fossa is absent at this point. This unusual feature is present but more developed in *C. montealtensis* (and possibly in *C. paulistanus* but this region is poorly preserved in the holotype) and incipiently present in *Armadillosuchus*. The condition of *Caryonosuchus* and *S. huenei* is unknown but this groove is absent in the anterolateral corner of the supratemporal fossa in *Yacarerani* and *Adamantinasuchus*, as well as in other advanced notosuchians (e.g., *Mariliasuchus*, *Notosuchus*) and baurusuchids.

The outer margin of the dorsal surface of the postorbital extends obliquely to the longitudinal axis of the skull as in most notosuchians [27], including peirosaurids and mahajangasuchids (but not *Stolokrosuchus*). A small rounded smooth facet is located anteriorly to this margin, recessed from the dorsal surface of the postorbital (Figure 11). This area is interpreted as the articular facet for the posterior palpebral, which has not been preserved in MZSP-PV 139. This smooth recessed surface extends posteriorly along the lateral surface of the postorbital (Figure 11). This posterior extension is separated from the dorsal ornamented surface of the postorbital by a longitudinal ridge and its posterior end extends ventrally to the anterior end of the ear-flap groove of the squamosal, being medially inset with respect to it (Figure 17B). The morphology of this posterior extension seems to be an autapomorphic feature of *Caipirasuchus stenognathus*, although this area is damaged in the type specimens of both *C. paulistanus* and *C.montealtensis*.

This recessed smooth surface of the postorbital forms the anterior and anterolateral limits of the otic recess. Thus, the otic recess of *C. stenognathus* extends anteriorly up to the anteriormost region of the postorbital, dorsally to the origin of the postorbital bar meets the jugal (Figure 17A), a condition present in all notosuchians (including peirosaurids and mahajangasuchids [24]). The anterior area of the otic recess is deep, with the postorbital forming its concave roof (Figure 13L), and only partially exposed in lateral view (Figure 17), as in the posterior region of this recess. In contrast to the caudally open posterior end of the otic recess (at the distal end of the squamosal posterolateral process), the anterior margin of the otic recess is closed by a descending lamina of the postorbital that extends transversely below the articular facet for the posterior palpebral (Figure 17B). Such an extensive and anteriorly closed rostral end of the otic recess is present in all notosuchians (including baurusuchids, sebecids, mahajangasuchids, and peirosaurids). Other mesoeucrocodylians, instead, have a more restricted anterior end of the otic recess [24], which is limited to the squamosal or only part of the postorbital and does not extend above the postorbital bar. The postorbital-squamosal suture within the otic recess cannot be precisely identified due to the crushing of the specimen.

The descending bar of the postorbital extends anteroventrally from the otic recess as a lateromedially thin and anteroposteriorly elongated lamina that tapers ventrally. The postorbital bar becomes subcylindrical close to its contact with the jugal located at the dorsoventral midpoint of the orbit. At this contact, the jugal laterally overlaps the postorbital as in most mesoeucrocodylians and together form the posterior margin of the orbit.

The jugal is triradiate as in all crocodyliforms and its external surface faintly ornamented with shallow short grooves (Figures 11, 19). The suborbital region of the jugal is dorsoventrally expanded with respect to the infratemporal bar, as in all mesoeucrocodylians [27]. The anterior end of the jugal is expanded with respect to the suborbital region of the jugal, although it lacks an expanded anteroventral corner as in most advanced notosuchians (including *C. montealtensis* and *C. paulistanus*). This differs from the condition of *S. huenei* and baurusuchids that, as noted above, have a blunt posteroventral process of the maxilla and a large anteroventral expansion of the jugal. The jugal has a short triangular process at its anteroventral end that wedges between the posteroventral end of the lateral surface of the maxilla and the anterior process of the ectopterygoid. This character that was described as diagnostic of *C. montealtensis* [10], but it is now known to be present in the three species of *Caipirasuchus*.

The anterodorsal corner of the jugal surpasses the anterior edge of the orbit (Figure 19), as in most mesoeucrocodylians. As noted above, this region of the jugal is sutured to the maxilla and fails to reach the lacrimal, contrasting with the condition of *C. paulistanus* and *C. montealtensis* that have a distinct anterodorsal process in the jugal that is sutured to the ventral end of the lacrimal.

The anterior region of the jugal of *C. stenognathus* has a ridge that runs along the orbital margin, located well above the jugal foramen (Figure 19), resembling the condition of *C. montealtensis*, *C. paulistanus*, *Mariliasuchus*, *Notosuchus*. The ornamention of this part of the jugal is restricted to the area of the ridge. The suborbital region of the jugal of other sphagesaurids (e.g., *Yacarerani*, *S. huenei*) also has a longitudinal crest but differs in that it runs on the lateral surface of the jugal, right above the jugal foramen, and overhangs a depressed anteroventral region of the jugal. This anteroventral depression is also present in baurusuchids. The longitudinal ridge of *C. stenognathus* extends posteriorly up to the level of the ascending process of the jugal, dividing the external ornamented surface of the jugal from the smooth dorsal and medial surface of the suborbital region of the jugal.

The lateral surface of the suborbital region of the jugal bears a large jugal foramen that opens in an ovoid fossa (Figure 19). Additionally, a small foramen is associated with the large jugal foramen, being located anteriorly on the left side (Figure 19) and posteriorly on the right side (Figure 11). A large jugal foramen located on the lateral surface of the anterior region of the jugal is also present in most advanced notosuchians (e.g., *Mariliasuchus*, *Yacarerani*, *Adamantinasuchus*, *C. montealtensis*, *S. huenei*), some specimens of *Notosuchus* (MLP 64-IV-16-9, MACN-PV RN1041), and *Comahuesuchus*; but not in baurusuchids, sebecids, peirosaurids, mahajangasuchids, or other basal notosuchians (*Araripesuchus*, *Anatosuchus*, *Simosuchus*, *Candidodon*). CT slices show that the large jugal foramen is the anterior opening (Figure 13H) of a large duct that extends posteriorly along the jugal (Figure 13I). This longitudinal passage has two accessory and small transverse ducts that open through two foramina located on the medial surface of the jugal (anterior to the postorbital bar; Figure 9). The large longitudinal duct bifurcates at the level of the ascending process of the jugal into a dorsomedial and a lateroventral branches (Figure 13J). The dorsomedial branch exits through a foramen located at the posterior surface of the base of the ascending process of the jugal (Figures 9, 13K) and the lateroventral branch extends posteriorly along the infratemporal bar of the jugal gradually reducing its diameter. This branch seems to obliterate at the anteroposterior midpoint of the infratemporal bar. This series of openings and ducts that run across the jugal may represent passages and ramifications of the jugal branch of the maxillary nerve (V_2_), which has a similar anterior exit on the lateroventral margin of the jugal of extant crocodylians [45].

The ascending process is located at the anteroposterior midpoint of the jugal and its base directs posterodorsally, as in most notosuchians. Its base is anteroposteriorly broad and laminar becomes cylindrical towards its contact with the postorbital, resembling the condition of advanced notosuchians (*Adamantinasuchus*, *Yacarerani*, *S. huenei*, *C. montealtensis*, *C. paulistanus*). The lateral surface of the ascending process is continuous with the external surface of the jugal, a plesiomorphic condition among mesoeucrocodylians [27]. Its entire surface is smooth as in most mesoeucrocodylians. The jugal extends posteriorly to the ascending process forming the infratemporal bar of the jugal (Figures 10, 11). This process is dorsoventrally flattened and has a sculpted lateral surface. The posterior end of the jugal has not been preserved in articulation with the quadratojugal, but it can be inferred that the jugal laterally overlapped the quadratojugal and that it extended up to the posterior margin of the infratemporal fenestra.

Only the right quadratojugal has been preserved in MZSP-PV 139 (Figure 11), disarticulated from the quadrate and jugal and rotated 90 degrees from its natural position. The quadratojugal is smooth and forms the posteroventral corner of the infratemporal fenestra. The anterior branch of the quadratojugal is short and almost entirely overlapped by the jugal (Figure 11). The ascending process of the quadratojugal is only partially preserved, but given the articular surface of the quadrate, it seems to have reached the postorbital close to the apex of the infratemporal fenestra. The posteroventral corner of the quadratojugal is rounded and, based on the articular surface for this bone on the quadrate, it would have been positioned well above the level of the quadrate condyles. These features, the rounded shape of the postervonventral corner of the quadratojugal and its position far from the quadrate condyles, is present in all known sphagesaurids (*C. montealtensis*, *C. paulistanus*, *S. huenei*, *Armadillosuchus*, *Yacarerani*) and other notosuchians (e.g., *Mariliasuchus*, *Notosuchus*, *Malawisuchus*, *Pakasuchus*) and baurusuchids, but is absent in the most basal notosuchians (*Araripesuchus*, *Simosuchus*, *Candidodon*, *Libycosuchus*), as well as in peirosaurids and sebecids.

The right quadrate has been completely preserved whereas only the articular region is present of the left quadrate in MZSP-PV 139. The distal body of the quadrate is ventrally directed in lateral and posterior views (Figures 11, 18), as in notosuchians (including baurusuchids but not peirosaurids, mahajangasuchids, or sebecids). The quadrate condyles face ventrally and are located below the level of the upper toothrow (Figure 11), two features shoared by most notosuchians (whereas others like mahajangasuchids and some peirosaurids have condyles ventrally placed respect to the upper toothrow but that face posteroventrally). The medial quadrate condyle projects ventrally and ends in a transversely narrow and sharp ventromedial edge (Figure 12). The articular surface of the lateral condyle differs from that of the medial condyle in being rounded, lateromedially broader, and located more dorsally. In *C. paulistanus* this difference between the quadrate condyles, especially the ventral projection of the medial condyle is much more developed (and in *C. montealtensis* the articular condyles have not been preserved). This more extensive ventral projection of the medial quadrate condyle in *C. paulistanus* results in that the distal body of the quadrate is dorsoventrally taller with respect to its lateromedial width than in *C. stenognathus*. The quadrate condyles are separated by a deep intercondylar groove, as in most notosuchians (including baurusuchids), including at least some peirosaurids (e.g., *Montealtosuchus*, *Lomasuchus* MCF-PVPH 160). The lateromedial width of the distal body of the quadrate is approximately twice as broad as anteroposteriorly long, resembling the morphology of most notosuchians (*Araripesuchus*, *Simosuchus*, *Anatosuchus*, *Yacarerani*, *Mariliasuchus*, *C. paulistanus*). In contrast, *S. huenei* and baurusuchids have an anteroposteriorly broader distal body of the quadrate. Unfortunately, the condition of *C. montealtensis* and *Adamantinasuchus* is currently unknown. The distal body of the quadrate of *C. stenognathus* (and other notosuchians) is, however, not as anteroposteriorly narrow as in neosuchians, in which the lateromedial width is approximately three times its anteroposterior length.

The posterior surface of the distal body of the quadrate has a remarkably well-developed crest that extends from the posterior margin of the medial condyle to the posteromedial corner of the posterolateral process of the squamosal (Figure 18). At its dorsal end, the crest bears a rounded surface, ventrally bouded by a striated surface. Such a highly developed crest is an uncommon feature shared with *C. paulistanus* and probably with *C. montealtensis* [10], but in the holotype of the latter taxon the distal end of this crest is not preserved. In most notosuchians and baurusuchids this crest is present but much more faintly developed (e.g., *Araripesuchus*, *Libycosuchus*, *Mariliasuchus*, *Notosuchus*, *Yacarerani*, *S. huenei*). This crest laterally bounds a shallow triangular concavity on the posterior surface of the quadrate (Figure 18), which is also present in notosuchians, including baurusuchids [22]. The medial surface of this crest bears a small and elongated foramen aërum (*sensu* [50]), which is located on the posterior surface of the quadrate (Figure 18). Most notosuchians (and baurusuchids) have a medially located foramen aërum, although some basal notosuchians (e.g., *Anatosuchus*) have this opening more centrally located on the posterior surface of the quadrate.

As in all mesoeucrocodylians, the quadrate projects medially on the occipital region of the skull and is extensively sutured to the exoccipital. This suture extends ventromedially from the region of the paroccipital process to the basal tubera, at the point of triple contact between the quadrate, exoccipital, and basioccipital (Figures 18, 20). The oblique crushing of the specimen has damaged the region of the paroccipital contact so that the posterior opening of the cranio-quadrate passage cannot be observed. Nonetheless, the quadrate and squamosal meet posterolaterally from the distal end of the paroccpital process (and consequently, from the cranio-quadrate opening). The quadrate of *C. stenognathus* lacks the well-developed crest present in *S. huenei* and *Armadillosuchus* that extends medially along the ventral margin of the occipital region of the skull, which excludes the exoccipital from this margin.

The ventral surface of the right quadrate of MZSP-PV 139 is crushed, but the pterygoid processes of both quadrates have been preserved without damage (Figure 20). This process is sutured to the ventrolateral margins of the basal tubera of the basioccipital and to the posterodorsal half of the lateral margin of the basisphenoid. A quadrate-basioccipital contact is a derived condition shared by *Simosuchus*, baurusuchids, and advanced notosuchians (most sphagesaurids, *Notosuchus*, *Mariliasuchus*), although there is variation within Sphagesauridae regarding the existence and nature of this contact. In *S. huenei* and *Armadillosuchus* the quadrate-basioccipital suture is exposed on the occipital surface of the skull, whereas in the three species of *Caipirasuchus* is located on the ventral surface of the skull (the condition found in baurusuchids, *Simosuchus*, *Notosuchus*, *Mariliasuchus*, and mahajangasuchids). Finally, a quadrate-basioccipital suture is absent in *Yacarerani* (resembling the condition of *Araripesuchus* and peirosaurids). At this point, the pterygoid process of the quadrate contacts the quadrate process of the pterygoid through an interdigitated suture (Figure 20). The long extension of the pterygoid process of the quadrate along the lateral surface of the basisphenoid, leaving the pterygoid well separated from the lateral Eustachian openings is a derived feature of sphagesaurids (*C. montealtensis*, *C. paulistanus*, *S. huenei*, *Armadillosuchus*), and is extremely developed in *Yacarerani*. Lateral to the suture with the basisphenoid, and dorsally to the suture with the pterygoid, the quadrate bears two well-developed marks of muscular insertion. These face laterally and are located on the pterygoid process of the quadrate. The most ventral of these is a long and thin ridge that extends posterodorsally from the lateral surface of the quadrate-pterygoid suture (Figure 20). The other area of muscular insertion is located dorsally to the posterior end of the ridge described above and is a rounded and rugose bulge (Figure 20). These two structures were probably the area of insertion for the adductor musculature (m. adductor mandibulae posterior *sensu* [45]) and given their relative position and morphology could be homologous to the adductor crests A and B described for extant crocodylians [50]. The distribution of this feature among notosuchians is not clear, but in *C. paulistanus* there is a single crest located in this region and *C. montealtensis* seems lack both of them at least in the preserved region of the pterygoid process of the quadrate. *Mariliasuchus* and *Yacarerani* seem to have also only one of these crests, as in *C. paulistanus*. Dorsally to this region, the contact of the quadrate with the lateral wall of the braincase has not been preserved due to the oblique crushing of this specimen.

The right quadrate has preserved the dorsal articular process, which is anterodorsally directed and exposed laterally on the otic recess (Figures 11, 17). The dorsal edge of this region is extensively sutured to the squamosal. This suture runs posteroventrally from the otic aperture to the dorsal limit of vertical crest of the posterior surface of the quadrate (Figure 17A). The otic aperture interrupts this suture and presumably continues anteriorly to it, although this region is crushed and details on the quadrate-squamosal suture anterior to the otic aperture cannot be determined in *C. stenognathus*. The quadrate forms the entire ventral margin of the otic aperture and has a small but distinct depression (periotic fossa) that surrounds this margin (Figure 11). The ventral and posterior extent of the periotic fossa of *C. stenognathus* seems to be smaller than that of most notosuchians (*Araripesuchus*, *Simosuchus*, *Anatosuchus*, *Mariliasuchus*, *Notosuchus*) and resemble the reduced posteroventral region of the periotic fossa of *Yacarerani* and baurusuchids (*B. pachecoi*, *B. albertoi*). However, the anterior region of the periotic fossa is more extensive in *Yacarerani* (and other notosuchians) but is not preserved in *C. stenognathus*. The size of the periotic fossa of other sphagesaurids (e.g., *Adamantinasuchus*, *S. huenei*, *Caryonosuchus*, *C. montealtensis*, *C. paulistanus*) is currently unknown.

A small and elongated foramen is located at the anteroventral corner of the otic aperture, within the periotic fossa. The crushing of the specimen precludes determining with certainty how many accessory foramina within the periotic fossa, a feature that shows variation within advanced notosuchians (e.g., *C. montealtensis*, *Yacarerani*, *Mariliasuchus*, *Notosuchus*). Small foramina (located in a reduced periotic fossa) are also present in baurusuchids but are internalized in the otic aperture in most of them (*Baurusuchus*, *Stratiotosuchus*) [30]. As discussed above, the morphology of the periotic fossa in other sphagesaurids (except for *Yacarerani*) is currently unknown. However, all advanced notosuchians and baurusuchids for which this region is known share the presence of accessory foramina in the periotic region, which contrasts with the absence of these features in sebecids, peirosaurids, mahajangasuchids, and basal notosuchians (e.g., *Araripesuchus*, *Anatosuchus*, *Uruguaysuchus*, *Malawisuchus*, *Simosuchus*), as well as neosuchians. The presence of numerous and large accessory foramina has been also described for several basal crocodyliforms distantly related to these derived notosuchians [27], [51]–[53]. Anterior to the otic aperture (and periotic fossa), the quadrate projects anterodorsally to contact the postorbital within the otic recess. This region of the quadrate of *C. stenognathus* has a small anterior siphoneal foramen (Figure 17B). It is not clear if this foramen is included within the periotic fossa. The anteroventral margin of this projection has a sutural contact for the ascending process of the quadratojugal (Figure 17B).

Only part of the right laterosphenoid has been preserved in *C. stenognathus*, extending dorsally to the anterior process of the quadrate (Figure 20). The lateral surface of the laterosphenoid bears a robust ridge, as in neosuchians (including extant crocodylians) but contrasting with the rounded lateral surface of basal crocodyliforms (e.g., *Zosuchus* [54]). This ridge extends posteroventrally from the capitate processs to the dorsoventral midpoint of the preserved region of the laterosphenoid and divides the anterior from the lateral surface of this bone. Unfortunately, this bone has not preserved details on the openings for the cranial nerves II, III, or IV. The capitate process of the laterosphenoid is sutured to the medial surface of the postorbital and probably to the anterolateral projection of the parietal.

Most of the supraoccipital has not been preserved, however the right dorsolateral region of this bone is visible in occipital view (Figure 18). The preserved region is lateroventrally sutured to the exoccipital and dorsally bounded by the squamosal. Based on the position of the dorsolateral corner of the supraoccipital, the lateromedial width of this bone must have occupied half of the dorsal margin of the occipital table, as in notosuchians (including peirosaurids and *Mahajangasuchus*) and basal crocodyliforms. Neosuchians, instead, have a much narrower supraoccipital that forms less than 30% of the dorsal margin of the occipital table. The dorsal margin has a small posteriorly projected process with a rugose dorsal surface, resembling the morphology of the supraoccipital at the ventral margin of the post-temporal fenestra in other crocodyliforms [50]. The ventral margin of this fenestra in *C. stenognathus* is located at the level of the dorsal surface of the skull roof (Figure 18). This condition is shared by most notosuchians (e.g., *A. gomesii*, *Notosuchus*, *B. salgadoensis*), but differs from that of extant eusuchians in which the supraoccipital contribution to this opening is ventrally recessed with respect to the level of the skull roof.

Most of the right exoccipital has been preserved in MZSP-PV 139 (Figure 18). The left element is much more fragmentary, having preserved only its ventromedial region along its contact with the basioccipital and quadrate. The posterior surface of the exoccipital has a broad transverse ridge that runs medially from the paroccipital process, above the level of the foramen magnum. Dorsal to this ridge the exoccipital is triangular shaped and faces posterodorsally and ventral to this ridge it faces posteroventrally, as in most notosuchian crocodyliforms. The dorsomedial margin of the dorsal region of the exoccipital is sutured to the supraoccipital and reaches the dorsal margin of the occipital table, where the exoccipital ends in a lateromedially narrow apex (Figure 18). The dorsolateral margin of the exoccipital is sutured to the posterolateral process of the squamosal and extends posterolaterally up to the dorsal margin of the paroccipital process. This process is poorly preserved in MZSP-PV 139 but seems to end in a dorsoventrally narrow and rounded distal end (Figure 18), as in *C. montealtensis*. The distal end of the paroccipital process is placed anteriorly and medially to the distal end of the posterolateral process of the squamosal (that meets the dorsal end of the quadrate posterior crest). As described above, the ventrolateral margin of the exoccipital is extensively sutured to the quadrate along an overlapping contact that extends from the paroccipital process to the basal tubera of the basioccipital. The medial region of this suture forms the ventral margin of the occipital table, so that the exoccipital reaches the ventral margin of the occiput as in *C. montealtensis* (and most crocodyliforms) but differing from *S. huenei* and *Armadillosuchus* in which the quadrate-basioccipital contact excludes the exoccipital from the ventral margin of the occiput. The suture with the basioccipital runs dorsomedially from the tubera to the occipital condyle. The exoccipitals form the dorsolateral region of this condyle and extend dorsally from it, forming the margins of the foramen magnum (Figure 18), as in all crocodyliforms. The posterior exit for the cranial nerves IX-XI is crushed and therefore the number and position of these foramina cannot be determined in *C. stenognathus*. The region lateral to the foramen magnum, where the exits of the cranial nerve XII are located in other crocodyliforms, has not been preserved. The ventral region of the exoccipital lacks a foramen for the internal carotid artery (Figure 18, 20B), resembling the condition of advanced notosuchians (*Notosuchus*, *Mariliasuchus*, *C. montealtensis*, *Yacarerani*), baurusuchids, and some peirosaurids (e.g., *Montealtosuchus*) in which the entrance of the internal carotid artery is located within the same depression as the exit for the cranial nerves IX-XI. This position contrasts with the generalized condition found in more basal notosuchians (*Simosuchus*, *A. gomesii*, *A. wegeneri*, *Anatosuchus*), *Sebecus icaeorhinus*, basal crocodyliforms, and neosuchians, in which the entrance of the internal carotid artery is located close to the ventral tip of the exoccipital.

The basioccipital forms most of the occipital condyle and its ventral surface faces posteroventrally (Figures 18, 20). The condyle is hemispherical and has a well-developed neck, especially on the ventral surface. Ventral to the occipital condyle, the posterior surface of the basioccipital bears a median small rounded foramen. The basal tuberae are poorly developed, being slightly elevated and rugose areas located at the ventrolateral corners of the basioccipital along the sutures with the exoccipital and pterygoid process of the quadrate (Figure 20). Ventral to the small foramen, the basioccipital is flat and only has a faintly developed sagittal ridge that extends up to the ventral edge of this bone. This crest is much more developed in other notosuchians, except for *Armadillosuchus* and *S. huenei*. The basioccipital-basisphenoid suture is preserved only lateral to the lateral Eustachian foramina. The basioccipital ventral edge has a slightly developed transverse ridge that is interrupted by two small notches, one located at the ventrolateral corner of the left side and another in the midline of the basioccipital (Figure 20). These notches are the posterior margins of the left lateral Eustachian foramen and the median foramen intertympanicum. These two foramina are located at the same level, both anteroposteriorly and dorsoventraly, as in other sphagesaurids in which this region is known (*Yacarerani*, *Armadillosuchus*, *S. huenei*). In most notosuchians (including baurusuchids, sebecids, peirosaurids, and mahajangasuchids) the Eustachian foramina are posteriorly located with respect to the foramen intertympanicum. In *Mariliasuchus* the posterior displacement of the Eustachian foramina is not as marked as in other notosuchians but nonetheless they are not aligned as in *C. stenognathus*.

Most of the ventral surface of the basisphenoid is broken and has collapsed, but its lateral and anteriormost regions have been preserved (Figure 20). Based on the lateral and anterior margins, the ventral exposure of the basisphenoid was rounded and remarkably large, being slightly longer than broad (although the crushing of the specimen have likely affected this proportion). Although basal crocodyliforms are known to have an expanded basisphenoid [27], most notosuchians have a relatively small and V-shaped ventral exposure of the basisphenoid (e.g., *Araripesuchus*, *Anatosuchus*, *Simosuchus*, *Notosuchus*, *B. salgadoensis*). The ventral surface of the basisphenoid of *Mariliasuchus* and sphagesaurids that have preserved this region (*S. huenei*, *Armadillosuchus*, *Yacarerani*), however, also has the unusually extensive and rounded shape present in *C. stenognathus*. Although the basisphenoid of *C. montealtensis* is not complete, its right lateral margin is complete and shows this condition too. The preserved ventral surface of the basisphenoid bears two low oblique ridges that converge anteriorly and extend for at least the anterior half of the bone (Figure 20). The central region of the basisphenoid that is enclosed within these ridges is crushed and collapsed (Figures 20, 21). Similar ridges are present in *Mariliasuchus*, *Notosuchus*, baurusuchids, and basal notosuchians (e.g., *Araripesuchus*, *Uruguaysuchus*), but are absent in other sphagesaurids (*Yacarerani*, *Armadillosuchus*, and *S. huenei*). The anterior margin of the basisphenoid is sutured to the medial region of the quadrate processes of the pterygoid.

Both palatines have been preserved in MZSP-PV 139, although the left paplatine is partially crushed and its anterior end is displaced under the palatal branch of the left maxilla (Figure 12). The anterior end of the palatine is sutured to the posteromedial corner of the palatal branch of the maxilla along suture that extends anteromedially from the anterior end of the suborbital fenestra. Thus, the anterior ends of the palatines extend between the right and left maxilla, as in most notosuchians. As noted above, this contact probably bears a small maxillo-palatine fenestra on each side, as in *Mariliasuchus* and *Notosuchus*. These openings are absent in *Yacarerani* and *S. huenei* and seem to be absent in *C. montealtensis* and *C. paulistanus*. In the latter two taxa, however, have a distinct depression at the anteromedial corner of the palatines and posteromedial region of the palatal branches of the maxilla. This depression is filled with sediment in the holotype of both taxa and therefore the presence of a fenestra cannot be confirmed at this moment. The anterior projection of the palatines between the palatal branches of the maxilla is present in all notosuchians, except for baurusuchids. The condition of *Comahuesuchus* and *S. huenei* is intermediate, having a poorly developed anterior process of the palatines. As in most notosuchians, the palatine does not contact the ectopterygoid on the anterior end of the suborbital fenestra. These two bones are separated by the short maxillary contribution to the anterolateral margin of the suborbital fenestra (Figure 12). This is the generalized morphology of notosuchians (present also in the two other species of *Caipirasuchus*) but contrasts with the palatine-ectopterygoid contact described for *Yacarerani* [15] and the condition of *Mariliasuchus*, in which the palatine and ectopterygoid are almost in contact at the anterior end of the suborbital fenestra [17].

Posterior to their contact with the maxilla, the palatines project between the suborbital fenestrae forming the floor of the nasopharyngeal duct (Figure 13H). This region of the palatines is relatively narrow and long (Figure 12), as in *C. montealtensis*, *C. paulistanus* and *S. huenei*, but not as elongated and narrow as in baurusuchids. In other advanced notosuchians (including the sphagesaurid *Yacarerani*) the palatines are anteroposteriorly shorter and lateromedially broader between the suborbital fenestrae (e.g., *Notosuchus*, *Mariliasuchus*). The lateral margins of the palatines converge posteriorly along the anterior half of the suborbital fenestra and then diverge from each other along the posterior half of this opening (as in *C. paulistanus* and *C. montealtensis*). The left and right palatines are tightly sutured to each other along the sagital plane up to the level of the anteroposterior midpoint of the suborbital fenestra. At this point the lateral surface of the palatines extends dorsally forming a laminar process that meets the anterior projection of the pterygoid (that form the dorsal roof of the nasopharyngeal passage anterior to the chonal opening).

Posterior to this region, the palatines form the anterior margin of the choanal opening and diverge posteriorly forming two posterolaterally projected palatine bars (*sensu* [17]). The palatine bar is a rod-like process that extends posterolaterally (and slightly ventrally) towards the pterygoid flanges, forming the lateral margins of the broad and triangular-shaped choanal depression (Figure 21). In contrast to the condition of most mesoeucrocodylians, the palatine is not sutured to the pterygoids in the choanal region, which form a narrow and anteroposteriorly oriented choanal groove (located dorsally and medially to the palatine bars; Figure 13I). Thus, the choanal depression is connected to the suborbital region through the space that separates the ventrally located palatine bar from the dorsally located pterygoid choanal groove (Figure 21). All these features are shared by advanced notosuchians in which this region has been preserved (*Notosuchus*, *Mariliasuchus*, *C. montealtensis*, *C. paulistanus*, *Yacarerani*) and by *Comahuesuchus* and baurusuchids (with certain modifications) and distinguish this group from the condition of all other crocodyliforms.

The distal end of the palatine bars is sutured to the ectopterygoid and pterygoid flanges (Figure 21). The ecopterygoid is sutured to the lateral margin of the palatine, from the posterior corner of the suborbital fenestra to the distal tip of the palatine bar (Figure 21). The pterygoid of *C. stenognathus*, therefore, is excluded from the posterior margin of the suborbital fenestra. The distal region of the palatine bar ventrally overlaps the anteromedial region of the pterygoid flanges (Figure 21). This type of contact between the palatine, ectopterygoid, and pterygoid is also present in most taxa that have palatine bars (*Comahuesuchus*, *Mariliasuchus*, *Yacarerani*, *C. paulistanus*, and *C. montealtensis*). The morphology of *Notosuchus*, however, is different because the palatine bar does not contact the pterygoid, as the ectopterygoid projects an anteromedial bar that meets the palatine bar along the lateral margin of the choanal depression [55]. This morphology is also present in baurusuchids (*Pissarrachampsa*, *B. pachecoi*, *B. salgadoensis*, *Stratiotosuchus*), which have a broad anteromedial projection of the ectopterygoid.

The ectopterygoids of MZSP-PV 139 are completely preserved and form the entire lateral margin of the suborbital fenestra with its major axis oriented anteroposteriorly (Figure 12). This condition is also found in *Mariliasuchus* and all sphagesaurids in which this region is known (*Yacarerani*, *C. montealtensis*, *C. paulistanus*) but differs from the generalized condition of mesoeucrocodylians in which the major axis of the ectopterygoid is oriented obliquely to the longitudinal axis of the skull and forms only the posterolateral margins of the suborbital fenestra. Such morphology is also found in *Notosuchus*, baurusuchids, sebecids, peirosaurids, mahajangasuchids, and other basal notosuchians (*Araripesuchus*, *Simosuchus*, *Anatosuchus*).

The anterior end has well developed anterior and posterior processes. The presence of well-developed anterior and posterior processes of the anterior end of the ectopterygoid is a plesiomorphic condition for notosuchians and is present in most sphagesaurids (*C. paulistanus*, *C. montealtensis*, *Yacarerani*) but contrasts with the derived condition of *S. huenei* that has a small and rounded anterior end of the ectopterygoid [5]. The posterior process overlaps the ventral surface of the jugal, located at the level of the jugal foramen (Figure 12). The anterior process is sutured to the posterior margin of the palatal branch of the maxilla and extends anteromedially, ending close to the maxilla-palatine suture at the anterior corner of the suborbital fenestra (Figure 12). The ectopterygoid-maxilla suture passes along the posteromedial border of the last maxillary alveolus as in *C. paulistanus*, *Mariliasuchus* and *Yacarerani*. In *C. montealtensis* (and most notosuchians) the ectopterygoid-maxilla suture passes close to the last maxillary alveolus but in *S. huenei* this suture is well separated from the alveolus (due to the reduced extension of the anterior process of the ectopterygoid).

The ectopterygoid projects posteroventrally from its anterior articular surface (Figures 10, 11) and forms a robust bar that is circular in cross section (Figure 13I). Towards its posterior contact with the palatine and pterygoid flange, the ectopterygoid expands lateromedially. This expanded region of the ectopterygoid overlaps the ventral surface of the anterolateral corner of the pterygoid flanges (Figure 21). The region that overlaps the pterygoid is lanceolated in ventral view, and slightly curved with the apex pointing poserolaterally. Along its lateral margin the ectopterygoid borders the ventral surface of the robust lateral suface of the pterygoid flange and bears slightly developed rugosities, as in most crocodyliforms. The medial margin of the lanceolated posterior end of the ectopterygoid is bordered by the pterygoid along its posterior half and by the lateral margin of the palatine bar along its anterior half (Figure 21). The right ectopterygoid has a small pointed process extends anteromedially from the posterior corner of the suborbital fenestra (Figure 21). This feature is absent in the left ectopterygoid, probably due to preservational causes. This process, however, differs from the well-developed and extensive anteromedial branch of the ectopterygoid that forms the posterolateral margin of the choanal opening of *Notosuchus* and baurusuchids.

The pterygoids are completely fused as in all mesoeucrocodylians [27] and the pterygoid flanges are directed lateroventrally, although the orientation was affected by the oblique crushing of the specimen (Figures 13L, 21B). The pterygoid flanges have an anteroposteriorly narrow base and expand gradually towards their lateral ends (Figure 21A). A similarly anteroposteriorly constricted medial end of the pterygoid flanges is present in *C. montealtensis*, but not in *C. paulistanus* that has the pterygoid flanges poorly constricted at its medial end (close to the choanal groove). The posterior margin of the flanges is formed by a thin ridge that extends along its entire length. The posterior region of the flanges is laminar (Figure 13L) but close to its anterior edge the flanges become dorsoventrally thicker given the presence of internal cavities (Figures 13J–K). The anterolateral corner of the pterygoid flange has a ventral expansion that forms an elevated platform that projects medially and is overlapped by the posterior end of the palatine bar (Figure 20). This platform is incipiently developed in *C. paulistanus* and *C. montealtensis* and in some specimens of *Notosuchus* (MACN-RN 1045, MUC-Pv 147), and *Mariliasuchus* (MZSP-PV 50, UFRJ R105), but none of them has the degree of development present in *C. stenognathus*. The ventral surface of the pterygoid flange is flat, except for a shallow concavity that runs from its anterolateral corner to the base of the elevated platform along the anterior margin of the pterygoid flange. This elongated concave surface is preserved only on the left side of *C. stenognathus*. As in *C. paulistanus*, the pterygoid flange lacks the deep and rounded parachoanal fossa described for *C. montealtensis* [10]. The lateral surface of the pterygoid flange is convex and rugose and tear-drop shaped in lateral view (Figure 12).

The morphology of the pterygoid flanges shares numerous characters with advanced notosuchians. First, the narrow base of the pterygoid flanges that expand distally is a widespread character among advanced notosuchians, being present in *Notosuchus*, *Mariliasuchus*, and *C. montealtensis* (but not in *C. paulistanus*). In *S. huenei*, the lateral region of the flanges is not preserved but their base is lateromedially narrow. In *Yacarerani* the base of the pterygoid flange is modified, being broad and facing posteromedially. Second, the elevated platform that supports the distal ends of the ectopterygoid and palatine bar as noted above is incipiently present in *C. montealtensis*, *C. paulistanus*, *Mariliasuchus*, *Notosuchus*, and *Yacarerani*.

Third, the ventral surface of the pterygoid flanges of most notosuchians is flat and lack parachoanal fossae. Some sphagesaurids, however, have distinct depressions on the ventral surface of the pterygoid. In *C. montealtensis* a subcircular and deep fossa is located posteromedially to the contact with the ectopterygoid and palatine bar (parachoanal fossa *sensu* [10]). In *S. huenei* (RCL-100) a deep and small parachoanal fossa is present but is located much more medially on the ventral surface of the pterygoid flange (close to the choanal groove). The anterior margin of the pterygoid flange of *C. stenognathus* bears a narrow and elongated concavity, but such depression is clearly not as deep and developed as the parachoanal fossa of *S. montealtensis*. Similarly, a shallow concavity is also present in *Mariliasuchus* (MZSP-PV 50, URC R67) but this seems to be absent in other taxa (e.g., *Notosuchus*, *Yacarerani*) in which this region is well preserved. Some baurusuchids and some sebecids (*Lorosuchus*, *Sebecus querejazus*) have similarly located depressions on the ventral surface of the pterygoid flange. The development of parachoanal fossa therefore seems to be a labile character, showing a great deal of variation among sphagesaurids, baurusuchids, and sebecids.

Anterior to the base of the flanges the pterygoid projects a trough-shaped process that extends anteriorly forming the choanal groove that bears a well-developed septum (Figure 21). This trough-shaped process is formed by a delicate lamina that is ventrally concave and poorly preserved in MZSP-PV 139 (but its anterior extension can be seen in lateral view). The anterior end of the choanal groove contacts the dorsal process of the palatine located at the anteroposterior midpoint of the suborbital fenestra, anterior to the choanal region (see above). Within the choanal groove the pterygoid bears a sagittaly oriented choanal septum that has an extremely narrow posterior end (as a thin ridge), differing from the posteriorly broad septum of *C. montealtensis* and *C. paulistanus*. Anteriorly, its ventral surface broadens lateromedially and is concave. This concavity broadens and deepens anteriorly (Figures 21, 13J). This groove is certainly absent in the choanal septum of *C. montealtensis* and also seems to be absent in *C. paulistanus* although the ventral surface of the septum in the holotype is damaged. The anterior end of this septum is hidden below the palatines and cannot be completely observed. The posterior region of the choanal septum of *C. stenognathus* resembles in its morphology the condition of other advanced notosuchians (e.g., *Mariliasuchus*, *Notosuchus*), although the concave ventral surface is not as broad and deep as in *C. stenognathus*. In *Yacarerani*, instead, the septum is extremely narrow along its entire length. The morphology of other sphagesaurids, however, is currently unknown (e.g., *S. huenei*, *Caryonosuchus*, *Armadillosuchus*, *Adamantinasuchus*), but as in other clades of Notosuchia (e.g., Uruguaysuchidae), there seems to be a high variability in the shape of the choanal septum at low taxonomic levels.

The posterior region of the pterygoids extends dorsally to meet the quadrate and basisphenoid. The base of this process bears internal cavities separated by thin bony septa (Figure 13L). Given the long pterygoid process of the quadrate that extend down to the midpoint of the lateral margin of the basisphenoid (see above), the quadrate process of the pterygoid is relatively reduced in their dorsal projection as in other sphagesaurids (*C. montealtensis*, *S. huenei*, *Armadillosuchus*, *Yacarerani*). In *Notosuchus* and other notosuchians (including *Araripesuchus*, *Uruguaysuchus*, *Simosuchus*, *Malawisuchus*, baurusuchids, sebecids, peirosaurids, and mahajangasuchids), this process extends further dorsally and contacts the quadrate close to the location of the lateral Eustachian foramina. The posterior surface of the dorsal process of the pterygoids bears a thin oblique ridge on each side that extends from the posterior margin of the pterygoid flanges, converging dorsally near the anterior end of the basisphenoid (Figure 21B).

#### Mandible

Both mandibular rami are completely preserved in MZSP-PV 139 (Figure 22) and are only slightly distorted. The symphyseal region is narrow and long and the posterior rami diverge from each other up to the level of the external mandibular fenestra and are parallel to each other posterior to this point, as in most advanced notosuchians.

The symphysis is anteroposteriorly long and extends along the first six dentary alveoli (Figure 22). The dentaries form most of the mandibular symphysis and the splenials contribute only to the symphysis along the sixth alveolus in dorsal view and to a smaller region in ventral view (Figure 23). The dentaries progressively taper anteriorly in dorsal and lateral view (Figures 22, 24), but in ventral view, the anterior half of the symphyseal region has parallel-sided lateral margins (Figure 23). This anterior process is present in all advanced notosuchians (*Morrinhosuchus*, *Notosuchus*, *Mariliasuchus*, and all sphagesaurids), although there is variation in the length and width of this distinct anterior process. The proportions of this process in *C. stenognathus* resemble those of *C. montealtensis* and *C. paulistanus*, being three times as long as wide. In *S. huenei*, *Caryonosuchus*, and *Armadillosuchus* this process is only twice as long as broad, and in all other advanced notosuchians (*Mariliasuchus*, *Notosuchus*, *Morrinhosuchus*, and the sphagesaurids *Adamantinasuchus* and *Yacarerani*) this region is almost as broad as long. Baurusuchids and basal notosuchians (e.g., *Araripesuchus*, *Uruguaysuchus*, *Candidodon*, *Malawisuchus*, *Libycosuchus*) have the generalized condition of mesoeucrocodylians, in which the mandibular rami gradually diverge along the symphyseal region. The dorsal surface of the dentaries (and splenial) along mandibular symphysis is extremely narrow, positioning the left and right lower toothrow closely located from each other as in other sphagesaurids (see Dentition).

The ventral surface of the dentaries is ornamented with shallow and irregular grooves that are well separated from each other (Figure 23). Posterior to the anterior process of the mandibular symphysis, the lateral surface of the dentaries is divided in two distinct regions. The ventral region is ornamented and becomes dorsoventrally higher posterior to the symphyseal region (Figure 24). Due to the oblique crushing of the specimen, this region of the left and right dentaries is asymmetric in their orientation and convexity. In the left dentary this ornamented surface is flat vertically oriented, facing laterally. In the right dentary this surface faces lateroventrally and is slightly convex. The dorsal region of the lateral surface of the dentaries is smooth and faces dorsolaterally. Along the alveolar edge the dentary cruves medially and forms a dorsally facing alveolar shelf. Thus, the posterior half of the lower toothrow of *C. stenognathus* is medially inset with respect to the lateral margins of the dentary (Figure 22). The presence of an alveolar shelf and medially inset toothrow is a derived feature present in advanced notosuchians (*Morrinhosuchus*, *Notoscuhus*, *Mariliasuchus*, and sphagesaurids) as well as in *Labidiosuchus*, *Malawisuchus*, and *Pakasuchus*. This shelf, however, is absent in baurusuchids, sebecids, peirosaurids, mahajangasuchids, and basal notosuchians (e.g., *Araripesuchus*, *Uruguaysuchus*, *Anatosuchus*, *Libycosuchus*, *Simosuchus*). Along the buccal margin of the alveoli, the dentary projects small processes that form incipient alveolar septa between the posteriormost five mandibular teeth.

Below the alveolar shelf, the dentaries of *C. stenognathus* have eight neurovascular foramina that form a slightly curved line the runs parallel to the alveolar margin (Figure 24). One or two small foramina are located along the anterior process of the symphysis in the left and right dentary, respectively. Posterior to this point the neurovascular foramina are much larger and closely spaced from each other. These foramina open into a large internal duct that runs longitudinally along the dentary. This duct is triangular in cross section (with the apex pointing ventrally) and is located ventromedially from the neurovascular foramina. The presence of neurovascular foramina on the lateral surface of the dentary is a widespread feature among mesoeucrocodylians, but a linear arrangement of remarkably large foramina (approximately as long as one alveolus) is only present in some sphagesaurids (*C. montealtensis*, *C. paulistanus*, *Yacarerani*). *Adamantinasuchus* has a single, similarly large foramen that is accompanied by smaller foramina. The condition of other sphagesaurids is currently unknown (e.g., *S. huenei*, *Caryonosuchus*, *Armadillosuchus*). *Mariliasuchus* also has a line of neurovascular foramina but there are only four foramina and they are smaller than in sphagesaurids.

Posterior to the toothrow, the dentaries broaden dorsoventrally and have an overlapping contact with the surangular and the angular. The contact with the angular runs posterolaterally on the ventral surface of the mandibular ramus (Figure 22) and directs dorsally on its lateral surface, at the level of the anterior margin of the external mandibular fenestra (Figure 23). Thus, as in most notosuchians, the dentary fails to project posteriorly along the ventral margin of the external mandibular fenestra [27]. The dentary forms the curved dorsal half of the anterior margin of the external mandibular fenestra, which is best preserved on the right side of MZSP-PV 139.

The posterodorsal branch of the dentary is divided in two distinct processes, a ventral process exposed on the lateral surface of the lower jaw and a dorsal process that fits into a large notch between the medial and lateral rami of the bifurcated anterior end of the surangular, as noted for *C. montealtensis* by Andrade & Bertini [10]. The ventral process extends along the dorsal margin of the external mandibular fenestra up to the anteroposterior midpoint of this opening and is separated from the dorsal process by a deep and broad U-shaped concavity that faces posteriorly (Figure 24). The dorsal process overlaps the surangular along the dorsal surface of the mandibular ramus and extends posteriorly up to the level of the coronoid tuberosities of the surangular (Figures 22, 24). The dentary-surangular suture extends anteriorly from the posterior tip of the dorsal process along the dorsal margin of the medial surface of the mandibular ramus. This suture meets the splenial at the posterior corner of the last mandibular alveolus. This kind of sutural contact between the dentary and surangular is also present in *Mariliasuchus* and other sphagesaurids (*Adamantinasuchus*, *Yacarerani*, *C. paulistanus*, *C. montealtensis*), although in most of these taxa the dorsal and ventral processes of the posterodorsal branch of the dentary are separated by a narrow and elongated V-shaped facet that receives the surangular external ramus (*sensu* [10]). An interlocking sutural contact between the dentary and the surangular, however, is not unique of these forms and some peirosaurids (e.g., *Montealtosuchus*, *Uberabasuchus*, *Lomasuchus*) also have a similar morphology [56].

The splenial covers the entire medial surface of the mandibular ramus up to level of the anterior margin of the mandibular fenestra (Figures 23, 25). The medial surface of the splenial is flat and bears a relatively large and rounded foramen intermandibularis oralis located at the level of the posterior mandibular alveoli and close to the ventral margin of the splenial (Figure 25). The presence of the anterior opening located at the level of the posterior teeth is also found in advanced notosuchians, baurusuchids, and sebecids, contrasting with the condition of most basal notosuchians (e.g., *Araripesuchus*, *Uruguaysuchus*, *Anatosuchus*, *Simosuchus*) in which the foramen lies just posteriorly/laterally to the splenial-splenial suture at the mandibular symphysis. The foramen intramandibularis oralis of *C. stanognathus* is not as large and elongated (slot-like) as in baurusuchids and sebecids [57] but resemble in shape and size those of advanced notosuchians (*C. paulistanus*, *Yacarerani*, *Notosuchus*, *Mariliasuchus*). The rounded foramen intermandibularis oralis, however, is slightly more dorsally placed in *Mariliasuchus* and *Notosuchus* than in *C. paulistanus* and *C. stenognathus*.

The ventral margin of the splenial contacts the dentary along the ventromedial edge of the post-symphyseal region of the mandibular ramus. Therefore, the splenial has an extremely limited participation on the ventral surface of the mandible, as in *Mariliasuchus* and other sphagesaurids (*C. paulistanus*, *C. montealtensis*, *Yacarerani*). The splenial ventral margin gradually curves dorsally overlapping the medial surface of the anterior branch of the angular, reaching the anteroventral corner of the mandibular adductor fossa (Figure 25).

The posterior margin of the splenial is smoothly concave, forming the entire anterior edge of the mandibular adductor fossa and contacts the surangular internal ramus (*sensu* [10]) at the anterodorsal corner of this fossa. The imperforated splenial-angular suture of these mesoeucrocodyians contrasts with the condition of extant crocodylians and basal crocodyliforms (e.g., *Protosuchus richardsoni*, *Hsisosuchus chowi*), in which the posterior margin of the splenial forms the anterior margin of the foramen intermandibularis caudalis that is located at the splenial-angular suture, through which exits the ramus intermandibularis caudalis of the mandibular nerve (V_3_). The morphology of the posterior margin of the splenial of *C. stenognathus* resembles that of other advanced notosuchians (e.g., *Notosuchus*, *Mariliasuchus*, *C. paulistanus*, *Yacarerani*), but also that of baurusuchids (*B. salgadoensis*), sebecids (*Bretesuchus*), peirosaurids (*Montealtosuchus*), and *A. gomesii*. The absence of a distinct foramen intermandibularis caudalis in notosuchians suggests this nerve exited from the anterior margin of the mandibular aductor fossa. The concavity of the posterior margin of the splenial was identified as the foramen intermandibularis caudalis by Iori & Carvalho [11] in *C. paulistanus* but has been mistakenly identified by Osi [3] in *Yacarerani* (in which the foramen intermandibularis oralis was labeled as the foramen intermandibularis caudalis). Dorsal to the foramen intermandibularis caudalis, the posterior margin of the splenial of extant crocodylians has a large notch that is overlapped by the coronoid, a bone that has not been preserved in any advanced notosuchian (see below).

Anterior to the splenial-surangular, the dorsal margin of the splenial forms the entire medial margin of the mandibular alveoli (Figure 22). The splenial projects small triangular processes between each tooth that form incipient alveolar septa that do not meet the similar projections that the dentary has along the buccal alveolar edge, as in most notosuchians [57], [58]. The dorsal (alveolar) margin of the splenial of *C. stenognathus* is a lateromedially thin lamina, instead of being lateromedially broad as in some baurusuchids and sebecids [57].

As noted above, the splenials meet at the mandibular symphysis at the level of the sixth mandibular tooth (Figure 22). The splenial-splenial suture extends briefly on the dorsal surface of the symphysis up the anterior margin of the sixth mandibular tooth. The anterior end of the splenial is sutured to the dentary on the dorsal surface of the mandible along a transversely oriented suture that extends between the fifth and sixth mandibular tooth. The posterior surface of the mandibular symphysis is dorsoventrally deep and has the splenial-splenial suture running along the sagittal axis. This surface is oriented vertically and faces posteriorly instead of facing posterodorsally as in most other notosuchians (*Araripesuchus*, *Anatosuchus*, *Mariliasuchus*). A small and blunt posterior splenial peg is present close to the ventral end of the posterior surface of the mandibular symphysis (Figure 25). The presence of a posterior splenial peg is a widespread feature among notosuchians. However, several basal notosuchians (e.g., *Uruguaysuchus*, *A. buitreraensis*, *A. tsangatsangana*), *Morrinhosuchus*, and *Notosuchus* have a large peg that is located on the ventral surface of the mandibular symphysis. *Caipirasuchus stenognathus* shares with other sphagesaurids (*C. paulistanus*, *C. montealtensis*, *Yacarerani*), *Mariliasuchus*, *Comahuesuchus*, and baurusuchids the presence of a smaller peg located on the posterior surface of the mandibular symphysis.

The ventral exposure of the splenials along the symphyseal region has a broad triangular shape with the apex pointing anteriorly and the splenial-dentary suture projecting posterolaterally from this point (Figure 23). The participation of the splenial on the ventral surface of the mandibular symphysis is small and extends for only 10 mm, which represents approximately 10% of the symphyseal length (Figure 23). A reduced participation of the splenial on the ventral symphyseal surface is also present in other advanced notosuchians (e.g., *Notosuchus*, *Morrinhosuchus*, *Mariliasuchus*, sphagesaurids) but contrasts with the extensive V-shaped projection of the splenials in more basal notosuchians (e.g., *Malawisuchus*, *Pakasuchus*, *Candidodon*, *Araripesuchus*, *Uruguaysuchus*) and peirosaurids.

As in most mesoeucrocodylians the angular forms the ventral region of the posterior mandibular ramus and is U-shaped in cross section (Figure 24). Its lateral surface forms the entire ventral margin of the subcircular external mandibular fenestra. The lateral surface of the angular has a shallow fossa that extends below the external mandibular fenestra. The ventral margin of this fossa is a laterally projected ridge located close to the ventral margin of the lateral surface of the angular (Figure 24). This region is not preserved in other sphagesaurids, except for *Yacarerani* and *Adamantinasuchus* that have a similar ridge and smooth elongated fossa along the ventral margin of the external mandibular fenestra. This fossa is also present in other advanced notosuchians (*Notosuchus*, *Mariliasuchus*) but is absent in basal notosuchians (*Araripesuchus*, *Uruguaysuchus*, *Libycosuchus*, *Simosuchus*), baurusuchids, sebecids, peirosaurids, and mahajangasuchids. It must be noted that this fossa and its ventral ridge is restricted to the ventral margin of the external mandibular fenestra, and is not considered homologous to the more posteriorly located angular ridge present in many crocodyliforms (including basal notosuchians and baurusuchids) which forms the dorsal limit of the depression of the angular that serves for the insertion of the m. pterygoideus ventralis [45]. In *C. stenognathus* (as in other advanced notosuchians) the fossa and ridge of the angular becomes less pronounced and disappears posteriorly to the external mandibular fenestra. The lateral surface of the angular overlaps the lateral surface of the surangular and increases in dorsoventral height posterior to the external mandibular fenestra, from the posteroventral corner of the external mandibular fenestra to the midheight of the mandibular ramus. Posteriorly, the angular-surangular suture is straight and extends up to the caudal end of the retroarticular process.

The medial lamina of the angular has a dorsally directed process that has a rugose and dorsally blunt apex (Figure 26). This process resembles the broad process of extant crocodylians located posteriorly to the foramen intermandibularis caudalis (medial projection of the medial border of the adductor fossa [50]). The dorsal process of the medial lamina of the angular of extant forms also has a rugose surface, produced by the aponeurotic attachment of the basal part of the mandibular adductor tendon [59] (coronar or coronoid aponeurosis *sensu* [50], [60]). Given that this site of tendinous attachment is located posteriorly to the exit of the ramus intermandibularis caudalis of the mandibular nerve (V_3_), we interpret that in MZSP-PV 139 this ramus exits directly from the mandibular adductor fossa (see above). The rugose dorsal process of the medial lamina of the angular seems to be absent (e.g., *Simosuchus*) or less developed (*Yacarerani*, *Mariliasuchus*, *A. gomesii*) in other notosuchians. The medial lamina of the angular extends anteroventrally from this point, wedging between the splenial and the dentary on the ventromedial surface of the mandibular ramus (Figure 25). The posterior half of the medial lamina of the angular overlaps ventrally the entire articular bone.

The surangular forms the dorsal region of the posterior mandibular ramus (Figure 24). As in most advanced notosuchians, the dorsal surface of the surangular is arched above and posteriorly to the external mandibular fenestra [27]. The anterior ramus has an external branch that fits into the U-shaped concavity of the posterodorsal branch of the dentary and an internal branch that overlaps the medial surface of the dentary, as described for *C. montealtensis* [10]. This morphology is also present in other sphagesaurids (*C. paulistanus*, *Adamantinasuchus*, *Yacarerani*), *Mariliasuchus*, *Labidiosuchus*, peirosaurids (*Lomasuchus*, *Uberabasuchus*, *Montealtosuchus*), and some neosuchians (e.g., *Sarcosuchus*), but is absent in baurusuchids, more basal notosuchians (*A. gomesii*, *Uruguaysuchus*, *Simosuchus*), and *Kaprosuchus*.

The left external surangular branch bears a large anterior surangular foramen on its dorsal surface (Figure 24), whereas the right element has two anterior surangular foramina. The preserved surangular of *C. montealtensis* also has two similar positioned foramina but the type specimen of *C. paulistanus* seems to lack a large foramen in the anterior end of the external surangular branch. Posterior to this foramen the dorsal surface of the surangular is flat, more so that in extant crocodylians, which have a flattened surface where the m. adductor mandibulae externus superficialis inserts [50].

The medial surface of the internal surangular branch bears two elongated protuberances separated by a longitudinal trough-shaped surface (Figure 26). These are referred here as the dorsal and ventral coronoid tuberosities of the surangular, as similar processes enclose the posterodorsal process of the coronoid in extant crocodylians (*Caiman latirostris* FMNH 9713, *Paleosuchus trigonatus* FMNH 69882; *Tomistoma schlegelii* FMNH 206755). This region of the surangular is also the attachment site of the lateral surface of the mandibular adductor tendon in extant taxa [59] (fibrous cushion of coronar aponeurosis *sensu* [50], [60]) and cartilago transiliens. The dorsal coronoid tuberosity of the surangular has a rugose medial surface and extends longitudinally along the medial margin of the dorsal process of the posterodorsal branch of the dentary (Figure 26). The ventral coronoid tuberosity of the surangular is dorsoventrally low and extends longitudinally along the ventral margin of medial surface of the anterior branch of the surangular (Figure 26), projecting moderately into the mandibular adductor fossa. These rugose coronoid tuberosities of the surangular (divided by a longitudinal cocavity) are also found in other advanced notosuchians (e.g., *C. montealtensis*, *C. paulistanus*, *Yacararani*, *Mariliasuchus)*. Baurusuchids and basal notosuchians (*A. gomesii*, *A. tsangatsangana*, *Simosuchus*) have less prominent tuberosities and only a slightly concave surface between them.

The surangular is dorsoventrally low above the external mandibular fenestra and expand posteriorly forming the dorsal half of the caudal margin of this opening (Figure 24), The dorsal margin of the surangular is ventrally deflected along the articular region of the mandible and extends posteriorly up to the distal end of the retroarticular process (Figure 27C). The surangular-articular suture is located on the medial surface of the mandibular ramus and forms the lateral margin of the glenoid facet for the lateral quadrate condyle (Figure 27B). The medial surface of the surangular bears a single foramen above its suture with the articular and posterior to the external mandibular fenestra (Figure 27A).

The coronoid is absent in MZSP-PV 139. As described above the medial surface of the surangular of *C. stenognathus* bears two rugose tuberosities (Figure 26) that resemble the articular facet for the posterodorsal branch of the coronoid of extant crocodylians. A similar morphology is present in at least three complete mandibles of *Mariliasuchus* (MZSP-PV 50, MZSP-PV 51, MN 6756-V), all well preserved specimens of *Notosuchus*, and the type specimens of *C. paulistanus*, *C. montealtensis*, and *Yacarerani*. Additionally, no coronoid bones have been found in other well-preserved basal mesoeucrocodylians, such as several well-preserved mandibles of baurusuchids, peirosaurids (e.g., *Montealtosuchus*), and basal notosuchians (*A. gomesii*, *Simosuchus*). An elongated fragmentary bone was identified as a possible coronoid in *A. tsangatsangana* [47], but this fragment also resembles morphology of the posterodorsal process of the splenial so its identity as a coronoid cannot be confirmed. The absence of a separate coronoid bone in multiple well-preserved and complete lower jaws of different notosuchians lends support to the interpretation that the coronoid was indeed absent as a separate ossified element in these basal mesoeucrocodylians. This could be explained by a complete fusion of the coronoid to the surangular (as suggested by Zaher *et al.* [17] for *Mariliasuchus*), or to the splenial, or simply by the absence of a coronoid ossification. In fact, some well-preserved non-notosuchian mesoeucrocodylians also lack a separate ossified coronoid (e.g., *Shamosuchus*) so that this feature may represent a more widespread feature among non-eusuchian mesoeucrocodylians.

Both articulars have been completely preserved in MZSP-PV 139, although the right retroarticular process is slightly damaged. The anterior process of the articular has a narrow triangular shape in dorsal view (Figure 27B) and extends along the medial surface of the posteroventral margin of the external mandibular fenestra. Its dorsal surface is slightly concave and is medially bounded by a rugose crest that projects anteroventrally from the anterior edge of the glenoid crest (Figures 27A–B). This crest serves as the attachment site of the m. adductor mandibulae posterior [45] in extant crocodylians. As in all mesoeucrocodylians [27], there are no signs of a prearticular ossification in *C. stenognathus*.

The glenoid region of the articular has two distinct facets for the medial and lateral condyles of the quadrate separated by a broad and dorsally convex longitudinal crest (Figure 27). The articular facets are anteroposteriorly long and lateromedially narrow, being approximately twice as large as the articular surface of the quadrate condyles. Similarly elongated facets are also found in *Mariliasuchus*, *Notosuchus*, *Yacarerani*, *Malawisuchus*, and *Pakasuchus*, which were interpreted as evidence supporting anteroposterior motion of the jaw during occlusion [3], [4], [14], [19], [61]. This condition was probably present in other advanced notosuchians (*C. paulistanus*, *Caryonosuchus*, *S. huenei*, *Armadillosuchus*) that have extensive wear facets that suggest fore-aft motion of the jaws [3], [5]–[7], [11], [12]. Baurusuchids, sebecids, peirosaurids, mahajangasuchids, and basal notosuchians (e.g., *Araripesuchus*), instead, have articular facets that are at the most 50% longer than the articular surface of the quadrate condyles.

The medial facet extends ventromedially from the broad longitudinal crest, so that its articular surface faces dorsomedially (Figures 27A, B, E), as in most basal mesoeucrocodylians. The inner margin of the medial articular facet is limited by a slightly developed longitudinal ridge (Figure 27E). This ridge gradually curves laterally along its posterior region forming the posterior limit of the articular facet. The articular facet for the lateral quadrate condyle faces dorsolaterally. The medial and lateral articular facets lack an elevated posterior buttress, a derived feature shared by several notosuchians (e.g., *Araripesuchus*, *Uruguaysuchus*, *Simosuchus*, *Malawisuchus*, *Pakasuchus*, *Notosuchus*, *Mariliasuchus*, *Yacarerani*). Other notosuchians, instead, have the ridge that posteriorly borders the articular facets for the quadrate, (e.g., *Libycosuchus*, sebecids, peirosaurids, mahajangasuchids, baurusuchids; see below). The posterior edge of the medial facet is separated from the medial flange of the retroarticular process by a distinct step (Figures 27A, B, E) but the lateral facet is continuous with the dorsal surface of the lateral flange of the retroarticular process (Figures 27B, E). The continuous dorsal surface of the lateral glenoid facet and the retroarticular process is a feature shared only with some notosuchians (*Simosuchus*, *Mariliasuchus*, *Notosuchus*, *Yacarerani*), although the condition of many taxa is still unknown (e.g., almost all sphagesaurids). More basal notosuchians have a distinct step that separates the lateral glenoid facet from a ventrally recessed lateral flange of the retroarticular process (e.g., *Araripesuchus*, *Anatosuchus*, *Uruguaysuchus*, peirosaurids, mahajangasuchids, sebecids). Baurusuchids have a prominent posterior crest that limits the posterior extent of the lateral glenoid facet.

The retroarticular process of *C. stenognathus* has short lateral flange that extends posteriorly from the lateral glenoid facet (Figure 27B) and a large paddle-like medial flange (Figure 27). The lateral flange is triangular shaped with its apex pointing posteriorly. This surface is shorter than the width of the glenoid facets for the quadrate condyles, as in basal crocodyliforms and some notosuchians (*Libycosuchus*, *Simosuchus*, *Pakasuchus*, *Notosuchus*, *Mariliasuchus*, *Yacarerani*, *B. salgadoensis*). Neosuchians have a much longer (and ventrally recessed) lateral flange of the retroarticular process, being equal or longer than the lateromedial width of the articular surface of the quadrate condyles. This condition is also present in basal notosuchians (*Araripesuchus*, *Anatosuchus*), peirosaurids, and sebecids [62], in which the lateral flange is also formed by a posterior extension of the surangular. The dorsal surface of the lateral flange of the retroarticular process of MZSP-PV 139 is slightly concave and its posterior apex slightly elevates dorsally as a small rounded bulge with rugose surface located at the posterolateral corner of the retroarticular process (Figures 27B, D, E), as in all mesoeucrocodylians but not basal crocodyliforms. This rugose bulge is interpreted as homologous to a similar protuberance found in extant crocodyliforms that mark the posteriormost limit of insertion of the m. depressor mandibulae [59], [63]. The lateral flange of the retroarticular process of MZSP-PV 139 is limited along its medial margin by a faintly developed and oblique crest that extends posterolaterally from the posterior end of the broad ridge (that separates the lateral and medial glenoid facets of the articular) to the rugose bulge at posterior end of the retroarticular process (Figure 27). This crest forms the limit between the lateral and medial flanges of the retroarticular process. Advanced notosuchians (e.g., *Marilasuchus*, *Notosuchus*, *Yacarerani*), *Pakasuchus*, *Simosuchus*, and baurusuchids share with *C. stenognathus* the posterolateral orientation of ridge (oriented at approximately 45 degrees with the longitudinal axis of the mandibular ramus). However, all other crocodyliforms have this ridge oriented longitudinally along the retroarticular process (including *Araripesuchus*, *Anatosuchus*, *Libycosuchus*, peirosaurids, mahajangasuchids, and sebecids). In some neosuchians (including some extant taxa) this ridge is markedly developed as a prominent crest on the dorsal surface of the retroarticular process that divides its lateral and medial flanges.

The medial flange of the retroarticular process is much more extensive than the lateral flange and forms a slightly concave paddle-shaped lamina that is obliquely oriented with respect to the sagittal plane of the skull, facing posteromedially and slightly dorsally (Figure 27). The medial flange is therefore strongly deflected medially with respect to the lateral flange, a derived feature shared by all known notosuchians (including peirosaurids, mahajangasuchids, sebecids, and baurusuchids). This similarity was early noted by Busbey [62] while comparing the medially deflected medial flange of *Sebecus* cf. *huliensis* and notosuchians. The posterior margin of the medial flange runs ventromedially from the posterior rugose bulge of the retroarticular process and then turns anteriorly, forming an extensive arch and ending at the anteroposterior level of the posterior margin of the glenoid facet (see Figure 27). This rounded and extensive margin creates the paddle-shaped morphology of the medial flange of the retroarticular process, a feature also found in *Libycosuchus*, *Simosuchus*, *Pakasuchus*, baurusuchids, and advanced notosuchians (*Notosuchus*, *Mariliasuchus*, *Yacarerani*, *C. paulistanus*).

The anteroventral end of the medial retroarticular flange is a distinct process of the medial flange that is more vertically oriented than the posterior surface and faces more medially (Figure 27A, E). This anterior process of the medial retroarticular flange is not markedly separated from the rest of the medial flange in *C. stenognathus* but has a distinct orientation. The anterior process is dorsoventrally lower than the rest of the medial flange and extends anteriorly bordering the medial glenoid facet of the articular (Figure 27A). The anterior end of this process is located at the anteroposterior midpoint of the medial glenoid facet. The dorsal margin of this anterior projection of the medial retroarticular flange is ventrally recessed from the posterior margin of the medial glenoid facet (Figure 27E) and the ventral margin is sinuous, being concave posteriorly and convex anteriorly (Figure 27A). A similar anterior process of the medial flange is absent in *Notosuchus* and most notosuchians, but is present in *Mariliasuchus* and *Yacarerani*. This region, however, is currently unknown in all other sphagesaurids (e.g., *C. montealtensis*, *C. paulistanus*, *S. huenei*, *Armadillosuchus*, *Caryonosuchus*, *Adamantinasuchus*). Baurusuchids also have a distinct anterior process of the medial flange but is different from the condition found in *Caipirasuchus stenognathus*, *Mariliasuchus*, and *Yacarerani*, being a robust, deep process that projects anteroventrally as a pendant process [29].

### Dentition

The dentition of MZSP-PV 139 is one of the most important anatomical features preserved in this specimen, due to its completeness, quality of preservation, and the combination of derived characters present in advanced notosuchians (sphagesaurids, *Mariliasuchus*, *Notosuchus*). Although the dentition of sphagesaurids bears numerous unique features, most of these taxa are known by specimens with incompletely or poorly preserved toothrows (e.g., *Caryonosuchus*, *S. huenei*, *Armadilosuchus*). The only sphagesaurid known so far with well preserved dentition is *Yacarerani*, although this taxon has multiple autapomorphic features that contrast with the condition of other sphagesaurids. MZSP-PV 139 therefore adds significant new information that increases our knowledge on the dental characters of sphagesaurids.

#### General features

The dentition of *C. stenognathus* shares with advanced notosuchians the presence of three main crown morphologies: incisiviform, caniniform, and molariform (following [61]).

The upper toothrow is composed of two incisiviforms, a caniniform, a “transitional” tooth, and six molariforms. The two incisiviforms are closely spaced to each other in the anterior region of the premaxilla and are slightly displaced form their natural position (Figure 28A). The presence of two premaxillary incisiviforms is shared by most advanced notosuchians (including *C. paulistanus*), except for *Notosuchus* that has three and *S. huenei* that lacks incisiviforms in the upper toothrow. The condition of *C. montealtensis* is unknown given the precaniniform region has not been preserved in the holotype of this taxon. In both *C. paulistanus* and *C. stenognathus* the premaxillary incisiviforms are not as procumbent as the anterior premaxillary teeth of *Mariliasuchus*, *Yacarerani*, and *Adamantinasuchus*. The caniniform is located in the premaxilla (Figure 28A) and is followed by a transitional tooth that is set at the premaxilla-maxilla contact, with the maxilla forming the posterior margin of the alveolar edge, another feature shared by most advanced notosuchians (*Notosuchus*, *Mariliasuchus*, *C. montealtensis*, *C. paulistanus*, *Yacarerani*), except for *S. huenei* and *Caryonosuchus*. The six maxillary molariforms are subequal in size, except for the posteriormost element that is slightly smaller. This seems to be the widespread condition among advanced notosuchians, except for *Mariliasuchus* that have only five maxillary teeth (not counting the small tooth located at the premaxilla-maxilla suture in the latter taxon).

The entire premaxillary toothrow of *C. stenognathus* is set in a continuous alveolar groove that ends in a complete bony septum located distal to the transitional tooth, separating the premaxillary alveolar groove from the maxillary alveolar groove that lodges the molariforms. In these two grooves the alveoli are delimited by incipient bony projections of the premaxilla and maxilla. A similar condition is found in the other species of *Caipirasuchus* and *Yacarerani*, although in the latter taxon a large diastema separates the anterior (premaxillary) alveolar groove from the posterior (maxillary) alveolar groove. In contrast, complete septa separate all alveoli in *S. huenei*, *Armadillosuchus*, *Comahuesuchus*, and baurusuchids. In *Notosuchus* and *Mariliasuchus* the entire upper toothrow is set in a continuous alveolar groove.

The lower toothrow is composed of three anterior incisiviforms, a transitional tooth, and six posterior molariforms. The lower incisiviforms are only slightly procumbent and gradually increase in size posteriorly (Figure 28B), as in *Notosuchus*, *C. montealtensis*, *C. paulistanus*, *S. huenei*, and *Armadillosuchus*. This contrasts with the remarkably large and highly procumbent anterior incisiviform of *Yacarerani* and *Adamantinasuchus*, as well as with the two highly procumbent but less developed incisiviforms of *Mariliasuchus*. Given the long and narrow mandibular symphysis of *C. stenognathus*, the left and right lower toothrows are parallel and closely spaced from each other, from the first incisiviform to the first molariform tooth (Figure 22). This feature is only known in sphagesaurids (*C. paulistanus*, *C. montealtensis*, *S. huenei*, *Armadillosuchus*, *Caryonosuchus*, and *Yacarerani*), as well as in the fragmentary taxon *Labidiosuchus*. The closely spaced left and right symphyseal toothrows of sphagesaurids differs from the well-separated anterior region of the left and right lower toothrows of *Notosuchus*, *Morrinhosuchus*, and *Mariliasuchus* (as well as those of more basal notosuchians).

The lower incisiviforms are set in a continuous alveolar groove that is separated by a complete bony septum from the alveolus of the transitional tooth. This lower incisiviform alveolar groove is shared with *C. paulistanus* and *Yacarerani*, but seems to be absent in other sphagesaurids (e.g., *S. huenei*), as well as other notosuchians (including *Notosuchus* and *Mariliasuchus*). Posteriorly, the first lower molariform is also set in a distinct alveolus that has broad bony septa that separates it from the preceding and subsequent alveoli. This separation of the first lower molariform from the rest of the toothrow is also present in *C. paulistanus* [11], although in this taxon the separation is larger than in MZSP-PV 139 and was recognized as a diastema by Iori & Carvalho [11]. The posterior five lower molariforms are set in a continuous alveolar groove. The condition of *C. montealtesis* seems to be similar to the above-described condition, at least in the separation of the first molariform from the rest of the toothrow. *Yacarerani* shares with *Caipirasuchus* the presence of an anterior alveolar groove for the incisiviforms and a posterior groove for the molariform teeth, although these two grooves are separated in *Yacarerani* by a large diastema and in *Caipirasuchus* by two small diastemata that enclose the first molariform tooth. The presence of a diastema may characterize sphagesaurids, but at the moment complete mandibles of other taxa are currently not known. *Notosuchus*, *Morrinhosuchus*, *Mariliasuchus*, and more basal notosuchians have the first four alveoli separated by complete bony septa and the lower molariforms set in a continuous alveolar groove but there is no clear separation between the anterior and posterior regions of the lower dentition.

#### Incisiviforms

In MZSP-PV 139 the incisiviform teeth are conical elements with a slightly bulbous crown base and a pointed apex (Figure 28). These teeth have a barely developed constriction between the root and the crown (or completely lack this constriction in some cases). The crown-root limit is marked by the abrupt end of the thick coat of enamel. The basal bulbous region of the tooth occupies approximately one third of the crown. Then, the crown tapers rapidly toward apex, having a convex mesial margin and a concave distal margin. Thus, the tips of the incisiviform teeth are posteriorly recurved. The enamel surface of these elements bears well-developed apico-basal ridges (Figure 28), resembling the condition of *C. paulistanus*, *S. huenei*, *Armardillosuchus*, and *Caryonosuchus*. *Mariliasuchus* also has apicobasal keels in the incisiviforms, but much more faintly developed than in *C. stenognathus* and the above-mentioned taxa. The incisiviforms of *Notosuchus*, *Yacarerani*, *Adamantinasuchus*, and all other notosuchians lack this derived feature. These ridges are closely spaced to each other and extend over the buccal and lingual surfaces of the crown, varying between 6 and 8 on the buccal side and 7 to 9 on the lingual side. The enamel surface of anterior incisiviforms is smooth except for the above-mentioned ridges.

#### Caniniform

A single caniniform is present posteriorly on the premaxilla and this type of tooth is absent in the lower toothrow, as in most advanced notosuchians. As in *Notosuchus* [61], the caniniform is more than twice the size of the preceding teeth, has a conical shape and a poorly expanded base of the crown (Figure 28A). The apex is recurved posteriorly and the surface of the enamel bears apicobasal ridges as in *Mariliasuchus*, but differing from the smooth enamel surface of the caniniform of *Notosuchus*. The distal margin of the caniniform of *C. stenognathus* bears a slightly developed keel with small rounded denticles.

#### Transitional tooth

A “transitional” tooth is present in the alveolus located at the premaxilla-maxilla suture and in the fourth dentary alveolus (Figure 29). This element is larger than the incisiviforms but smaller than the molariforms. Furthermore, its crown resembles that of incisiviforms in being conical but differs from them in the presence of a distal keel that bears small tuberous denticles. The denticles and keel are restricted to the mid-height of the crown and are well separated from the apex of the crown (as it occurs in the transitional tooth of *Notosuchus*). Interestingly, the keel of the upper and lower transitional tooth is located on the distal margin (Figure 29), as opposed to the mesially located keel of lower molariforms (see below). In contrast to the molariforms the upper and lower transitional teeth probably did not occlude with each other given they lack the extensive wear facets that are present in molariform teeth (see below).

#### Molariforms

The molariform teeth of MZSP-PV 139 are present in the posterior section of the lower and upper toothrow. The tooth crown shape and orientation markedly differs from the condition present in the incisiviforms. The crown is separated from the root by a well-developed constriction, and the base of the crown is much more bulbous than those of the incisiviform teeth. The molariform crowns are tear-drop shaped in cross section and are oriented obliquely to the longitudinal axis of the skull (Figures 12, 22). The acute end of the tear-drop shaped crown corresponds to the distal keel of the maxillary teeth (Figure 12) and the mesial keel of the posterior dentary teeth (Figure 22). Thus, the molariform upper and lower toothrows are inversely oriented as opposing triangles. The oblique orientation of the molariform teeth, and their inverse orientation between the upper and lower toothrows is a unique feature shared by advanced notosuchians (*Notosuchus*, *S. huenei*, *C. montealtensis*, *C. paulistanus*, *Adamantinasuchus*, *Yacarerani*), contrasting with the plesiomorphic longitudinally oriented teeth of other notosuchians (including *Morrinhosuchus*). As noted by Andrade & Bertini [64], the available specimens of *Mariliasuchus* show some degree of variability in this feature, some have a longitudinally oriented carina (MZSP-PV 50, 51) whereas others have an obliquely oriented carina (URC R68, UFRJ 106R). In unworn teeth, the keel of the molariform elements runs from the tooth apex to the base of the crown and is composed by a series of denticles extending along a smooth arch (Figures 30A–C). This slightly curved keel is slightly buccally concave in the upper dentition (Figure 30A) and lingually concave in the lower teeth. The keel of *C. stenognathus* lacks the accessory row of denticled keels that characterize the molariform teeth of *Yacarerani* [15] and that is incipiently developed in *Adamantinasuchus*.

The four most apical denticles are extremely small (extending along 1.64 mm), closely spaced, and seem to be formed mainly by a thickened layer of enamel, the surface of which is smooth (Figures 30B–C, G). The small size of these first denticles markedly contrasts with the large tooth apex, making a clear distinction between the tooth apex and the denticled keel, as in other notosuchian crocodyliforms (e.g., *Mariliasuchus*, *S. huenei*, *Armadillosuchus*). The central denticles of the keel are the largest (Figures 30C, E, G), being approximately twice the buccolingual width and height of the apical and basal denticles, a condition also present in *Mariliasuchus* and other sphagesaurids (*Yacarerani*, *Adamantinasuchus*, *Armadillosuchus*). The denticles gradually decrease in size towards the base of the crown (Figures 30D–G), so that the basalmost denticles are smaller than the central denticles (but larger than the apical ones).

All denticles are buccolingually broad, globular shaped, and end in a broad rounded apex (Figures 30E–F). These denticles are formed by constrictions of the enamel and dentine as the denticles of ziphodont crocodyliforms (*sensu* [65]) but different from ziphodont teeth in several aspects. Firstly, the denticles of MZSP-PV 139 are cup-shaped ending in a broad apex, whereas the ziphodont denticles are buccolingually narrow structures that meet at the mesial or distal margin forming a sharp and apicobasally extensive edge. Secondly, the height of the diaphysis between denticles is very short (less than 50% the height of the adjacent denticles) and the cella is broad and deep because of the globular morphology of each denticle (Figure 30E). In constrast, cellae are much narrower in ziphodont crocodyliforms and the diaphyses are usually much higher (approximately 80% the denticle height in ziphodont crocodyliforms except for those of *Dakosaurus andiniensis* [66]). Finally, ziphodont crocodyliforms have relatively minor size variation in most denticles whereas the denticles of *C. stenognathus* vary greatly in size along the apicobasal keel (Figures 30B, E, G). The morphology of the denticles of *C. stenognathus* resemble in these features that of *Mariliasuchus*, for which Andrade & Bertini [64] coined the term anisomorph. Another derived feature of the denticles of *C. stenognathus* is the presence of a thin enamel ridge (loph) that connects adjacent denticles (Figure 30E). This loph is present also in *Yacarerani* but is absent in *Notosuchus*, *Mariliasuchus* and more basal notosuchians.

The morphology described for the denticled keel of *C. stenognathus* and other notosuchians contrasts, however, with the highly autapomorphic dentition of *Chimaerasuchus paradoxus* [67]. In this taxon, the molariform tooth crowns has three parallel and identical keels that bears tuberosities that could be described either as true cusps or as extremely high tuberous denticles. The distinction between the cusps and tuberous denticles is somewhat arbitrary and boils down to how prominent and separated they are from their adjacent cusps/denticles. The anteriormost (mesial) cusp of each of the three keels can be postulated as homologous to the apex of the crown of other crocodyliforms (including notosuchians) and is much larger than the subsequent cusps or denticles. The apex of the molariforms of *Chimaerasuchus* is separated from the subsequent cusp by a remarkably deep slit. The second cusp (the one located basally to the tooth apex) is almost as prominent as the apex and is also separated by a deep slit from the distal cusps. These slits reach the apicobasal midpoint of the crown and therefore, the apex and the following cusp are well-separated individual structures rather than small tuberosities located along a keel. Distal to this point, the molariform of *Chimaerasuchus* has four tuberosities that gradually decrease in height. These are much smaller than the apex and the first cusp and they are separated by low and narrow slits, resembling more the morphology of the tuberous denticles of notosuchians. In this regard, the morphology of the cusps/denticles of the molariform of *Chimaerasuchus* is highly autapomorphic and only resembles those of the South American taxa in the distal region of the keel. Furthermore, *Chimaerasuchus* has three cuspidated/denticled keels that are parallel to each other and are oriented longintudinally rather than a single denticles keel oriented at 45 degrees respect to the longitudinal axis of the skull (*Notosuchus*, *S. huenei*, *Armadillosuchus*, *Adamantinasuchus*).

*Yacarerani* has a unique condition among notosuchians in having the posteromedially oriented keel that bears tuberous denticles bounded by two additional denticled keels (located buccally and lingually from the central keel). These accessory keels are less developed than the central keel and their tuberous denticles are also smaller. *Adamantinasuchus* has these features incipiently developed, with faintly marked buccal and lingual keels bearing small tuberosities. In this regard, *Chimaerasuchus* and *Yacarerani* share the derived feature of having three keels with tuberous denticles or cusps. Their condition, however, is not identical given that in *Yacarerani* the buccal and lingual keels originate from the central keel (at the single apex of the tooth), diverge basally along the crown, and then converge with the central keel at the base of the crown (see [15]: figure 3F). In *Chimaerasuchus*, instead, the three keels are equally developed and parallel to each other. Most importantly, the three keels of *Chimaerasuchus* are independent from each other and each one originates from a major cusp, so there are three major cusps that are well separated from each other along the broad mesial margin of the crown.

The keel of molariform teeth of MZSP-PV 139 merges with the rest of the crown differently on the buccal and lingual surfaces. In the maxillary teeth, the lingual surface of the multicusped keel is continuous with the lingual surface of the rest of the crown (Figure 30A, D). The buccal surface of the keel in the upper dentition is separated from the rest of the crown by a deep groove that extends apicobasally (Figures 30A, D), parallel to the multicusped keel. The depth of this groove increases at the mid-height of the keel and virtually disappears at the apical and basal region of the crown. In the lower molariforms the continuous surface is located on the buccal surface and the deep groove is located on the lingual surface (Figure 30F, G), as the tooth crowns are inversely oriented with respect to the maxillary molariforms.

The broad and rounded end of the tear-drop shaped crown lacks a distinct keel in all molariform teeth of MZSP-PV 139, but bears prominent and well separated apicobasally oriented ridges (Figure 30) that are more developed than in the incisiviform teeth. There are six to eight ridges on the buccal surface and nine to twelve ridges on the lingual surface of the molariform teeth. The development of these ridges varies, some of them are sharp and extend along the entire apicobasal height of the crown whereas others disappear at the crown midheight (Figures 30D, E, G, I) or merge with adjacent ridges (Figure 30D). These prominent ridges are present in *C. paulistanus*, *Caryonosuchus*, *S. huenei* and *Armadillosuchus* and slightly less developed in *Mariliasuchus* and *Notosuchus*. The sphagesaurids *Yacarerani* and *Adamantinasuchus*, instead, lack these well developed apicobasal ridges on the molariforms.

The enamel surface of the molariform crowns have, in addition to the denticled keel and the prominent apicobasal ridges two distinct patterns of enamel wrinkling. The broad (unkeeled) region of the crown has small and closely spaced globular protuberances scattered between the ridges (Figures 30H, I). This type of wrinking pattern was first described by Price [8] in *S. huenei* as the pebbled enamel surface. This type of enamel wrinkling is now known to be present also in other sphagesaurids (*Armadillosuchus*, *Caryonosuchus*, and in the distal surface of the crown of *Yacarerani*). The presence of pebbled enamel surface may be diagnostic of sphagesaurids [12] but there is variation in the development, density, and shape of these protuberances within this group. The poor preservation of the enamel surface in *C. montealtensis* precludes determining the condition of this taxon. The enamel surface of the molariforms of *Adamantinasuchus* seems to be smooth [6], [14] but the rugosities of *Yacarerani* are only visible under high magnification and SEM images (see [15]: figure 3) and therefore this condition in *Adamantinasuchus* is considered uncertain at the moment. The condition of sphagesaurids contrasts with the smooth enamel surface of *Notosuchus*, *Chimaerasuchus*, basal notosuchians (e.g., *Araripesuchus*, *Uruguaysuchus*, *Candidodon*, *Simosuchus*), sebecids, peirosaurids, and baurusuchids. In *Mariliasuchus* the wrinkling pattern of the enamel surface seems to be variable, as some specimens have anastomizing ridges mixed with globular protuberances (MZSP-PV 51).

The enamel surface of the denticles bears a different pattern of enamel wrinkling. Here, elongated wrinkles are oriented perpendicularly to keel and extend on the buccal and lingual surface of the denticles (Figures 30E, F). These enamel wrinkles are sinuous and merge and bifurcate irregularly, in an anastomosing pattern. The enamel wrinkling pattern of the basal denticles is less developed than in the large central denticles, being completely absent in the two basalmost denticles. Along the non-grooved side of the crown (lingual surface in maxillary teeth) these elongated enamel wrinkles extend on surface of the crown and cut and interrupt the apicobasal ridges (Figure 30E).

#### Wear facets

Extensive wear facets are exclusively present in the molariform teeth of MZSP-PV 139. The molariform teeth are highly specialized and the upper and lower teeth closely matched with each other producing extensive wear facets. These facets are present in both lower and upper molariform teeth and are mostly restricted to the lingual surface of the distal keel in the maxillary teeth (Figure 30D) and on the buccal surface of the mesial keel in the posterior dentary teeth (Figure 30H).

The facets resemble in their general morphology, orientation, and location on the surface of the crown to the facets described for *S. huenei* [5], *Mariliasuchus* [17], [64], [68], *Notosuchus* [61], and *Caryonosuchus* [13]. Similarly, this kind of wear facets is also present in *Yacarerani* and *C. paulistanus*. Two other notosuchian taxa, *Pakasuchus* and *Chimaerasuchus*, have been described with well-developed wear facets. The facets of these two taxa are located horizontally on the molariform crowns (parallel to the longitudinal plane of the skull), contrasting with the obliquely oriented plane of the wear facets of sphagesaurids, *Notosuchus*, and *Mariliasuchus*. The obliquely oriented wear facets of these taxa has been regarded as evidence that they share a derived jaw motion that included not only a large fore-aft component but also a lateral component [5], [61]. As in *S. huenei* and *Mariliasuchus*, the dentine and enamel surface of the wear facets have numerous striae that are straight, closely spaced, and subparallel to each other (Figure 30J). To date, the only sphagesaurid in which there are well-preserved molariforms that lack the well-developed oblique wear facets is *Adamantinasuchus* [14], although Osi ([3]: fig. Thirty-four F) noted the presence of similarly positioned and oriented small wear facets. The absence (or small development) of such facets in this taxon is intriguing given the similarities in its dentition with other sphagesaurids (in particular with *Yacarerani*), and could be related to different dietary habit and/or to the juvenile stage of its holotype, as suggested by Marinho & Carvalho [6] and Osi [3].

### Phylogenetic relationships

#### Phylogenetic dataset

The phylogenetic relationships of *Caipirasuchus stenognathus* were tested using a data matrix expanded both in terms of taxon and character sampling from a recently published dataset [26]. This dataset contains a large sample of non-eusuchian mesoeucrocodylians and basal crocodyliforms. In particular this dataset contains a broad sample of Cretaceous crocodyliforms from Gondwana that were recently described, including the eight known sphagesaurids, which were only represented by *S. huenei* in the dataset of [26]. The taxon sampling of other groups of Notosuchia was improved with the addition of peirosaurids (*Montealtosuchus*), mahajangasuchids (*Kaprosuchus*), and baurusuchids (*B. salgadoensis*, *B. albertoi*, *Campinasuchus*, *Pissarrachampsa*). The phylogenetic dataset has a total of 109 taxa scored across 412 characters. The character sampling scheme was improved through the addition of 65 characters that describe the variation in the skull, mandible, and dentition of notosuchian mesoeucrocodylians. The added characters include 34 new characters and 31 characters published in recent phylogenetic studies [10], [15], [18], [20], [21], [23], [24], [29], [30], [56]. A complete list of characters and the phylogenetic data matrix are provided as Document S1 and Dataset S1, respectively.

#### Phylogenetic analysis

**Figure 31:**
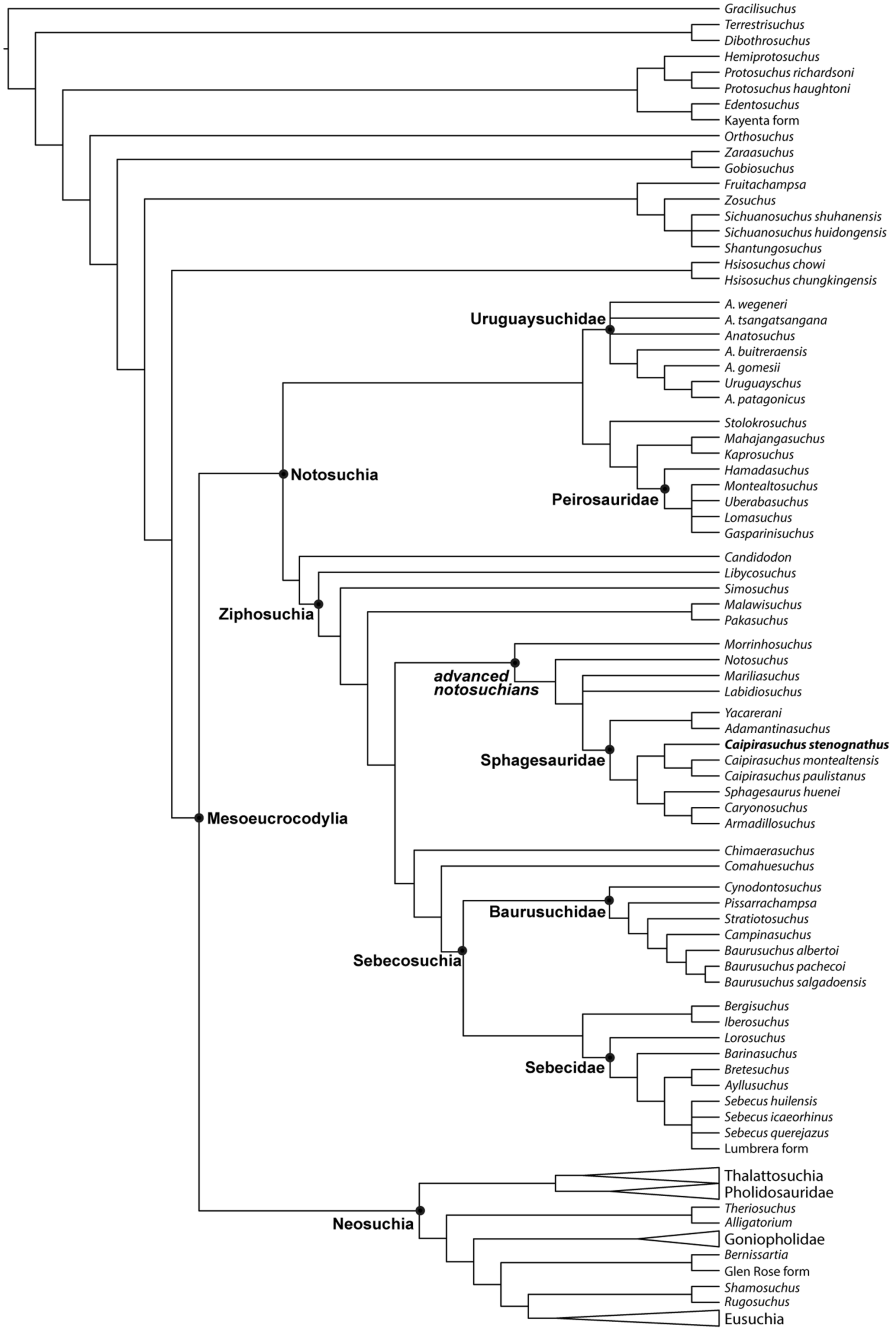
Phylogenetic relationships of Notosuchia within the context of Crocodyliformes. The reduced strict consensus is shown here after pruning the fragmentary taxa *Pehuenchesuchus*, *Pabhweshi*, and *Coringasuchus*.

This phylogenetic dataset was analyzed with equally weighted parsimony using TNT v. 1.0 [69], [70]. A heuristic tree search strategy was conducted performing 10000 replicates of Wagner trees (using random addition sequences) followed by TBR branch swapping (holding 10 trees per replicate). The best trees obtained at the end of the replicates were subjected to a final round of TBR branch swapping. Zero length branches were collapsed if they lack support under any of the most parsimonious reconstructions (i.e., rule 1 of Coddington & Scharff [71]). This analysis resulted in 6990 most parsimonious trees of 1598 steps (CI = 0.309, RI = 0.745), found in 1325 out of the 10000 replicates. TBR branch swapping of these 6990 trees found additional optimal topologies, yielding a total of 38880 most parsimonious trees (MPTs), which belong to a single island of MPTs. The unstable behavior of some taxa in the MPTs was analyzed using the script IterPCR [72], which identified the fragmentary *Pehuenchesuchus*, *Pabwehshi*, and *Coringasuchus* as the three taxa that cause large polytomies (the first two at the base of Sebecosuchia and the last one among advanced notosuchians). The following discussion of the phylogenetic results and synapomorphies of different nodes is based on the reduced strict consensus obtained by pruning these three fragmentary taxa after conducting the tree searches (Figure 31).

Branch support of clades was evaluated examining the most parsimonious trees in which the monophyly of a given group is rejected [73], as well as bootstrap and jackknife frequencies in TNT. Bremer support was evaluated using the script BREMER.RUN distributed with TNT [70] that uses a combination of heuristic tree searches that save suboptimal trees with constraints for non-monophyly. Resampling measures of support were calculated in TNT performing 1000 pseudoreplicates of bootstrap and jackknife resampling of characters. For the sake of simplicity, bootstrap and jackknife pseudoreplicates are discussed in the text based on their absolute frequencies, but both absolute and GC frequencies are given in Document S1. All branch support analyses (i.e., tree searches) included the unstable *Pehuenchesuchus*, *Pabwehshi*, and *Coringasuchus* but these taxa were pruned from the resulting trees to summarize the results. A similar procedure was conducted in some particular cases to test the effect of other incompletely known taxa (e.g. *Labidiosuchus*, *Morrinhosuchus*, *Chimaerasuchus*) on support values of specific nodes (see below). Additionally, alternative topologies on the relationships of some notosuchians (such as those obtained in previous studies) were tested through the use of heuristic tree searches under monophyly or non-monophyly constraints in TNT. This procedure aims to evaluate how many extra steps these alternative topologies require within the context of this dataset.

#### General Results

In general terms, all MPTs depict an overall similar pattern of relationships among basal mesoeucrocodylians as the study of the original data matrix [26] but provide improved resolution of some clades (e.g., uruguaysuchids, peirosaurids, mahajangasuchids) and a different pattern of relationships among advanced notosuchians (see below). The basalmost clade of Notosuchia clusters Uruguaysuchidae (i.e., *Anatosuchus*, *Araripesuchus* spp., and *Uruguaysuchus*) as the sister group of Peirosauridae and Mahajangasuchidae (Figure 31). All other notosuchians are grouped together in a large and diverse clade, which has its basal forms recorded in Cretaceous beds of Africa and South America (e.g., *Candidodon*, *Libycosuchus*, *Malawisuchus*, *Pakasuchus*) and two major derived clades: Sebecosuchia (i.e., Baurusuchidae and Sebecidae) and a derived clade of advanced notosuchians (which includes Sphagesauridae).

The major topological change with respect with the analysis of Pol *et al.* [26] as well as other recent studies [11], [12], [15], [18], [19], [21] is the monophyly of this clade of advanced notosuchians (Figure 31), which partially agrees with the recent results of Andrade *et al.* [20]. Our results show this clade of derived notosuchians as exclusively found in the Late Cretaceous of South America, and composed by *Morrinhosuchus*, *Notosuchus*, *Coringasuchus*, *Mariliasuchus*, *Labidiosuchus*, and a monophyletic Sphagesauridae (which includes *Adamantinasuchus* and *Yacarerani* but excludes non-South America taxa such as *Chimaerasuchus* and *Pakasuchus*). We will focus our discussion on the interrelationships and character support of this group of advanced notosuchians and their closely related taxa.

#### Relationships of Sphagesauridae

**Figure 32:**
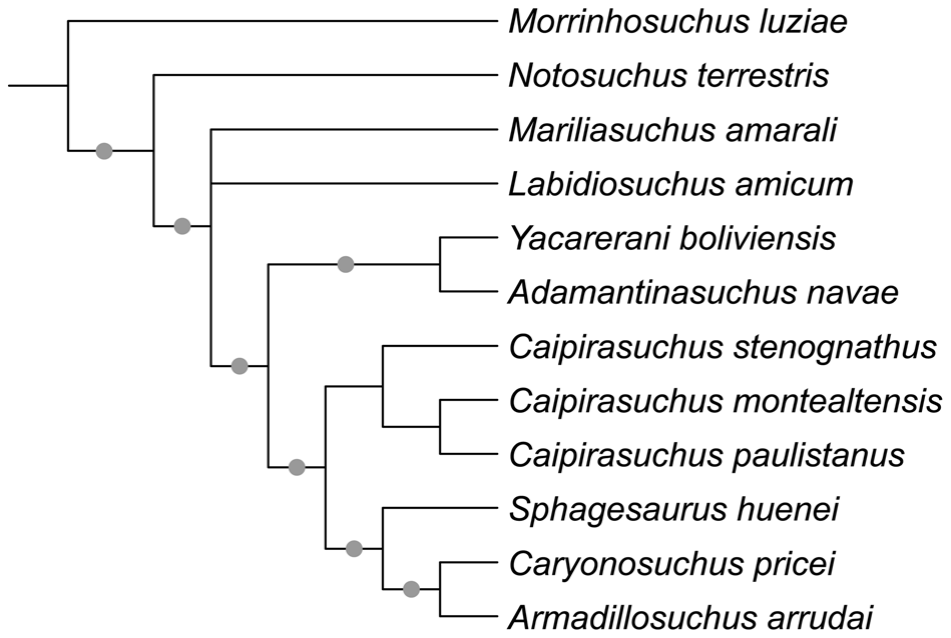
Phylogenetic relationships of advanced notosuchians. The relationships show the reduced strict consensus and the grey circles represent the alternative positions that the fragmentary *Coringasuchus* take in the MPTs.

**Figure 33:**
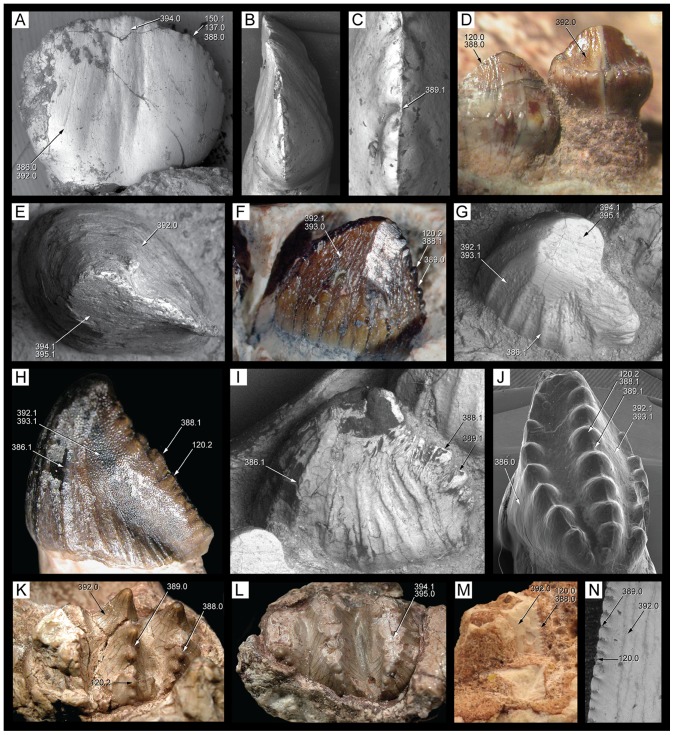
Dental characters of notosuchians. **A–C**, posterior tooth of *Uruguaysuchus aznarezi* FC-DPV 2320 in buccal (**A**) and mesial (**B–C**) views; **D**, *Candidodon itapecurense* UFRJ 114R posterior maxillary teeth in lingual view; **E**, *Nototsuchus terrestris* MACN-PV RN 1127 sixth maxillary tooth in occlusal view; **F**, *Mariliasuchus amarali* MZUSP-PV 51 second maxillary tooth in lingual view; **G**, *Caipirasuchus stenognathus* MZSP-PV 139 sixth lower molariform tooth in distobuccal view; **H**, *Armadillosuchus arrudai* UFRJ DG 303-R molariform tooth in lingual view; **I**, *Caipirasuchus stenognathus* MZSP-PV 139 fifth lower molariform tooth in distobuccal view; **J**, *Yacarerani boliviensis* MNK-PAL 5063 sixth lower molariform tooth in mesiolingual view; **K**, *Chimaerasuchus paradoxus* IVPP V8274 sencond maxillary tooth in occlusal view; **L**, *Chimaerasuchus paradoxus* IVPP V8274 worn maxillary tooth in occlusal view; **M**, *Comahuesuchus brachybuccalis* MUC-PV 202 lower caniniform tooth in buccal view; **N**, Sebecidae indet. MPEF-PV 10824 serrated carina of isolated ziphodont teeth in buccal view.

All most parsimonious trees depict a monophyletic Sphagesauridae, which is formed by the basal clade that includes *Yacarerani* and *Adamantinasuchus*, the *Caipirasuchus* clade, and a clade of derived sphagesaurids formed by *Sphagesaurs huenei*, *Armadillosuchus*, and *Caryonosuchus* (Figure 32). The monophyly of Sphagesauridae is supported by the presence of seven unambiguous synapomorphies. Two of these synapomorphies are related to derived features of the dentition of this clade: the enamel surface of maxillary and posterior dentary teeth are covered with small rounded bulges (char 393.1; Figure 33), described as “pebbled-enamel” by Price [8], and the large tuberous denticles of the keel of these teeth are connected by a thin enamel ridge (loph), instead of being separated by distinct and deep interdenticular slits (char. 389.1; Figure 33).

**Figure 34:**
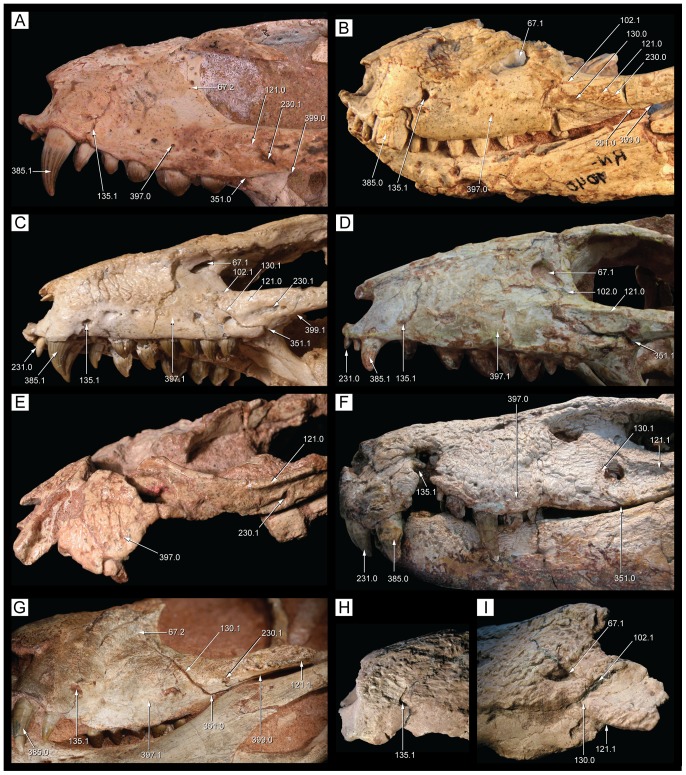
Rostral characters of notosuchians in lateral view. **A**, *Mariliasuchus amarali* MN 6756-V; **B**, *Notosuchus terrestris* MACN-PV RN 1040; **C**, *Caipirasuchus stenognathus* MZSP-PV 139; **D**, *Caipirasuchus paulistanus* MPMA 67-0001/00; **E**, *Comahuesuchus brachybuccalis* MOZ 6131P; **F**, *Baurusuchus salgadoensis* MPMA 62-0001/02; **G**, *Yacarerani boliviensis* MNK-PAL 5063; **H-I**, *Chimaerasuchus paradoxus* IVPP V8274, anterior rostral region (**H**) and posterior rostral region (**I**).

**Figure 35:**
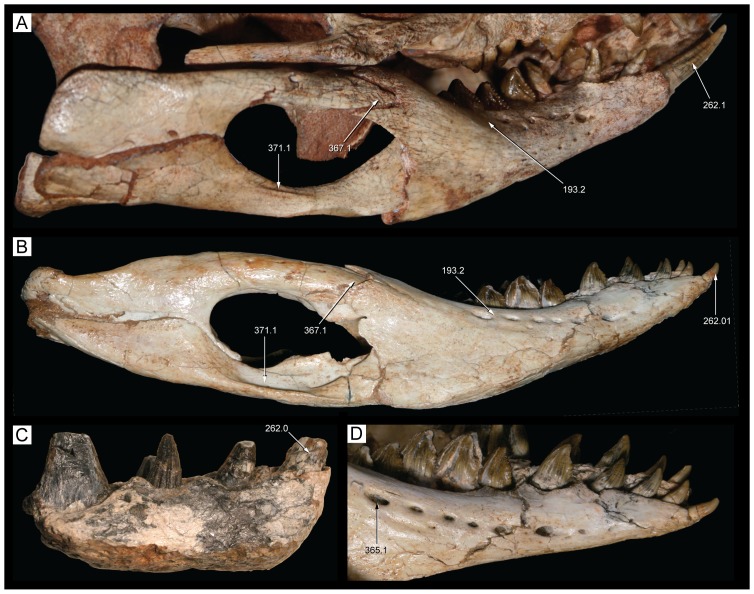
Mandibular characters of sphagesaurid notosuchians in lateral view. **A**, *Yacarerani boliviensis* MNK-PAL 5063; **B**, *Caipirasuchus stenognathus* MZSP-PV 139; **C**, *Sphagesaurus huenei* RCL-100; **D**, *Caipirasuchus stenognathus* MZSP-PV 139.

**Figure 36:**
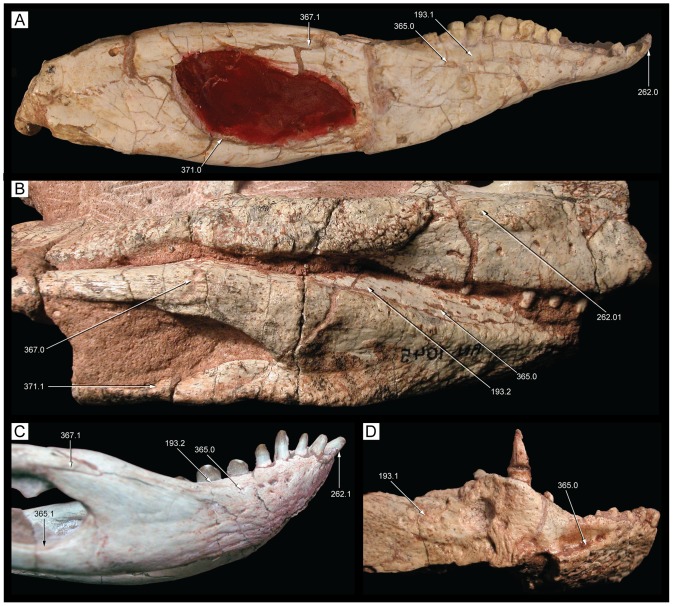
Mandibular characters of notosuchians in lateral view. **A**, *Uruguaysuchus aznarezi* FC-DPV 2320; **B**, *Notosuchus terrestris* MACN-PV RN 1045; **C**, *Mariliasuchus amarali* MN 6756-V; **D**, *Comahuesuchus brachybuccalis* MOZ 6131P.

Two other synapomorphic features are related to the particular pattern of neurovascular openings present close to the alveolar margins of the upper and lower jaws. Sphagesaurids are characterized by a gap between the anterior and posterior series of aligned neurovascular foramina of the lateral surface of maxilla. This derived feature was noted by Turner & Sertich [18] as shared by *S. huenei*, *C. montealtensis*, and *Adamantinasuchus*, but the same condition is also present in *Yacarerani*, *C. paulistanus*, and *C. stenognathus*, and therefore is optimized as an unambiguous synapomorphy of sphagesaurids (char. 397.1; Figure 34). Furthermore, these taxa are also characterized by the presence of remarkably large neurovascular foramina along the mid to posterior region of alveolar edge of the dentary, which are approximately as anteroposteriorly long as an alveolus (char 365.1; Figures 35–36).

**Figure 37:**
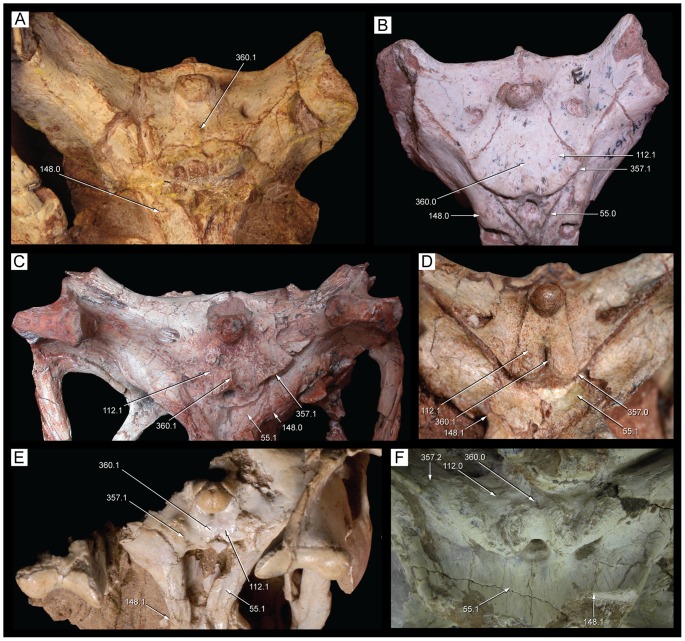
Basicranial characters of advanced notosuchians in ventral view. **A**, *Notosuchus terrestris* MACN-PV RN 1037; **B**, *Notosuchus terrestris* MLP 64-IV-16-2; **C**, *Mariliasuchus amarali* MZSP-PV 51; **D**, *Yacarerani boliviensis* MNK-PAL 5063; **E**, *Caipirasuchus stenognathus* MZSP-PV 139; **F**, *Armadillosuchus arrudai* UFRJ DG 303-R.

**Figure 38:**
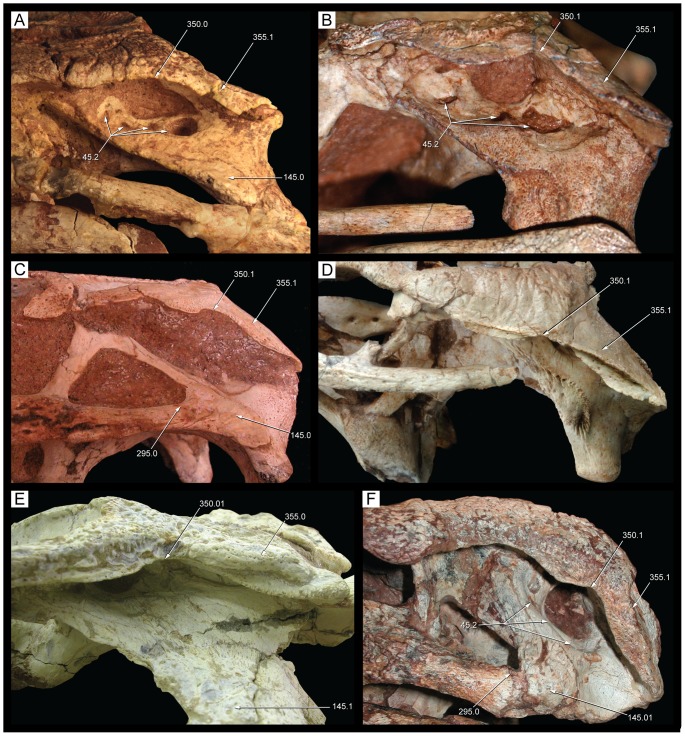
Infratemporal characters of advanced notosuchians in lateral view. **A**, *Notosuchus terrestris* MACN-PV RN 1037; **B**, *Yacarerani boliviensis* MNK-PAL 5063; **C**, *Mariliasuchus amarali* MN 6756-V; **D**, *Caipirasuchus stenognathus* MZSP-PV 139; **E**, *Armadillosuchus arrudai* UFRJ DG 303-R; **F**, *Baurusuchus salgadoensis* MPMA 62-0001/02.

**Figure 39:**
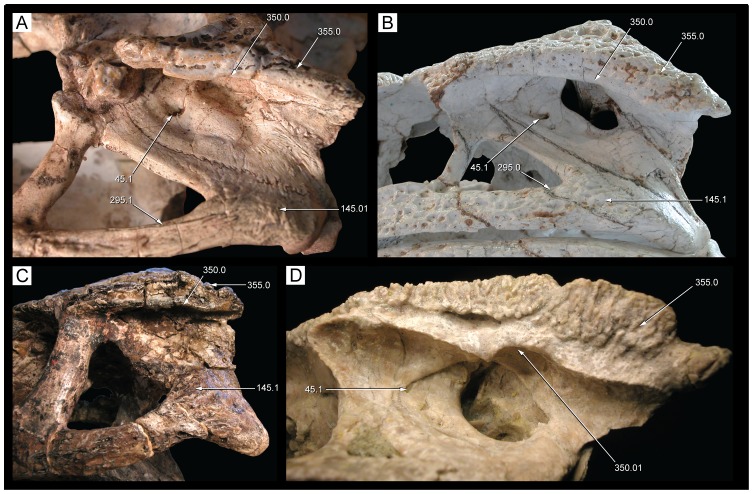
Infratemporal characters of basal notosuchians in lateral view. **A**, *Araripesuchus wegeneri* MNN GAD19; **B**, *Montealtosuchus arrudacamposi* MPMA 16-0007/04; **C**, *Libycosuchus brevirostres* BSP 1912.VIII.574; **D**, *Simosuchus clarki* UA 8679.

Finally, three skull characters are synapomorphic of Sphagesauridae. In all sphagesaurids that have preserved the braincase, the quadrate processes of pterygoids are extremely short and fail to extend along the lateral margins of the basisphenoid, ending far away from the level of the lateral eustachian openings (char. 148.1; Figure 37). The anterior process of quadratojugal forms the posterior third of the ventral margin of the infratemporal fenestra (char. 295.1) in sphagesaurids but in other advanced notosuchians (and baurusuchids) this process is extremely short or absent (Figure 38). The condition of the quadratojugal of sphagesaurids, however, is not unique and is also present in uruguaysuchids, peirosaurids, and longirrostrine crocodyliforms [23] (Figure 39). The suture between the postorbital and the squamosal of the three only sphagesaurids that have preserved this region (i.e., *Yacarerani*, *C. stenognathus*, *Armadillosuchus*) is anteriorly convex in lateral view (char. 411.1). This derived feature was noted as a synapomorphic character of the genus *Baurusuchus* [29], [30] and is optimized as convergently acquired in sphagesaurids in our analysis.

**Figure 40:**
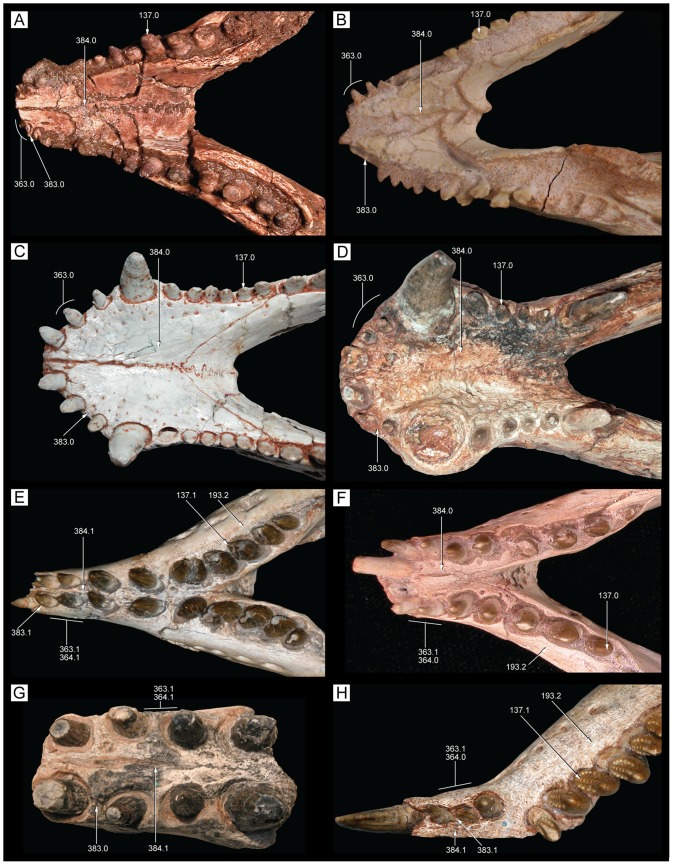
Mandibular sympyhseal characters of notosuchians in dorsal view. **A**, *Araripesuchus* MPCA-PV 236; **B**, *Uruguaysuchus aznarezi* FC-DPV 2320; **C**, *Montealtosuchus arrudacamposi* MPMA 16-0007/04; **D**, *Baurusuchus salgadoensis* MPMA 62-0001/02; **E**, *Caipirasuchus stenognathus* MZSP-PV 139; **F**, *Mariliasuchus amarali* MN 6756-V; **G**, *Sphagesaurus huenei* RCL-100; **H**, *Yacarerani boliviensis* MNK-PAL 5063.

Other derived features present in some sphagesaurids provide potential synapomorphies of this group that are ambiguously optimized due to the existence of character conflict or lack of information in the basalmost members of this clade. The most conspicuous of these characters was originally proposed by Andrade & Bertini [10], who noted that in *S. huenei* and *C. montealtensis* the left and right toothrow along mandibular symphysis are closely located from each other (forming a symphyseal tooth battery; Figures 5, 40). This feature is currently known to be present also in all the species of *Caipirasuchus*, *Armadillosuchus*, *Caryonosuchus*, and *Yacarerani* (the condition of *Adamantinasuchus* could not be determined based on the holotype). This unusual disposition of the lower toothrow along the symphyhsis is certainly characteristic of sphagesaurids and is absent in all other notosuchians (including *Mariliasuchus* and *Notosuchus*), with the exception of the fragmentary *Labidiosuchus* [74]. The presence of a symphyseal tooth battery in *Labidiosuchus* suggests a close affinity of this taxon with sphagesaurids, although other features, such as the absence of basal constriction in the posteriormost dentary tooth in *Labidiosuchus* (shared by *Mariliasuchus* and sphagesaurids) suggests a more basal position for this fragmentary taxon. More complete remains of *Labidiosuchus* are necessary to determine its putative affinities with Sphagesauridae.

The nodal support for Sphagesauridae is low if all taxa are included in the support analysis (Bremer = 1; boostrap and jackknife frequencies below 50%) but this occurs because the fragmentary taxon *Labidiosuchus* can be positioned within this clade with a single extra step. Running the support analysis ignoring the alternative positions of *Labidiosuchus* reveals high character support for the monophyly of Sphagesauridae to the exclusion of all other notosuchians (including *Mariliasuchus*, *Notosuchus*, and *Morrinhosuchus*). Bremer support value increases to 4 and bootstrap and jackknife values increase to 71% and 81%, respectively. This indicates a strong character support for the monophyly of Sphagesauridae, as in two recent analyses [20], [21] but contradicting previous studies that included more than one sphagesaurid and rejected the monophyly of this clade [11], [12], [15], [18]. The low support for the exclusion of *Labidiosuchus* from this group is due to the presence of sphagesaurid characters and the abundant missing entries of this taxon.

The clade *Caipirasuchus* includes *C. paulistanus*, *C. montealtensis*, *and C. stenognathus*, the latter the sister group of the two previously described taxa (Figure 32). The monophyly of the genus *Caipirasuchus* is supported by the presence of three unambiguous synapomorphies. These three species share the presence of a small antorbital fenestra (char. 67.1; Figure 34C–D), a reversal to the condition of more basal notosuchians that contrasts with the absence of this opening in other sphagesaurids and *Mariliasuchus* (Figure 34A). The only other sphagesaurid that may have an antorbital fenestra is *Adamantinasuchus*. Although Nobre & Carvalho [14] described this taxon as lacking an antorbital fenestra the right side of the holotype seems to have a small notch at the anterior margin of the lacrimal. This may represent a reduced antorbital fenestra although the poor preservation of the type specimen (and its likely juvenile ontogenetic stage) precludes determining this feature with certainty. Nonetheless, if this notch actually represents an antorbital opening in *Adamantinasuchus*, it would differ from the proportionately larger antorbital fenestra of *Caipirasuchus*.

The jugal of the three species of *Caipirasuchus* also shares the presence of an anteroventral short triangular process that wedges between the ectopterygoid and maxilla on the lateroventral surface of the skull (char. 351.1; Figure 34). This feature was described as an autapomorphic feature of *C. montealtensis* by Andrade & Bertini [10] but is now known to be present in the three species of this genus. Another synapomorphic feature of *Caipirasuchus* is the extremely long and narrow anterior (parallel sided) process of dentary symphysis, being approximately three times as long as wide (char. 364.2; Figure 40). The anterior end of the symphysis of *C. montealtensis* is missing but the preserved portion indicates it was proportionally long and narrow (Figure 5B) in comparison with the shorter and broader anterior (parallel sided) process of dentary symphysis of all other sphagesaurids (e.g., *S. huenei*, *Armadillosuchus*, *Caryonosuchus*, *Yacarerani*, *Adamantinasuchus*; Figure 40) and other advanced notosuchians (*Mariliasuchus*, *Labidiosuchus*, *Notosuchus*, *Morrinhosuchus*).

**Figure 41:**
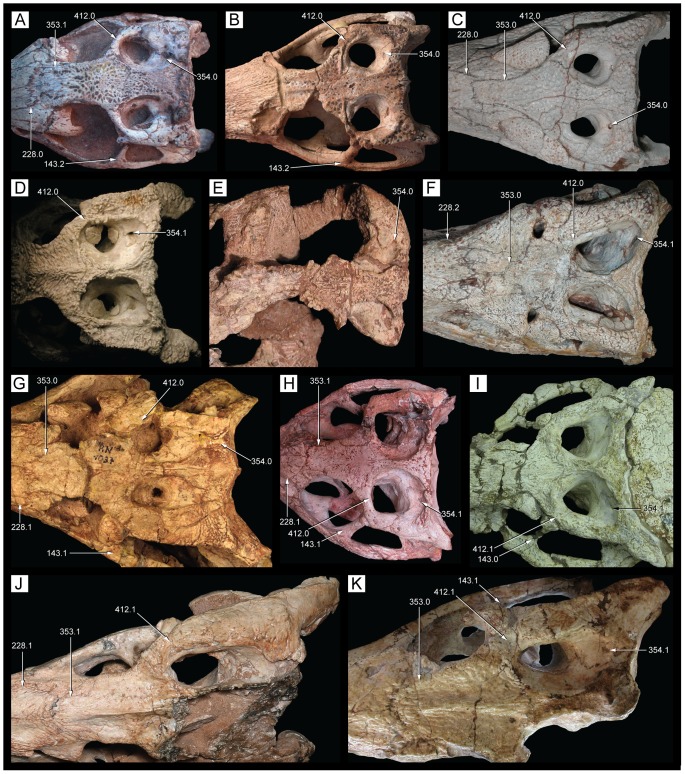
Supratemporal characters of notosuchians in dorsal view. **A**, *Araripesuchus patagonicus* MUC-PV 269; **B**, *Araripesuchus wegeneri* MNN GAD19; **C**, *Montealtosuchus arrudacamposi* MPMA 16-0007/04; **D**, *Simosuchus clarki* UA 8679; **E**, *Comahuesuchus brachybuccalis* MOZ 6131P; **F**, *Baurusuchus salgadoensis* MPMA 62-0001/02; **G**, *Notosuchus terrestris* MACN-PV RN 1037; **H**, *Mariliasuchus amarali* MZSP-PV 51; **I**, *Armadillosuchus arrudai* UFRJ DG 303-R; **J**, *Caipirasuchus stenognathus* MZSP-PV 139; **K**, *Caipirasuchus montealtensis* MPMA 15-001/90.

Two other features shared by the species of *Caipirasuchus* are optimized as ambiguous synapomorphies of this clade due to the presence of character conflict. The anterior region of the jugal of the three species of *Caipirasuchus* has thin longitudinal ridge that lacks a well-developed depression underneath it (char. 121.0; Figures 1, 34), resembling the condition of some non-sphagesaurid notosuchians (e.g., *Mariliasuchus*; Figure 34). Other sphagesarurids have a much more developed ridge (e.g., *Adamantinasuchus*, *Yacarerani*; Figure 34G) or a laterally projected shelf (e.g., *S. huenei*) and a markedly depressed surface ventral to the ridge or shelf. Finally, the anterolateral margin of the supratemporal fenestra is interrupted by transversely oriented groove that extends across the dorsal surface of the postorbital in *C. montealtensis* and *C. stenognathus* (char. 412.1; Figure 41J–K). The holotype of *C. paulistanus* has a depressed and unornamented surface on this region but the poor preservation of this specimen precludes determining the presence of this feature with certainty. Furthermore, this feature is incipiently present in *Armadillosuchus* (Figure 41I) but unknown in *S. huenei* and *Caryonosuchus*. Therefore the presence of this groove may diagnose *Caipirasuchus* or a more inclusive clade (including the above-mentioned sphagesaurids), as this unusual feature of the skull roof is absent in *Yacarerani*, *Adamantinasuchus*, and other advanced notosuchians (e.g., *Mariliasuchus*, *Notosuchus*; Figure 41).

The nodal support for *Caipirasuchus* is moderate, with Bremer support values of 2 and bootstrap and jackknife values of 67% and 77%, respectively. Suboptimal topologies and trees derived from the resampling techniques that violate the monophyly of *Caipirasuchus* show *C. stenognathus* in a more basal position or a paraphyletic arrangement of the three species with respect to other sphagesaurids.

**Figure 42:**
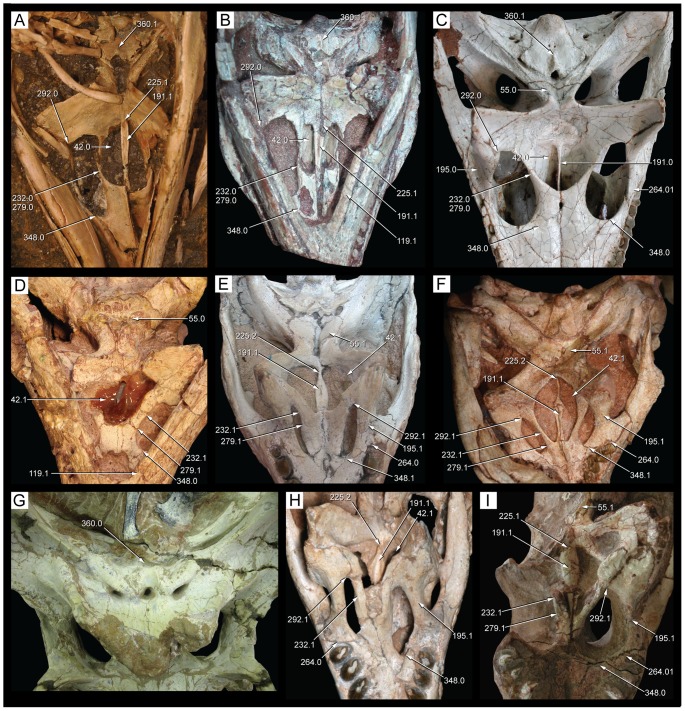
Choanal characters of notosuchians in ventral view. **A**, *Araripesuchus gomesii* AMNH 24550; **B**, *Araripesuchus patagonicus* MUC-PV 269; **C**, *Montealtosuchus arrudacamposi* MPMA 16-0007/04; **D**, *Notosuchus terrestris* MACN-PV RN 1037; **E**, *Mariliasuchus amarali* MN 6756-V; **F**, *Yacarerani boliviensis* MNK-PAL 5063; **G**, *Armadillosuchus arrudai* UFRJ DG 303-R; **H**, *Caipirasuchus stenognathus* MZSP-PV 139; **I**, *Caipirasuchus montealtensis* MPMA 15-001/90.

*Caipirasuchus paulistanus* and *C. montealtensis* depicted as sister taxa (Figure 32) based on the presence of two unambiguous synapomorphies. The first synapomorphy of this node concerns the flat ventral surface of the choanal septum of these two taxa, which is posteriorly broad (char. 225.1; Figures 4, 42) instead of tapering posteriorly as in *C. stenognathus* and other advanced notosuchians (*Yacarerani*, *Mariliasuchus*, *Notosuchus*; Figure 42). The second synapomorphic feature concerns the frontal shape along its suture with the prefrontal. Both *C. montealtensis* and *C. paulistanus* have the lateral margins of the anterior region of the frontal oblique to each other so that the frontal tapers gradually anteriorly (char. 353.0; Figures 2, 41K), resembling the condition of other notosuchians (e.g., *Notosuchus*, *Comahuesuchus*, *Malawisuchus*; Figure 41). In contrast, *C. stenognathus*, *Armadillosuchus*, *Yacarerani*, and *Mariliasuchus* have their anterior region of the frontal tabular-shaped with the lateral margins of the frontal parallel to each other (along their suture with the prefrontal; Figure 41). Therefore, within the context of Sphagesauridae, the obliquely oriented lateral margin of the anterior region of the frontal in *C. paulistanus* and *C. montealtensis* is interpreted as a reversal to the condition of more basal notosuchians.

Additionally, these two taxa share the presence of a distinct anterodorsal process in the anterior end of the jugal that not only contacts the lacrimal but also forms the posteroventral corner of the antorbital fossa (char. 102.0; Figures 1, 34). The jugal participation on the antorbital fossa is uncommon among notosuchians and is certainly absent in *C. stenognathus* (Figure 1C). However, the participation of the jugal in the antorbital fossa of *S. huenei* (RCL-100) and the inapplicable scorings (due to the absence of a clearly defined antorbital fossa) of closely related taxa (e.g., *Yacarerani*, *Adamantinasuchus*, *Mariliasuchus*) make this feature an ambiguous synapomorphy of the clade formed by *C. montealtensis* and *C. paulistanus*.

The nodal support for the node joining *C. montealtensis* and *C. paulistanus* has similar values as the node of *Caipirasuchus*, as in trees with two extra steps the monophyly of this clade is rejected. The bootstrap and jackknife values are 72% and 75%, and these values do not increase if the fragmentary *Labidiosuchus* is ignored in the support analyses.

*Caipirasuchus* is depicted as the sister group of the clade formed by *Sphagesaurus huenei*, *Caryonosuchus pricei*, and *Armadillosuchus arrudai* (Figure 32). This clade clusters all sphagesaurids except for *Yacarerani* and *Adamantinasuchus* and is diagnosed by five unambiguous synapomorphies.

Two synapomorphies are related to the shape of the symphyseal region of the mandible. The anterior (parallel sided) process of dentary symphysis of these sphagesaurids is elongated, being at least twice as long as wide (char 364.1; Figure 40). As noted above, this condition is further developed by *Caipirasuchus* that has an even longer and narrower anterior process of the mandibular sympyhsis (char. 364.2; Figure 40). The successive sister taxa of this clade (e.g., *Yacarerani*, *Adamantinasuchus*, *Notosuchus*, *Labidiosuchus*, *Mariliasuchus*; Figure 40F, H) have a shorter and broader anterior process of the mandibular symphysis, being approximately as long as wide. The second synapomorphic feature is the absence of strongly procumbent anterior dentary alveoli (char. 262.0; Figure 35). The three successive sister taxa of this clade (*Labidiosuchus*, *Mariliasuchus*, and the clade formed by *Adamantinasuchus* and *Yacarerani*) have horizontally projected anterior dentary teeth (Figures 35–36) and therefore within the context of sphagesaurids, the absence of highly procumbent teeth in the members of this clade is interpreted as a reversal to the plesiomorphic condition found in other notsuchians (e.g., *Notosuchus*, *Simosuchus*, *Libycosuchus*, baurusuchids). It must be noted that *Armadillosuchus* was distinguished from other sphagesaurids by its procument anterior dentary teeth [7], [12], however, this taxon lacks the strongly procumbent (horizontally directed) teeth present in the above mentioned taxa.

**Figure 43:**
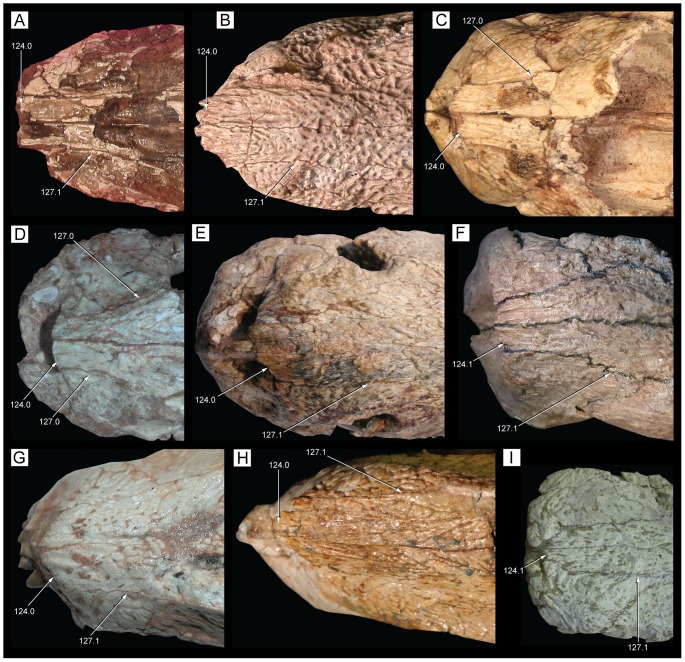
Rostral characters of notosuchians in dorsal view. **A**, *Araripesuchus buitreraensis* MUC-PV 235; **B**, *Araripesuchus wegeneri* MNN GAD19; **C**, *Notosuchus terrestris* MACN-PV RN 1040; **D**, *Comahuesuchus brachybuccalis* MUC-PV 202; **E**, *Baurusuchus salgadoensis* MPMA 62-0001/02; **F**, *Chimaerasuchus paradoxus* IVPP V8274; **G**, *Mariliasuchus amarali* URC R67; **H**, *Caipirasuchus stenognathus* MZSP-PV 139; **I**, *Armadillosuchus arrudai* UFRJ DG 303-R.

Three cranial characters are optimized as unambiguous synapomorphies of the clade formed by *Caipirasuchus*, *S. huenei*, *Caryonosuchus*, and *Armadillosuchus*. The nasal-premaxilla suture of these forms is straight (char. 127.1; unknown in *Caryonosuchus*), resembling the condition of the basalmost notosuchians, baurusuchids, and sebecids (Figure 43). This contrasts with the laterally concave premaxilla-nasal suture of other advanced notosuchians (i.e., *Adamantinasuchus*, *Yacarerani*, *Mariliasuchus*, *Morrinhosuchus*, and *Notosuchus*; Figure 43), and therefore the straight premaxilla-nasal suture of derived sphagesaurids is optimized as an unambiguous synapomorphy. This feature is subject to ontogenetic variation in some taxa (e.g., *Mariliasuchus*), showing a straight nasal-premaxilla suture in young specimens (Figure 43G) but laterally concave in more mature individuals (MZSP-PV 50 [17]). The second cranial synapomorphy of this node is the absence of a sagittal crest on the basioccipital (char. 360.0; Figure 37). Turner & Sertich [18] introduced this character, scoring this condition in *Armadillosuchus* and *Comahuesuchus*. The basioccipital of *Armadillosuchus* (Figure 37F) certainly lacks this ridge, as well as that of *C. stenognathus* (Figure 37E) and *S. huenei* (RCL-100). Although this feature is unknown in other species of *Caipirasuchus* and in *Caryonosuchus*, the absence of a basioccipital sagittal ridge is optimized as an unambiguous synapomorphy of these sphagesaurids given that this ridge is present in *Yacarerani*, *Mariliasuchus* (Figure 37), and most other notosuchians (e.g., baurusuchids, sebecids, *Candidodon*, uruguaysuchids, and peirosaurids; Figure 42). It should be noted that although *Notosuchus* was scored [18] with the absence of this ridge, this trait is variable among the available material as the ridge is present in some specimens of this taxon (e.g., MACN-RN 1037; Figure 37A). The third cranial synapomorphy of this clade is the presence of a notch in the ventral margin of the jugal at the level of the posterior end of the ectopterygoid-jugal suture (char. 399.1), a feature present in the three species of *Caipirasuchus* (Figure 34C) and *S. huenei* (but unknown in *Caryonosuchus* and *Armadillosuchus*). This feature was described by Montefeltro *et al.* [30] for baurusuchids, but is also present in *Iberosuchus*, *Sebecus icaeorhinus*, and mahajangasuchids. This notch is absent in other advanced notosuchians (e.g., *Adamantinasuchus*, *Yacarerani*, *Mariliasuchus*, *Notosuchus*; Figure 34) and therefore is optimized in our results as an unambiguous synapomorphy of this clade, being convergently acquired by sebecosuchians and mahajangasuchids.

Our analysis also retrieves two ambiguous synapomorphies of the clade formed by *Caipirasuchus*, *S. huenei*, *Caryonosuchus*, and *Armadillosuchus* that have been previously proposed [15] as derived features supporting the monophyly of a clade formed by *Mariliasuchus*, *Adamantinasuchus* and *Yacarerani*. The presence of procumbent premaxillary alveoli (char 231.1; Figure 34) and the exclusion of the maxilla from the anterior margin of the suborbital fenestra due to the anterior ectopterygoid-palatine contact (char. 348.1; Figure 42) are features recorded in *Mariliasuchus* and *Yacarerani*. The absence of these features in other sphagesaurids and more basal notosuchians (e.g., *Notosuchus*, *Candidodon*, baurusuchids; Figures 34, 42) creates an ambiguous optimization of this character, and diagnose the clade formed by *Caipirasuchus* and more derived sphagesaurids only under accelerated transformation.

The nodal support for this node is relatively high, with Bremer values of 3 and bootstrap and jackknife values of 67% and 77%, respectively. Trees that violate the monophyly of this group usually depicts the clade of *Yacarerani* and *Adamantinasuchus* as closer to either *Caipirasuchus* or the clade of large bodied sphagesaurids, but maintain the monophyly of Sphagesauridae (which has higher support values if the suboptimal positions of *Labidiosuchus* are ignored; see above).

The clade formed by *Sphagesaurus huenei*, *Caryonosuchus pricei*, and *Armadillosuchus arrudai* (Figure 32) clusters the most derived and large bodied sphagesaurids, whose skulls are up to twice the length of those of more basal sphagesaurids (e.g., *Yacarerani*, *Caipirasuchus*). This clade of large bodied sphagesaurids is diagnosed by eight unambiguous synapomorphies.

**Figure 44:**
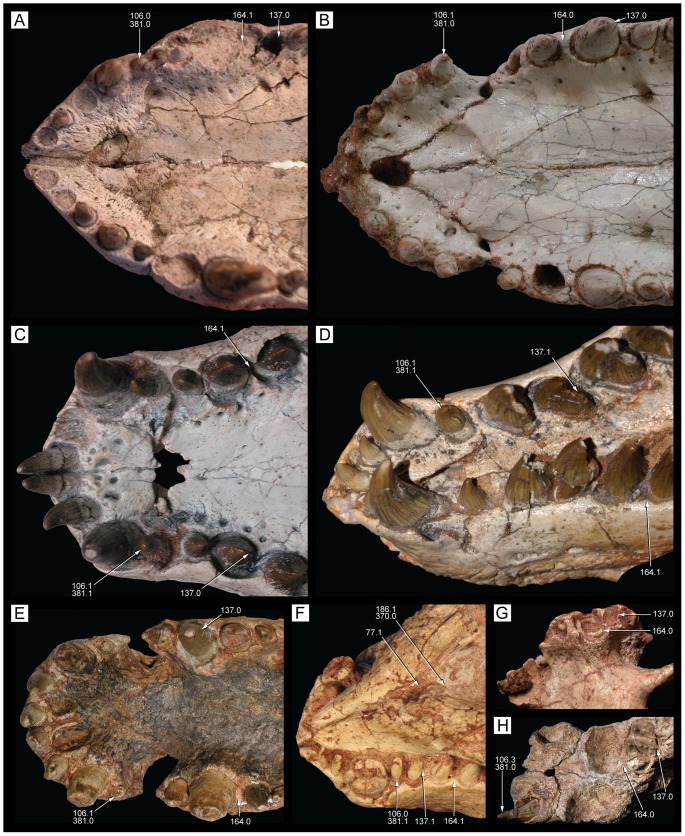
Rostral characters of notosuchians in ventral view. **A**, *Araripesuchus wegeneri* MNN GAD19; **B**, *Montealtosuchus arrudacamposi* MPMA 16-0007/04; **C**, *Mariliasuchus amarali* MZSP-PV 50; **D**, *Caipirasuchus stenognathus* MZSP-PV 139; **E**, *Baurusuchus salgadoensis* MPMA 62-0001/02; **F**, *Notosuchus terrestris* MACN-PV RN 1040; **G**, *Comahuesuchus brachybuccalis* MUC-PV 202; **H**, *Chimaerasuchus paradoxus* IVPP V8274.

Three of these synapomorphies concern the modified condition of the dentition of these taxa. These three taxa share the presence of only two premaxillary teeth (char. 106.3), whereas all other sphagesaurids and *Mariliasuchus* have four premaxillary teeth (Figure 44). The holotype of *C. montealtensis* has preserved only two teeth [10] but the specimen described by Iori *et al.* [12] shows the presence of four alveoli. The presence of only two premaxillary teeth is a drastic reduction and is unique among Crocodyliformes, with the exception of *Chimaerasuchus paradoxus* from the Early Cretaceous of China (Figure 44H). This similarity was part of the evidence for linking *S. huenei* and the Chinese taxon [5], but the results from our analysis (with improved taxon and character sampling) shows the reduction in the premaxillary dentition occurred convergently in this lineage of derived sphagesaurids and in *Chimaerasuchus*.

The second synapomorphy of this node is the absence of a “transitional tooth” located at the contact between the premaxilla and maxilla (char. 381.0). In contrast, all other advanced notosuchians have a postcaniniform alveolus that is formed by both the premaxilla and maxilla (Figure 44). This condition was first described for *Notosuchus* and *Mariliasuchus* [10], [61], [75], and was noted to be a unique condition among Crocodyliformes. The same condition is now known to be present also in *Caipirasuchus*, *Yacarerani*, *Adamantinasuchus*, and *Morrinhosuchus*. The absence of this feature in the clade of large bodied sphagesaurids is therefore optimized as an unambiguous synapomorphy of this clade, representing a reversal to the plesiomorphic condition of basal notosuchians and other crocodyliforms (Figure 44). Both *C. montealtensis* and *Adamantinasuchus* have been scored as lacking a tooth at the premaxilla-maxilla contact in previous studies [10], [18]. The condition of *C. montealtensis* is difficult to determine given the alveolar region is covered with sediment in its holotype, although the right premaxilla forms the anterolateral margin of this alveolus and the right maxilla forms the posterior margin. The condition of this taxon was therefore scored with character state 1. The holotype of *Adamantinasuchus* also has a small postcaniniform tooth located right at the premaxilla-maxilla contact and this taxon is also scored with character state 1 in our dataset.

The third synapomorphy of this clade is that all maxillary teeth are set in discrete alveoli (164.0). The distribution of this character has a similar pattern as the previously described synapomorphy, given that *S. huenei* and *Armadillosuchus* have the maxillary teeth set in discrete alveoli (the condition is unknown in *Caryonosuchus*) but all other sphagesaurids (*Yacarerani*, *Adamantinasuchus*, *Capirasuchus*; Figure 44D) and more basal notosuchians (e.g., *Mariliasuchus*, *Notosuchus*, *Morrinhosuchus*, *Pakasuchus*, *Malawisuchus*, *Simosuchus*, *Libycosuchus*, uruguaysuchids) have their teeth located on a continuous maxillary alveolar groove (Figure 44). The absence of this feature in the clade of large bodied sphagesaurids is therefore optimized as an unambiguous synapomorphy of this clade.

The other five unambiguous synapomorphies of this clade are found in skull characters, inferred from the condition of *S. huenei* (RCL-100) and *Armadillosuchus*, due to the fragmentary nature of the skull of *Caryonosuchus*. One of these concerns the morphology of the external nares, two the infratemporal region, and two the peculiar braincase anatomy of these forms.

The anterior tip of the nasals taper markedly along the anterior region of their contact with the premaxilla in both *S. huenei* (RCL-100) and *Armadillosuchus*, so that the dorsal margin of the external nares has only a minor contribution from the nasals (char. 124.1; Figure 43I) and a large contribution of the premaxillae. Other sphagesaurids (*Adamantinasuchus*, *Yacarerani*, *C. stenognathus*; Figure 43H) and all other notosuchians, instead, have the dorsal margin of the external nares formed mostly by the nasals, which do not taper anteriorly along their contact with the premaxilla (Figure 43). The only exception is in *Chimaerasuchus* that also has a reduced contribution of the nares to the dorsal margin of the nares (Figure 43F). As noted above, the exclusion of the nasals from the margin of the external nares described for *C. paulistanus* [11] is uncertain and the nasals seems to taper only anteriorly to the external nares (as in *C. stenognathus*), rather than along the anterior region of their lateral sutures with the premaxillae (as in *Armadillosuchus* and *S. huenei*).

The postorbital process of the jugal of *S. huenei* (RCL-100) and *Armadillosuchus* is located on the anterior half of this bone, being the infratemporal region longer than the suborbital and antorbital regions (char. 143.0; Figure 41I). All other sphagesaurids and notosuchians have a much more posteriorly positioned postorbital bar of the jugal (Figure 41) and therefore this feature is optimized as an unambiguous synapomorphy of this clade of derived and large bodied sphagesaurids.

The base of the quadratojugal of *S. huenei* (RCL-100) and *Armadillosuchus* is ornamented, at the posteroventral corner of the infratemporal fenestra (char. 145.1; Figure 38E). *Caipirasuchus*, *Mariliasuchus*, and other notosuchians (e.g., *Malawisuchus*, *A. gomesii*) have an unornamented base of the quadratojugal (Figure 38) and this character is optimized as a synapomorphy of this node. This character, however, shows several instances of homoplasy within Notosuchia, as the quadratojugal is ornamented also in several members of this clade (e.g., *Simosuchus*, *Stratiotosuchus*, sebecids, mahajangasuchids, peirosaurids; Figure 39).

The two derived characters of the braincase present in *S. huenei* (RCL-100) and *Armadillosuchus* that are optimized as unambiguous synapomorphies are: presence of a vertically oriented basioccipital (char. 112.0; Figure 37F) and presence of a quadrate-basioccipital contact on the occipital surface of the skull (char. 357.2; Figure 37F). These two characters are related to the highly modified anatomy of the basioccipital in these derived sphagesaurids but are considered as different characters given that they vary independently within Crocodyliformes. A vertically oriented basioccipital is also found in sebecids and neosuchians but contrasts with the condition of other notosuchians (including *Mariliasuchus*, *Notosuchus*, baurusuchids, and the sphagesaurids *Caipirasuchus* and *Yacarerani*; Figure 37) in which the basioccipital faces posteroventrally. The presence of a quadrate-basioccipital contact on the occipital surface of the skull of *S. huenei* and *Armadillosuchus* is unique among crocodyliforms, and is produced by a well developed medial crest of the quadrate that meets the basioccipital on the occipital surface of the skull [10]. This contact excludes the exoccipital from the ventral margin of the occipital surface, a character that was originally noted as autapomorphic of *S. huenei* [5] but is now also known to be present in *Armadillosuchus* (Figure 37F).

Finally, an additional ambiguous synapomorphy of this node is the presence of lower incisiviforms implanted in separate alveoli (char. 383.0; Figure 40G) that distinguish these taxa from other sphagesaurids (*C. stenognathus*, *C. paulistanus*, *Yacarerani*), which have the lower incisiviforms set in a continuous anterior alveolar groove of the dentary (Figure 40). This character is ambiguously optimized due to character conflict as the lower incisiviforms of other advanced notosuchians (e.g., *Notosuchus* [61], *Mariliasuchus* [17]) is also set in separate alveoli.

The support for the node that clusters *S. huenei*, *Armadillosuchus*, and *Caryonosuchus* is high both in terms of Bremer support and resampling measures. Bremer values show that the monophyly of this clade is only challenged in trees that are three steps longer than the MPTs. Bootstrap and jackknife values reach 91% and 92%, showing a high support for the clade of large bodied sphagesaurids even when fragmentary taxa such as *Labidiosuchus* are included in the support analyses.

In our analysis *Caryonosuchus pricei* and *Armadillosuchus arrudai* are depicted as sister taxa (Figure 32) given they share the presence of a single derived feature that is absent in the skull referred to *S. huenei* (RCL-100 [5]): the absence of a small neurovascular foramen located at the premaxilla-maxilla suture on the lateral surface of the rostrum (char. 135.0). This distinct foramen is present in all other sphagesaurids and most notosuchians (e.g., *Mariliasuchus*, *Notosuchus*, *Morrinhosuchus*, *Malawisuchus*, *Simosuchus*, baurusuchids, and most uruguaysuchids; Figure 34). The lack of more complete remains of *Caryonosuchus*, however, precludes testing more thoroughly its putative affinities with *Armadillosuchus* to the exclusion of *S. huenei*. Therefore, the character support for this group is minimal in terms of its Bremer support and low bootstrap and jackknife frequencies (64% and 62%).

Finally, the basalmost clade of sphagesaurids clusters *Adamantinasuchus navae* and *Yacarerani boliviensis* (Figure 32). These two small bodied and bizarre sphagesaurids are clustered together based on two unambiguous synapomorphies of their dentition. First, these two taxa are the only crocodyliforms in which the posterior teeth have a couple of accessory apicobasally oriented keels bearing tuberous denticles, which are located lingually and buccally from the major central keel (char. 391.1; Figure 33J). Second, the posterior teeth of *Adamantinasuchus* and *Yacarerani* lack the prominent and well-spaced apicobasal ridges on the enamel surface (char. 386.0; Figure 33J), which are present in all other sphagesaurids [5], [6], [10], [12], *Mariliasuchus* [17], [64], and *Notosuchus* [61], [75] (Figure 33). Therefore within the context of advanced notosuchians the absence of apicobasal ridges is interpreted as a reversal to the condition found in more basal crocodyliforms.

The support for the node that clusters *Yacarerani* and *Adamantinasuchus* is low, partially because of the lack of information for *Adamantinasuchus* (70% missing data). The Bremer support is minimal and the bootstrap and jackknife frequencies range between 51% and 64%, even when fragmentary taxa such as *Labidiosuchus* are ignored. The suboptimal topologies (with one extra step) and the trees from resampling techniques that reject the monophyly of *Yacarerani*+*Adamantinasuchus* place *Yacarerani* as closer to other sphagesaurids than *Adamantinasuchus*. These topologies imply an extra step for each of the two unambiguous synapomorphies of this clade (see above), but save a step in character 130 (dorsoventral expansion of the jugal anterior to the orbit). *Yacarerani* shares with other sphagesaurids a distinct expansion of the jugal anterior to the orbits (Figure 34), whereas *Adamantinasuchus* has an anteriorly tapering jugal as other notosuchians (*Mariliasuchus*, *Notosuchus*; Figure 34B). Further remains of *Adamantinasuchus* (particularly of adult specimens) are needed to test more thoroughly its sister group relationship with *Yacarerani*, supported by the unique and derived morphology of their molariform teeth.

#### Relationships of advanced notosuchians

Sphagesauridae is deeply nested within a clade of advanced notosuchians that also includes *Mariliasuchus*, *Labidiosuchus*, *Notosuchus*, and *Morrinhosuchus*. In all the MPTs the fragmentary *Coringasuchus* (only known by a fragment of a lower jaw [76]) also forms part of this clade but takes almost all possible positions (Figure 32) due to the lack of decisive information in the characters that can be scored in its holotype. This large monophyletic clade is exclusively recorded in the Late Cretaceous beds of Brazil, Bolivia, and Argentina.

This clade of advanced notosuchians is diagnosed by three unambiguous synapomorphies and eight ambiguous synapomorphies (due to the fragmentary condition of *Morrinhosuchus*). Two of the unambiguous synapomorphies of this clade were previously mentioned as they are reversed in subclades of Sphagesauridae. First, the nasal-premaxilla suture of *Morrinhosuchus*, *Notosuchus*, *Mariliasuchus*, *Yacararani*, and *Adamantinasuchus* is laterally concave (char. 127.1; Figure 43) and therefore this is optimized as an unambiguous synapomorphy of the clade of advanced notosuchians (showing a reversal in *Caipirasuchus* and more derive sphagesaurids). Second, the above-mentioned taxa and *Caipirasuchus* share the presence of a transitional tooth which is set in an alveolus that has its anterior half formed by the premaxilla and its posterior half formed by the maxilla (381.1; Figure 44) and therefore this character diagnoses the clade of advanced notosuchians, although it is subsequently modified in the derived clade of large-bodied sphagesaurids (see above).

The third unambiguous synapomorphy of advanced notosuchians is a noticeable feature of the mandibular symphysis. In all these forms the anterior region of the symphysis have a distinct anterior process, which has parallel lateral margins (363. 1; Figure 40). Andrade & Bertini [75] first noticed the similarities in the elongated mandibular symphysis of *Notosuchus* and *Mariliasuchus*. When viewed in ventral view, the symphyseal region of these taxa has a posterior region in which the lateral margins are oblique to each other (diverging posteriorly) and a distinct anterior process along which the lateral margin of each dentary are parallel to each other. This condition is now known to be present also in *Morrinhosuchus*, *Labidiosuchus*, and all sphagesaurids and therefore diagnose this clade of advanced notosuchians. All the basal members of this clade (incuding *Yacarerani* and *Adamantinasuchus*) have a relatively short anterior process of the symphysis (as broad as long), but this process becomes progressively elongated in more derived sphagesaurids (Figure 40).

The fragmentary nature of the type and only known specimen of *Morrinhosuchus* (i.e., a rostrum and anterior mandible [77]) precludes scoring this taxon for a large number of characters (78% of missing data). This taxon is the basalmost member of this clade and therefore creates an ambiguous optimization of several derived characters that distinguish advanced notosuchians from other taxa. The following list includes eight ambiguous synapomorphies that may diagnose the entire clade of advanced notosuchians (if the derived condition in *Morrinhosuchus* is confirmed by more complete remains) or the clade formed by *Notosuchus* and more derived forms (including sphagesaurids). Out of these eight ambiguous synapomorphies, three are from the skull, two from the mandible, and three from the dentition of these taxa.

The anterior region of the jugal has a large neurovascular foramen on its lateral surface (char. 230.1; Figure 34). This feature was originally described as a derived character of *Mariliasuchus* and *Comahuesuchus* [17], but in fact is present also in sphagesaurids (*Adamantinasuchus*, *Yacarerani*, *C. stenognathus*, *C. montealtensis*, *S. huenei*) and is polymorphic *Notosuchus* (Figure 34B), being present only in some specimens (e.g., MACN-RN 1041).

The choanal septum of *Notosuchus*, *Mariliasuchus*, and all sphagesaurids that have preserved this region shares the presence of a lateromedial expansion at its ventral end (char. 191.1; Figure 42). This condition was originally described for some of the South American species of *Araripesuchus* [58] (Figure 42A–B) but is also present in Mahajangasuchidae and *Simosuchus* in addition to the above-mentioned advanced notosuchians. The generalized condition within Notosuchia, nonetheless, is the absence of this ventral expansion (Figure 42C) and this feature is reconstructed as evolving independently four times within Notosuchia in our analysis.

The quadrate of advanced notosuchians has multiple fenestrae surrounding the otic aperture (char. 44.2), a condition described for *Notosuchus* [78], *Mariliasuchus* [17], [75], and also preserved in *Yacarerani* and *Caipirasuchus* (Figure 38). The multiple fenestrae in the quadrate of advanced notosuchians resemble the condition of basal crocodyliforms and therefore this character is optimized as a reversal to the plesiomorphic condition. Baurusuchids also have multiple fenestrae in the quadrate (Figure 38F), and some of which have them internalized within the otic notch [30], and provide within the context of this analysis an independent instance on the appearance of multiple quadrate fenestrae within Notosuchia (a reversal to the condition of basal crocodyliforms).

The two mandibular characters that are ambiguous synapomorphies of the clade of advanced notosuchians include modifications of the medial and lateral surfaces of the mandibular ramus. The first mandibular synapomorphy is the presence of an elongated fossa that extends along the ventral margin of the external mandibular fenestra (char. 371.1). This elongated fossa is exposed in lateral view, but is separated from the lateral surface of the angular by a sharp ridge that delimits the fossa ventrally. This feature is recorded in *Notosuchus*, *Mariliasuchus*, *Yacarerani*, and *C. stenognathus* (Figures 35–36), and is currently unknown in other members of this clade due to poor preservation of this region. This is a unique condition of advanced notosuchians as all other notosuchians (and other crocodyliforms) lack this distinct and laterally exposed fossa on the ventral margin of the external mandibular fenestra (Figure 36A).

**Figure 45:**
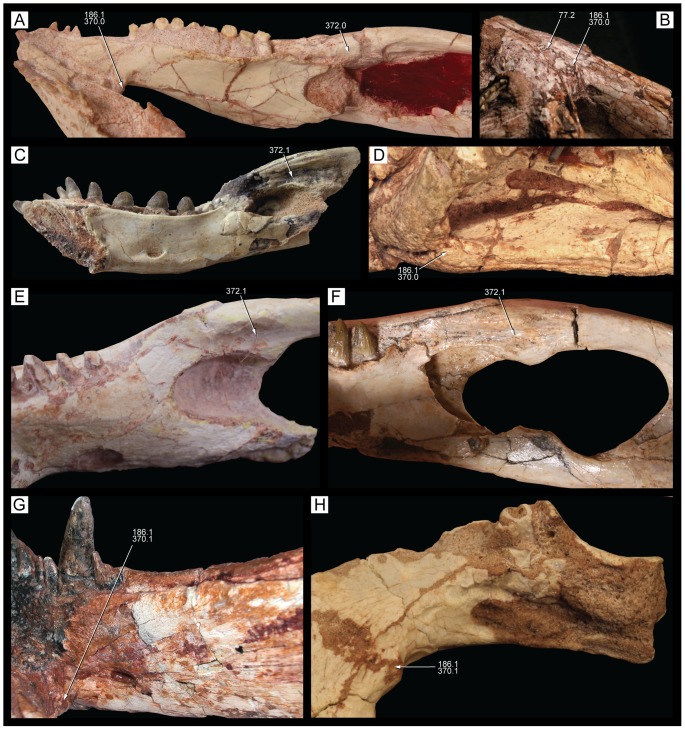
Characters of the mandibular ramus of notosuchians in medial view. **A**, *Uruguaysuchus aznarezi* FC-DPV 2320; **B**, *Araripesuchus buitreraensis* MUC-PV 235; **C**, *Mariliasuchus amarali* URC R68; **D**, *Notosuchus terrestris* MACN-PV RN 1037; **E**, *Caipirasuchus montealtensis* MPMA 15-001/90; **F**, *Caipirasuchus stenognathus* MZSP-PV 139; **G**, *Baurusuchus salgadoensis* MPMA 62-0001/02; **H**, *Comahuesuchus brachybuccalis* MACN-PV N 31.

The second mandibular synapomorphy is the presence of coronoid tuberosities forming elongated crests separated by a deep longitudinal sulcus on the medial surface of the anterior region of the surangular (char. 372.1; Figure 45). This character is recorded only in *Mariliasuchus*, *Yacarerani*, and *C. stenognathus*, but the condition of the other basal members of this clade (*Morrinhosuchus*, *Notosuchus*, *Labidiosuchus*) is currently unkown. Therefore, the coronoid tuberosities may be diagnostic of the entire clade or a subgroup of advanced notosuchians.

The three ambiguous synapomorphies of the dentition of advanced notosuchians are related to the peculiar morphology of the posterior molariforms of these taxa. The first one is the presence of apicobasal ridges on the enamel surface of posterior teeth (char. 386.1; Figure 33). This feature is extremely well developed in derived sphagesaurids and was one of the salient features used to diagnose *S. huenei* [8]. Similar ridges are also present in *Mariliasuchus* [64] (Figure 33F) and *Notosuchus* [61], [75]. Although the enamel ridges of molariforms in these two taxa are less developed than in derived sphagesaurids, their presence is diagnostic of most advanced notosuchians. This peculiar character is not free of homoplasy, as noted above, the clade of *Yacarerani* (Figure 33J) and *Adamantinasuchus* shows a reversal in this character as they lack the enamel ridges on posterior molariforms and the condition of *Morrinhosuchus* is currently unknown.

The second ambiguous synapomorphy of the dentition of advanced notosuchians is the particular shape and position of tooth-tooth occlusion wear facets on posterior upper and lower teeth. *Notosuchus*, *Mariliasuchus*, and most sphagesaurids have extensive and flat wear facets (char. 394.1; Figure 33) that extend mesiolingually from the apex of the crown in upper teeth and buccodistally from the apex in lower teeth. This unique orientation of the wear facets (Figure 33E, G) was first noted for *S. huenei* ([5]: fig. 8) and *Notosuchus* ([61]: fig. 6), but is now known in most members of this clade. Furthermore, these facets are oriented along a plane that is oblique to the longitudinal and sagittal planes of the skull (char. 395.1; Figure 33) and differs from the horizontally oriented wear facets of other notosuchians (e.g., *Pakasuchus*, *Chimaerasuchus*; Figure 33L). The orientation of upper and lower wear facets in advanced notosuchians was interpreted as evidence supporting not only the presence of fore-aft motion of the jaw but also an alternate unilateral occlusion of the right and left toothrows [5] (see [3] for a detailed account and functional inferences on some taxa). The only advanced notosuchian with well-preserved dentition that lacks well-developed wear facets is *Adamantinasuchus*, which is intriguing given the pattern of wear facets in advanced notosuchians is conservative even when there are marked differences in the molariform morphology. The lack of posterior teeth in *Morrinhosuchus* precludes determining if this character diagnoses all advanced notosuchians or only *Notosuchus* and more deverived forms.

The third ambiguous synapormorphy of the posterior teeth is the pattern of size variation of the denticles on the distal carina of upper teeth and mesial carina of lower teeth. *Marilisuchus* and sphagesaurids have denticles at the central region of the carinae that are approximately twice the size (in height and width) of both apical and basal denticles (char. 388.1; Figure 33). This differs from the rather homogeneous size of denticles in ziphodont crocodyliforms (e.g., baurusuchids, sebecids, peirosaurids; Figure 33M–N) and from the pattern present in *Chimaerasuchus* in which the denticles decrease gradually in size along the carina from the apex to the base of the crown (Figure 33K). The condition of *Morrinhosuchus*, *Notosuchus*, *Labidiosuchus* is unknown for this character so the pattern of denticle size variation may diagnose all advanced notosuchians or a restricted group formed by *Mariliasuchus* and sphagesaurids.

The nodal support for the clade of advanced notosuchians is high despite the incompleteness of its most basal taxon *Morrinhosuchus*. If the uncertain position of *Coringasuchus* is ignored, the monophyly of advanced notosuchians is rejected only in trees that are four steps longer than the MPTs, and bootstrap and jackknife values are 75% and 79%. These high values indicate a strong support for this group, reflecting the increase in character sampling for these forms and rejecting previous hypotheses that depicted some sphagesaurids as closer to baurusuchids than to *Notosuchus* or *Mariliasuchus* [5], [17], [18]; but agreeing with the initial study of Andrade & Bertini [10] on the affinities of *Sphagesaurus huenei* and *C. montealtensis* with *Notosuchus* and *Mariliasuschus* to the exclusion of baurusuchids [10]. The monophyly of a large group of advanced notosuchians that includes *Notosuchus*, *Mariliasuchus*, and most sphagesaurids was also supported in a more extensive analysis on crocodyliforms recently published by Andrade *et al.* [20].

*Morrinhosuchus* is depicted in all MPTs as the basalmost taxon of advanced notosuchians (Figure 29) and its basal position is supported by the absence of a single derived feature shared by *Notosuchus*, *Labidiosuchus*, *Mariliasuchus*, and sphagesaurids: the oblique orientation of distal carina on upper posterior teeth and mesial carina on lower posterior teeth (char. 137.1; Figures 40, 44). It must be noted that *Mariliasuchus* is polymorphic for this character as some specimens have a longitudinally oriented carina (Figures 40F, 44C) whereas others have an oblique orientation of the carina [17], [75].

The nodal support for this clade is extremely low, mainly because of the fragmentary nature of *Morrinhosuchus* (78% missing data). Bremer support is minimal and bootstrap and jackknife frequencies are below 50%. This low support is caused because *Morrinhosuchus* can be placed as the sister group of *Notosuchus* with a single extra step (a topology retrieved in 40% of the resampling replicates). Therefore, the exclusion of *Morrinhosuchus* from the clade formed by *Notosuchus* and more derived forms is poorly supported. More complete remains of this taxon are needed to test if it is indeed a basal form of this clade (as suggested by the lack of obliquely oriented posterior teeth) or if it forms the sister group of *Notosuchus*. Ignoring the alternative positions of *Morrinhosuchus* (and *Labidiosuchus*) increases the Bremer, bootstrap, and jackknife values to the ones described above for the entire clade of advanced notosuchians.

*Mariliasuchus* and *Labidiosuchus* are placed as closer to sphagesaurids than *Notosuchus* (Figure 32) based on the presence of three unambiguous synapomorphies of their mandibular and dental morphology. The first unambiguous synapomorphy is the presence of a posterodorsal branch of the dentary divided into a ventral and a dorsal process that is exposed on the lateral surface of the lower jaw; the dorsal process fits into the large notch between the medial and lateral rami of the bifurcated anterior end of the surangular (char. 367.1; Figures 35–36). Andrade & Bertini [10] noted this feature as shared by *Mariliasuchus* and *C. montealtensis* but scored this information as missing for *Notosuchus*. This derived feature is absent in *Notosuchus* (MACN-RN 1045; Figure 36B) but is present in all species of *Caipirasuchus*, *Adamantinasuchus*, *Yacarerani*, and *Labidiosuchus* (in addition to *Mariliasuchus*; Figures 35–36). As noted by Turner & Buckley [56], a similar articulation between the dentary and surangular is present in peirosaurids, although it is not present in *Kaprosuchus* and incipiently developed in *Mahajangasuchus* (in which the posterodorsal branch of the dentary has a very small dorsal process that fits between the medial and lateral rami of the anterior end of the surangular). This similarity in the articulation between the surangular and dentary between peirosaurids and the clade formed by *Mariliasuchus*, *Labidiosuchus* and sphagesaurids is interpreted as a convergent feature.

The anterior alveoli of the dentary are strongly procumbent in *Labidiosuchus*, *Mariliasuchus*, and the basalmost clade of sphagesaurids (*Adamantinasuchus* and *Yacarerani*). In these forms the first dentarry tooth (or first and second dentary teeth) are horizontally projected (char. 262.1; Figures 35–36) and differs from the anterodorsally directed anterior dentary teeth of *Notosuchus* and most other notosuchians (Figure 36). As mentioned above, more derived sphagesaurids show a reversal to the plesiomorphic condition (as seen in *Caipirasuchus* and *S. huenei*; Figure 35C).

The third unambiguous synapomorphy supporting the group of *Labidiosuchus*, *Mariliasuchus*, and sphagesaurids is the presence of a rugose outser enamel surface (char. 392.1; Figure 33). Turner & Sertich [18] noted this condition is shared by *Mariliasuchus* and sphagesaurids. Although these authors scored the presence of this feature in *Notosuchus* and the absence in *Yacarerani*, the enamel surface of the former taxon is smooth [61] (Figure 33E) and *Yacarerani* has small rounded rugosities [15] (Figure 33J) resembling the pebbled enamel of other sphagesaurids (Figure 33G–H). This character was scored in our dataset with character state 0 for *Notosuchus* and with character state 1 for *Yacarerani*, providing support for the clade clustering *Mariliasuchus*, *Labidiosuchus*, and sphagesaurids to the exclusion of *Notosuchus*.

Given that *Labidiosuchus amicum* [74] is only known by a partially preserved lower jaw, twelve derived features shared by *Mariliasuchus* and sphagesaurids are optimized as ambiguous synapomorphies of this node. These turn into unambiguous synapomorphies if *Labidiosuchus* is ignored or if it takes a more derived position than *Mariliasuchus* (as in some MPTs). The phylogenetic signal of these features provides decisive support for postulating *Mariliasuchus* as closer to sphagesaurids rather than to *Notosuchus*. Two of these twelve synapomorphies concerns the antorbital region, two of them the palatal morphology, three of them the skull roof and temporal region, one is on the basisphenoid morphology, three on the anatomy of the mandible, and one is from the dentition.

One of the rostral characters that link *Mariliasuchus* with sphagesaurids is the absence of an antorbital fenestra (char. 67.2; Figure 34), which is subsequently reversed in *Caipirasuchus*. The closure of the antorbital fenestra is optimized in our results as occurring independently seven times in the history of Crocodyliformes, three of which occurred within Notosuchia (i.e., in this clade of advanced notosuchians, in *Pakasuchus*, and in the clade that clusters *Comahuesuchus* with baurusuchids and sebecids).

The second rostral character is the presence of an orbital lamina in the maxilla (char. 352.1), a vertically oriented lamina located above the suborbital fenestra that restricts the opening of the nasal cavity into the orbit. This derived feature is present in *Mariliasuchus* (URC R68), *C. stenognathus*, *C. paulistanus*, and *S. huenei* but is unknown in other sphagesaurids. *Notosuchus* (MACN-RN 1039, 1045) lacks this derived feature of the maxilla and the posterior end of the palatal branch of the maxilla is dorsoventrally thin, leaving an ample connection between the nasal cavity and the orbit as in other crocodyliforms.

The palatal region of *Mariliasuchus* and sphagesaurids also shares two derived features absent in *Notosuchus*, related to the morphology of the ectopterygoid. First, the main axis of the ectopterygoid is oriented parasagitally in *Mariliasuchus* and sphagesaurids (char. 195.1; Figure 42), whereas in *Notosuchus* (Figure 42D) and most notosuchians is oriented obliquely to the longitudinal axis of the skull. Second, the anterior end of the ectopterygoid forms part of the alveolar margin of the posteriormost maxillary tooth (char. 264.0; Figure 42). This character is present in *Mariliasuchus*, *Yacarerani*, *C. paulistanus*, and *C. stenognathus* and therefore is optimized as a synapomorphy of this clade. In most other notosuchians (including *Notosuchus*) the anterior end of the ectopterygoid does not reach the posterior maxillary alveolus. It must be noted that this character shows a reversal to the plesiomorphic condition of Notosuchia in *S. huenei* and is ambiguous in *C. montealtensis* (which has the anterior end of the ectopterygoid extremely close to, but not reaching, the posteriormost maxillary tooth; Figure 42I).

The skull roof and supratemporal region of the skull of *Mariliasuchus* shares with sphagesaurids three derived characters absent in *Notosuchus*. The first synapomorphy the anterior region of the frontal of *Mariliasuchus* and all sphagesaurids (except for *C. paulistanus* and *C. montealtensis*) is tabular shaped, with the lateral sutures with prefrontals parallel to each other (char. 353.1; Figure 41). *Notosuchus* and more basal notosuchians have the lateral margins of the frontal oblique to each other, converging anteriorly.

The two other derived features of the supratemporal region are shared by *Mariliasuchus* and sphagesaurids, but also are present in baurusuchids and therefore are interpreted as convergently acquired in these two clades. The temporo-orbital foramen is enclosed between the parietal and squamosal in most crocodyliforms. However, *Mariliasuchus* and sphagesaurids have this opening completely included within the squamosal smooth surface on the posterior region of the supratemporal fossa (char. 354.1; Figure 41). This condition is recorded in *Yacarerani*, *C. montealtensis*, and *Armadillosuchus* but is unknown in other sphagesaurids. The exclusion of the parietal from the temporo-orbital foramen is a highly unusual condition and is present also in some but not all baurusuchids (Figure 42F). *Pissarrachampsa*, one of the most basal baurusuchids, has the temporo-orbital opening enclosed between the parietal and squamosal [30]. *Simosuchus* also has the parietal excluded from the temporo-orbital opening (Figure 42D), but in this bizarre taxon the morphology is slightly different as the quadrate contributes to this opening [18], [78].

The squamosal of *Mariliasuchus* and sphagesaurids also have a derived morphology on the outer margin of the shelf that overhangs the otic recess. This margin bears a markedly sinuous outline, with a highly convex ventral outgrowth on its anterior region and a small but highly concave notch located at the level of the otic aperture (char. 350.1; Figure 38). This feature was noted by Novas *et al.* [15] as shared by *Mariliasuchus* and *Yacarerani*, but is now known to be also present in *C. stenognathus* and (slightly less developed) in *Armadillosuchus* (Figure 38E). *Notosuchus* (Figure 38A) and almost all other notosuchians (Figure 39; see below), instead, have a straight anterior region of the ventral margin of the squamosal and lack (or have gently developed) the concavity at the level of the otic aperture. The only exception, as noted above, is found in most baurusuchids that also have the strongly sinuous ventral margin of the squamosal (Figure 38F), although *Stratiotosuchus* has only a gently concave notch at the level of the otic aperture. Finally, *Simosuchus* also has a rather strongly concave notch at the level of the otic aperture, but anteriorly to this point the ventral margin of the squamosal is straight rather than convex (Figure 39D).

The derived feature of the ventral surface of braincase shared by *Mariliasuchus* and sphagesaurids is the shape of the basisphenoid. First, the basisphenoid of these taxa has a notably broad ventral exposure (char. 55.1; Figure 37), a unique condition among notosuchians that resembles the morphology of basal crocodyliforms [27]. The basisphenoid of *Mariliasuchus* and sphagesaurids is anteriorly broad with curved anterolateral margins that give a semicircular shape to this bone in ventral view (Figure 37). This contrasts with the anteriorly narrow and V-shaped ventral exposure of the basisphenoid of all other mesoeucrocodylians (Figure 42C), including *Notosuchus* (Figure 37A–B).

The three derived features of the mandible shared by *Mariliasuchus* and sphagesaurids are found in the splenial and the retroarticular process. The splenial of all mesoeucrocodylians (Figure 42B), including *Notosuchus* (Figure 42D), has a lateral deflection at its ventral margin, so that this bone forms part of the (usually ornamented) ventral surface of the mandibular ramus. In *Mariliasuchus* and sphagesaurids (*Yacarerani*, *Caipirasuchus*) the splenial is restricted to the medial surface of the mandibular ramus and lacks the lateral deflection at its ventral margin (char. 119.0; Figures 23, 25), resembling in this feature the condition of basal crocodyliforms. The splenials of all notosuchians are sutured to each other at the mandibular sympyhsis and most of these forms have a peg along the posterior margin of the splenial-splenial symphysis [58]. *Notosuchus* (Figure 44F), *Morrinhosuchus*, and more basal notosuchians (e.g., uruguaysuchids; Figure 45A–B) have this peg located on the posteroventral corner of the splenial-splenial suture. *Mariliasuchus* and sphagesaurids (*Yacarerani*, *Caipirasuchus*) have modified this condition and bear a relatively small peg that is dorsally displaced from the ventral surface of the splenial-splenial suture (char. 370.1; Figure 25). This condition is also present in *Comahuesuchus* and baurusuchids (in which the peg is dorsoventrally elongated forming a short ridge; Figure 45G–H), but is optimized here as convergently acquired based on the topology of the MPTs.

**Figure 46:**
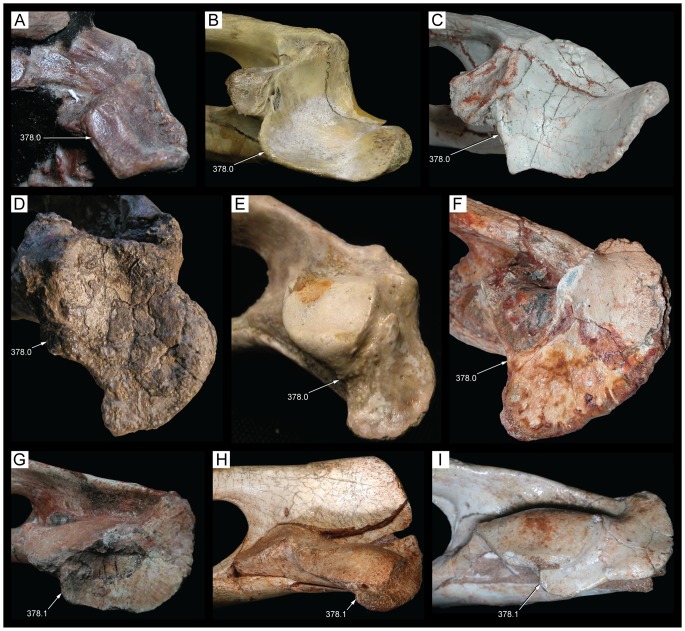
Characters of the retroarticular process of notosuchians and other crocodyliforms in posteromedial view. **A**, *Hsisosuchus chungkingensis* cast of CNM V 1090; **B**, *Caiman latirostris* FMNH 9713; **C**, *Montealtosuchus arrudacamposi* MPMA 16-0007/04; **D**, *Libycosuchus brevirostres* BSP 1912.VIII.574; **E**, *Simosuchus clarki* UA 8679; **F**, *Baurusuchus salgadoensis* MPMA 62-0001/02; **G**, *Mariliasuchus amarali* MZSP-PV 51; **H**, *Yacarerani boliviensis* MNK-PAL 5063; **I**, *Caipirasuchus stenognathus* MZSP-PV 139.

The retroarticular process of *Mariliasuchus* and sphagesaurids have a distinct anterior process at the anteromedial end of the medial flange that extends anteriorly up to the level of the anteroposterior midpoint of the medial glenoid facet of the articular (char. 378.1; Figure 46G–I). This distinct process is unique among crocodyliforms, as in most taxa (including *Notosuchus*) the anteromedial end of the medial flange is connected to the posteromedial corner of the medial glenoid facet of the articular through a dorsally directed crest (Figure 46A–F).

Finally, the dentition of *Mariliasuchus* shares with sphagesaurids (except for *Adamantinasuchus* and *Yacarerani*; Figure 34G) the presence of apicobasal ridges on the surface of the enamel of incisiviforms and caniniforms (char. 385.1; Figure 34). Although *Notosuchus* has these ridges in the molariforms (see above), its incisiviforms and caniniform are smooth [61] (Figure 34B) as in more basal notosuchians and other crocodyliforms.

As seen, all these features are absent in *Notosuchus* and provide evidence supporting the position of *Mariliasuchus* as closely related to sphagesaurids. When *Labidiosuchus* is considered in the support analyses, the node joining this taxon, *Mariliasuchus*, and sphagesaurids has a minimal Bremer support value (Bremer = 1) but is well supported by resampling techniques (bootstrap 83% and jackknife 88%). The fragmentary nature of *Labidiosuchus* makes possible to place this taxon more basally implying a single extra step, although the lack of features suggesting a more basal position produces high support in the resampling techniques. However, the large number of derived characters shared by *Mariliasuchus* and sphagesaurids noted above is reflected in notably high support values when *Labidiosuchus* is ignored in the support analyses. Doing such a procedure, the bremer support of this clade is 5 and the bootstrap and jackknife values are 93% and 97%, respectively. These results indicate the increased character sampling of our analysis is reflected in a notably high character support for the close affinities of *Mariliasuchus* with sphagesaurids to the exclusion of other notosuchians such as *Notosuchus* and *Comahuesuchus*. This result contradicts previous studies that depicted *Mariliasuchus* as closer to *Notosuchus* [10], [18], [20], [56] or to *Comahuesuchus* [17], [26].

#### The relationships of advanced notosuchians with *Comahuesuchus*, *Chimaerasuchus*, and baurusuchids

The large clade of advanced notosuchians is depicted as the sister group of *Comahuesuchus*+*Chimaerasuchus*+baurusuchids+sebecids (Figure 31). This large group is diagnosed by the presence of six unambiguous craniomandibular synapomorphies. Four of these synapomorphies represent the highly modified palatal morphology of these notosuchians, one character is found in the skull roof, and one the mandibular anatomy. This group is further diagnosed by a postcranial synapomorphy (char. 311.1 [26]). These synapomorphies are shared by advanced notosuchians, *Comahuesuchus*, and baurusuchids. Sebecids usually have internal variation of these features due to their highly modified cranial anatomy, but some of them share the presence of these derived features (see below). The absence of palate and skull roof in the holotype of *Chimaerasuchus* precludes determining the presence of this critical information in the Chinese taxon. The problematic affinities of *Chimaerasuchus* and *Comahuesuchus* are treated in the Discussion.

The posterior region of the palatines between the suborbital fenestra is slightly constricted and then flares posteriorly close to the anterior margin of the choana (char. 279.1; Figure 42). This condition is further modified in derived baurusuchids (*B. salgadoensis*, *B. pachecoi*), which have a much more developed constriction of the palatines [30]. More basal notosuchians, instead, lack this constriction and have the lateral margins of the palatines oriented subparallel to the longitudinal axis of the skull (e.g., *Pakasuchus*, *Malawisuchus*, *Candidodon*, *Araripesuchus*, peirosaurids; Figure 42A–C). Sebecids are depicted as nested within this group but their palatal morphology is modified. They lack the lateromedial constriction of the palatines between the suborbital fenestra, but the posterior region of the palatine is flared posteriorly in *Bretesuchus* (but not in *Sebecus icaeorhinus* or *Sebecus querejazus*).

Advanced notosuchians, *Comahuesuchus*, baurusuchids, and *Bretesuchus* have the posterior end of the palatines, along the lateral margins of the choanal opening, formed by a distinct rod-like process of the palatines that projects posteroventrally and laterally (char. 232.1; Figure 42). This process was referred as palatine bar by Zaher *et al.* [17] and was noted by other authors as shared by baurusuchids and advanced notosuchians [46], [55]. This distinct process of the palatines is absent in more basal notosuchians (*Pakasuchus*, *Malawisuchus*, *Simosuchus*, *Candidodon*, uruguaysuchids, peirosaurids; Figure 42A–C) and in derived sebecids (e.g. *Sebecus icaeorhinus*). An important feature of the palatine bars is that they are not sutured to the anteromedial margin of the pterygoids flanges, creating an accessory palatal fenestra that is located between the suborbital fenestra and the choanal groove. This distinguishes the morphology of the posterior region of the palatines of these taxa from that of other crocodyliforms, even those that have the posterior flaring of the palatines described in the character mentioned above (e.g., *Uruguaysuchus*, *Shamosuchus*, *Alligator*).

The posterior end of the palatine of advanced notosuchians, *Comahuesuchus*, and baurusuchids is sutured to the ectopterygoid (char. 292.1; Figure 42), a contact that is absent in all other notosuchians (Figure 42A–C). This suture is present in the above-mentioned taxa, all of which have rod-like palatine bars, but is uncertain if *Bretesuchus* had this contact (which is absent in other sebecids such as *Lorosuchus* and *Sebecus querejazus*). This character is considered as independent from the presence of a palatine bar as there are other crocodyliforms that have a palatine-ectopterygoid contact but lack rod-like palatine bars (e.g., *Caiman* [79], [80]).

The choanal opening of advanced notosuchians, *Comahuesuchus*, baurusuchids, and most sebecids (*Sebecus icaeorhinus*, *Barinasuchus*) is remarkably broad, occupying approximately 50% of the width between the lateral margins of the pterygoid flanges (char. 42.1; Figure 42). More basal notosuchians (*Pakasuchus*, *Malawisuchus*, *Simosuchus*, *Candidodon*, uruguaysuchids, peirosaurids; Figure 42A–C) have a much narrower choana that occupies less than 30% of the width of the pterygoid flanges. This condition is also present in some sebecids (*Lorosuchus*, *Bretesuchus*, *Sebecus querejazus*), which is optimized as reversals to the plesiomorphic condition in our analysis. The distribution of this character also shows that the presence of a broad choanal opening is not dependent on the characters above (presence or absence of a rod-like palatine bar or the posterior flaring of the palatines).

The squamosal of most advanced notosuchians, *Comahuesuchus*, and baurusuchids has an unornamented posterolateral process (char. 355.1; Figure 38), whereas the ornamentation of the skull roof extends onto the dorsal surface of this process in other notosuchians (*Pakasuchus*, *Simosuchus*, *Candidodon*, *Anatosuchus*, *Araripesuchus*, peirosaurids; Figure 39) and more basal crocodyliforms (e.g., *Hsisosuchus*). Two independent reversals to the plesiomorphic condition are recorded within this large clade. One of them is in *Armadillosuchus* (Figure 38E), which has extensive ornamentation of the squamosal extending along its posterolateral process. Given the absence of this region in other members of the clade of large bodied sphagesaurids (i.e., *Caryonosuchus*, *S. huenei*) it is currently unknown if this feature characterizes this clade or if it is autapomorphic of *Armadilosuchus*. The second reversal instance is present along the lineage leading to *Iberosuchus* and sebecids. All the members of this clade that have preserved a squamosal (*Iberosuchus*, *Sebecus icaeorhinus*) have the dorsal ornamentation of the skull roof extended onto the posterolateral process of the squamosal.

The mandibular character that provides support for the monophyly of this large clade that includes advanced notosuchians is that the splenial is only moderately involved in the mandibular symphysis. In ventral view, the splenial occupies 20% or less of the symphyseal length (char. 77.1; Figure 44F), whereas other notosuchians have a more extensive participation of the splenial, reaching 30% of the symphyseal length (*Pakasuchus*, *Malawisuchus*, *Anatosuchus*, *Uruguaysuchus*, *Araripesuchus*; Figure 45B). The latter condition is optimized as the plesiomorphic condition of Mesoeucrocodylia as it is also present in *Hsisosuchus* and basal neosuchians.

An additional ambiguous synapomorphy shared by all the members of this clade (including sebecids) is the presence of a posterolateral expansion of the nasals, which projects towards the articular facet for the anterior palpebral (char. 228.1; Figure 41). This process becomes more developed in baurusuchids and sebecids and extends further laterally, forming part of the lateral surface of the snout (char. 228.2; Figure 41F). In more basal notosuchians the nasals have a restricted posterolateral projection and they are well separated from the anterodorsal corner of the orbit (Figure 41A–C).

All these features directly support the close relationships of advanced notosuchians and baurusuchids, as they are absent in more basal notosuchians, including *Pakasuchus* from the Early Cretacous of Africa that has been postulated [19] as closely related to some sphagesaurids or advanced notosuchians. The support values for this large clade that clusters advanced notosuchians, *Comahuesuchus*, *Chimaerasuchus*, baurusuchids, and sebecids is notably low if all taxa are considered in the support analyses. Bremer support values are minimal and bootstrap and jackknife frequencies are below 50%. The reasons for this low support are found in the labile positions of some of its members, which are either poorly known with abundant missing entries (e.g., *Chimaerasuchus*) or exhibit notable character conflict (e.g., sebecids). If the alternative positions of the enigmatic *Chimaerasuchus* are ignored during the support analyses, the bremer support for this group increases up to 3 but the frequencies of bootstrap and jackknife are still below 50%. This indicates that although the inclusion of *Chimaerasuchus* in this large clade of notosuchians is weakly supported (see below for a discussion on this taxon), other taxa are also responsible for the low frequency of recovery of this clade in the resampling analyses.

A major problem in notosuchian phylogeny is the position of Sebecidae and there are two major phylogenetic signals that conflict with each other [48]. Sebecids (and closely related forms such as *Iberosuchus* and *Bergisuchus*) share derived similarities with baurusuchids [18], [22], [26], [48] and with peirosaurids [20], [21], [23], [24]. Our phylogenetic dataset includes characters representing both sets of derived similarities, and characters supporting the monophyly of Sebecosuchia (baurusuchids+sebecids) outweight the alternative signal in the MPTs. However, the alternative position of Sebecidae (more basally within Notosuchia and closer to peirosaurids) is a plausible hypothesis as it implies only four extra steps within the context of our analysis. This affects support measures of the node being discussed here as excluding sebecids violates its monophyly. Irrespective of the sebecid problem, it is important to assess the support in our dataset for clustering advanced notosuchians, *Comahuesuchus*, and baurusuchids to the exclusion of other notosuchians (such as *Pakasuchus*, *Malawisuchus*, *Simosuchus*, *Libycosuchus*, *Candidodon*, uruguaysuchids, and peirosaurids). Performing the support analyses ignoring the alternative positions of sebecids and the most fragmentary taxa of this clade (i.e., *Pehuenchesuchus*, *Pabwehshi*, *Coringasuchus*, *Chimaerasuchus*) reveals the existence of a strong support for clustering advanced notosuchians, *Comahuesuchus*, and baurusuchids. Bremer support increases to 4 and bootstrap and jackknife frequencies rises to 72% and 75% if sebecids and the above-mentioned fragmentary taxa are ignored. This reflects the strength of the evidence for the monophyly of these forms to the exclusion of other notosuchians in our dataset. Characters that increase in number of steps in the alternative topologies are precisely those that are listed above as synapomorphies of this group, four of which represent the highly modified palatal anatomy of these taxa.

## Discussion

### Implications of the monophyly of Notosuchia, advanced notosuchians, and sphagesaurids

The phylogenetic analysis presented here provides new information on the nature of the diversification events that made notosuchians one of the most conspicuous faunal components of the Cretaceous of Gondwana. The major clades of Notosuchia have their oldest records in the Early Cretaceous of different gondwanan land-masses (as well as in China). The pattern of phylogenetic relationships recovered here, coupled with the temporal and geographic record of notosuchians, suggest an important diversification event in the Early Cretaceous that marked the initial diversification of Notosuchia.

Furthermore, the recovery in our analysis of a monophyletic group clustering all sphagesaurids (*sensu* [6]) that is nested within a clade of advanced notosuchians has several implications for understanding the evolutionary history of notosuchian crocodyliforms in the Late Cretaceous of South America. The recovered pattern of phylogenetic relationships clarifies our understanding on the geographic and temporal radiation of this derived group of notosuchians as an important component of the Late Cretaceous terrestrial ecosystems in South America.

**Figure 47:**
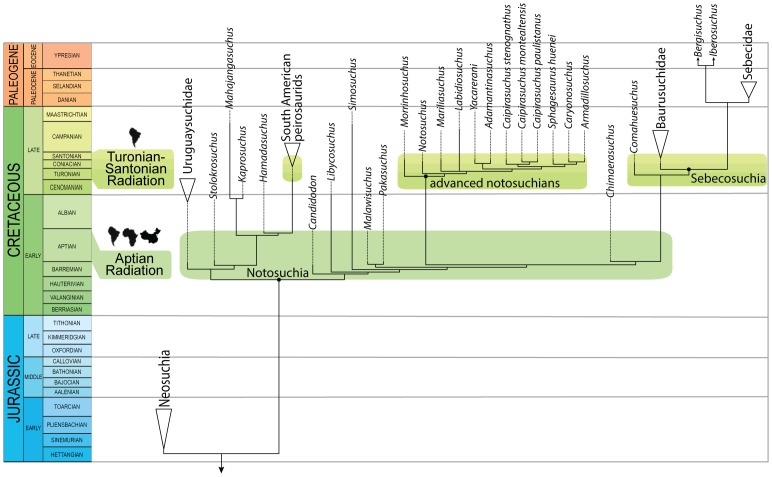
Calibrated phylogeny of Notosuchia based on the reduced strict consensus of our analysis. Dashed lines indicate the uncertainty on the age of each taxon. Major clades are collapsed (e.g., Neosuchia, Uruguaysuchidae, peirosaurids, Baurusuchidae, Sebecidae) and the age of the their oldest member is used for inferring the age of these nodes. The clade peirosaurids refer to South American forms (see Figure 28). Two distinct radiation events are inferred. The Aptian Radiation involves the initial diversification of Notosuchia and originated lineages recorded in South America, Africa, and China. The Turonian-Santonian Radiation involves the diversification of three clades (advanced notosuchians, Sebecosuchia+*Comahuesuchus*, and South American peirosaurids), which are exclusively recorded in the Late Cretaceous of South America.

We discuss in this section the implications from an evolutionary and biogeographical point of view two major episodes in the evolution of Notosuchia, an initial radiation of basal lineages that must have occurred by the Aptian (Early Cretaceous) and with more detail a subsequent radiation of South American notosuchians in the Late Cretaceous (Figure 47). Additionally, we discuss some common aspects of functional inferences of lower jaw motion that can be inferred from the highly modified mandibular and dental morphology of advanced notosuchians.

#### The Early Cretaceous radiation of Notosuchia

The increased taxonomic content of Notosuchia retrieved in recent phylogenetic analyses (e.g., [18], [20], [26]), as well as in this study, implies the existence of three major notosuchian clades: uruguaysuchids, peirosaurids (usually allied to mahajangasuchids), and ziphosuchians. In our analysis (Figure 31), Ziphosuchia includes an assemblage of basal forms (e.g., *Candidodon*, *Libycosuchus*, *Simosuchus*, *Pakasuchus*+*Malawisuchus*), advanced notosuchians, and sebecosuchians. The three major clades of Notosuchia mentioned above have their oldest records in the Early Cretaceous. Early records of Uruguaysuchidae include Aptian-Albian forms from Africa (*A. wegeneri*, *Anatosuchus* [24]) and South America (*A. gomesii*). The clade of mahajangasuchids+peirosaurids includes in our analysis the Albian-Cenomanian *Hamadasuchus* from Morocco and the Aptian-Albian *Stolokrosuchus* from Niger. The earliest records of Ziphosuchia are known from the Early Cretaceous of different land-masses. These include the Aptian *Candidodon* from South America, the Aptian-Albian *Malawisuchus*+*Pakasuchus* clade from Africa, and the conflictive Aptian-Albian *Chimaerasuchus* from China.

A calibrated phylogeny, integrating the phylogenetic results with the temporal occurrence of taxa, reveals the existence of a large initial radiation of Notosuchia that must have occurred by the Aptian (Figure 47). The phylogenetic position of all Early Cretaceous notosuchians are indicative of the existence of multiple ghost lineages that date back to this time, several of which lead to notosuchians that are only known from Late Cretaceous rocks (e.g., mahajangasuchids, South American peirosaurids, *Libycosuchus*, *Simosuchus*, advanced notosuchians, sebecosuchians; Figure 47). Despite some basal nodes are not strongly supported, the existence of an initial radiation of notosuchians during the Aptian is a relatively robust inference, and in fact multiple ghost notosuchian lineages extending into the Early Cretaceous are implied by most notosuchian phylogenies published to date (see [18]).

The initial radiation of Notosuchia in the Early Cretaceous involved not only the origin of major taxonomic clades but also the appearance of distinct morphological features that are indicative of ecological diversity. These include the small-bodied uruguaysuchids, the generalized morphology of peirosaurids, the longirrostrine *Stolokrosuchus*, and the appearance of heterodonty and a remarkable diversity of multicusped teeth in basal ziphosuchians (e.g., *Candidodon*, *Malawisuchus*, *Pakasuchus*, *Chimaerasuchus*).

Notosuchians have been regarded as a typical gondwanan clade [2], [81], forming part of the distinctive vertebrate faunal assemblage recorded in the Cretaceous of Gondwana [82]. The biogeographic pattern of the distribution of notosuchians within Gondwana, however, has been debated in recent years. Alternative hypotheses have been proposed, arguing for vicariant events during the Cretaceous break up of Gondwana [83] or climatic controls [84] as the major driving forces for explaining the geographic distribution of notosuchian crocodyliforms within the Cretaceous of Gondwana.

The spatial distribution of the lineages involved in the initial diversification of Notosuchia (South America, Africa, Madagascar) is compatible with a widespread geographic setting of this radiation across Gondwana [18], but includes the still enigmatic presence of *Chimaerasuchus* in the Early Cretaceous of China (Figure 47). The notosuchian affinities of *Chimaerasuchus* are relatively well supported in our analysis (see below) and could be explained either by a widespread distribution of basal notosuchian lineages or a case of long-range dispersal, as noted by Turner & Sertich [18]. An important aspect related to the complex biogeographic history of basal notosuchians is that the origin of this lineage undoubtly extends back to the Early Jurassic [1], [18], [85]. This indicates that we currently have record of only the last half of the 100 million years of notosuchian evolution during the Mesozoic (Figure 47). The lack of knowledge on the first half of notosuchian evolutionary history clearly hampers our understanding on the early biogeographic history of this group [18]. Future discoveries in the earliest Cretaceous and Jurassic will likely provide key data for understanding the origins of Notosuchia, including a more stringent test of the existence of the Aptian radiation event.

#### The Late Cretaceous radiation of advanced notosuchians and sphagesaurids

The clade of advanced notosuchians includes in our analysis at least twelve taxa, eight of which are clustered in Sphagesauridae. The first advanced notosuchian to be described was *Notosuchus terrestris* [86]. Over half a century passed until Price [8] described the second member of this group (i.e. type material of *Sphagesaurus huenei*) and more detailed studies on *Notosuchus* were published [2], [78]. Subsequently, the third member of this clade, *Mariliasichus amarali*, was published by Carvalho & Bertini [87]. However, it was only during the last ten years that the remaining ten species of advanced notosuchians were described. This recent increase in diversity was driven by the remarkable discoveries of crocodyliforms in the Late Cretaceous Bauru Group of Brazil [7], [10]–[14], [74], [76], [77] as well as that of the well-preserved remains of *Yacarerani* from Bolivia [15]. These discoveries also prompted additional studies on the anatomy of advanced notosuchians based on more complete specimens of some taxa [5], [6], [17], [46], [61], [64], [68], [75], [84], [88]–[91]. This large number of recent publications on advanced notosuchians has provided a wealth of new anatomical information during the last decade that has not been yet fully understood and synthetized. The disparate results on their relationships obtained in previous phylogenetic studies exemplify the lack of a thorough understanding on the evolution of this group.

It is within this context that our phylogenetic analysis, based on an extended taxon and character sampling, provides important results for understanding the evolutionary history of advanced notosuchians. Although there are multiple topological uncertainties in our results (see Phylogenetic Analysis), the monophyly of the clade of advanced notosuchians and of Sphagesauridae is robustly supported by the addition of new character data. The monophyly of these two groups is the major difference with respect to previous studies, except for that of Andrade *et al.* [20] that also recovered these two clades as monophyletic (although with some differences in their internal topology). The monophyly and high support of these groups is relevant given they cluster forms exclusively found in the Late Cretaceous of South America (see below) and include some outstandingly abundant species (e.g. *Notosuchus*, *Mariliasuchus*). Sphagesauridae is an important component of the radiation of advanced notosuchians, as it includes more than half of their diversity and has the broadest range of body size and the largest morphological disparity in their dentition (see below).

The monophyly of advanced notosuchians and the support values obtained in our study provides a robust scenario for an evolutionary radiation of advanced notosuchians that occurred in South America during the Late Cretaceous. The calibration of the phylogenetic hypotheses against geologic time illustrates the Late Cretaceous timing of this radiation (Figure 47), which likely occurred by the Santonian but may be as old as the Turonian. The uncertainty on the precise timing of this radiation stems from the uncertainties on the age of some key lithological units from South America. Four different lithological units have provided each a single taxon of advanced notosuchians: Bajo de la Carpa Formation (southern Argentina), Cajones Formation (central Bolivia), Marilia Formation (southestern Brazil), and Alcântara Formation (northeastern Brazil). More than half of the diversity of advanced notosuchians, however, comes from the Adamantina Formation (nine taxa; southern Brazil). Although these units have been regarded as Late Cretaceous by most recent authors, there is some degree of uncertainty on their age at the stage level and there are no radioisotopic dates for any of them.

The Marilia Formation (Bauru Group) has only provided so far the remains of the fragmentary *Labidiosuchus amicum* [74]. Its age is commonly regarded as Campanian-Maastrichtian based on faunal correlations and this unit overlies the Adamantina Formation. Therefore, its age is only important to determine the length of the ghost lineage leading to *Labidiosuchus* but not to determine the age of the radiation of advanced notosuchians (Figure 47).

The Cajones Formation yielded the type material of *Yacarerani*, including several specimens found in close association with eggs [15]. This unit was originally interpreted as Maastrichtian based on fish remains [92], [93] but Novas *et al.* [15] considered most likely a Turonian-Santonian age due to the close affinities of *Yacarerani* and *Adamantinasuchus*. Unfortunately, no other geochronological information is available for this unit. Based on the available evidence, precising the age of this unit would only help determining the length of the ghost lineage leading to *Yacarerani* but not the timing of the advanced notosuchian radiation (Figure 47).

The Bajo de la Carpa Formation (Neuquén Group) crops out in northwestern Patagonia (Argentina) and yielded abundant remains of *Notosuchus*, as well as *Comahuesuchus*, baurusuchids, and peirosaurids (see below). This unit was regarded as Santonian based on its vertebrate fauna [78], [94]–[97]. Although no radioisotopic dates are available for the Bajo de la Carpa Formation, this unit is overlied by the Anacleto Formation [95], for which paleomagnetic data has yielded a lower Campanian age [98]. Therefore, a Santonian age for this unit has been generally accepted. The age of the Bajo de la Carpa Formation indicates the radiation of advanced notosuchians must have started at least by the Santonian.

The vast majority of advanced notosuchians comes from the Adamantina Formation (Bauru Group) that crops out in São Paulo and Minas Gerais states, Brazil. There are two main interpretations for the age of this unit based on the biostratigraphic information of ostracods and vertebrates and the disputed existence of a Campanian depositional hiatus in the Bauru Group. The two proposed ages for the Adamantina Formation are Turonian-Santonian [41], [99] or Campanian-Maastrichtian [37], [100]–[103]. These alternative interpretations have been reflected in the notosuchian literature, with some authors referring the crocodyliforms from the Adamantina Formation as Turonian-Santonian [7], [11], [14], [77], [104]–[107] or Campanian-Maastrichtian [10], [12], [17], [29], [108]. Given the large diversity of advanced notosuchians in the Adamantina Formation, defining more precisely its geological age is critical to understand more precisely the timing of the radiation of advanced notosuchians (as well as that of other notosuchian clades such as baurusuchids and peirosaurids). If this unit were indeed deposited during the Turonian-Santonian, the radiation of advanced notosuchians (and baurusuchids) would be narrowly constrained to this interval. The alternative interpretation would suggest the radiation event was slightly younger and extended into the Campanian, but certainly starting in the Santonian given the age of the Bajo de la Carpa Formation.

The Alcântara Formation (Itapecuru Group) crops out in northeastern of Brazil and has yielded the fragmentary *Coringasuchus anisodontis* [76]. The age of this unit is regarded as early Cenomanian based on its vertebrate fossil content [76]. Therefore, the poorly known *Coringasuchus anisodontis* may represent the oldest advanced notosuchian known to date. Unfortunately, the fragmentary nature of the type specimen (a partial dentary) does not allow determining a precise phylogenetic position for this taxon within the clade of advanced notosuchians (Figure 32), and this taxon can be positioned almost anywhere within Notosuchia with one or two extra steps. More remains are needed to understand its putative affinities with this clade and its impact on the timing of the radiation of advanced notosuchians.

Despite the uncertainties noted above on the geochronological constraint of these units, the available evidence suggests that the radiation of advanced notosuchians was already ongoing by the Santonian, and may have started in the Turonian (Figure 47) depending on the age of the Adamantina Formation. Interestingly, a similar temporal pattern of diversification is inferred for two other groups of notosuchians from the Late Cretaceous of South America: *Comahuesuchus*+Sebecosuchia and South American peirosaurids (Figure 47).

The diversification of the *Comahuesuchus*+sebecosuchians clade must have started at least in the Santonian (Figure 47), given the age of the Bajo de la Carpa Formation that yielded *Comahuesuchus* and the basal baurusuchid *Cynodontosuchus*. The largest diversity of baurusuchids is known from the Adamantina Formation, so that the radiation of baurusuchids mirrors the case of the diversification of advanced notosuchians, which was already initiated by the Santonian but could be as old as Turonian (depending on the age of the Adamantina Formation).

The clade formed by South American peirosaurids is also inferred to diversify in the same period of time. The oldest well-preserved member of this clade is the Turonian-Coniacian *Lomasuchus* from the Portezuelo Formation of Argentina and its currently known diversity also includes the Santonian *Gasparinisuchus* from the Bajo de la Carpa Formation, two latest Cretaceous taxa from the Marilia Formation (*Uberabasuchus*, *Peirosaurus*), as well as *Montealtosuchus* from the Adamantina Formation (Turonian-Santonian or Campanian-Maastrichtian). Putative peirosaurid material have also been reported from late Early Cretaceous or early Late Cretaceous beds of Patagonia [109], [110] but their fragmentary nature precludes determining if they belong to the restricted clade formed by the above mentioned South American taxa or the more inclusive clade in which the Albian-Cenomanian *Hamadasuchus* from Africa is included.

Thus, the Turonian-Santonian is inferred to be a critical time not only for advanced notosuchians but also for two other important groups of notosuchian crocodyliforms from South America (Figure 47). As noted by Turner & Sertich [18], the South American record shows a marked increase in the alpha taxonomic diversity of notosuchians during the Turonian-Santonian. Our phylogenetic results show that this increase in diversity mainly occurred in the three most successful clades of South American notosuchians, which seem to have undergone a major diversification phase during this time.

#### The South American endemism of advanced notosuchians and sphagesaurids

Despite the complexities and uncertainties on the early biogeographic history of Notosuchia noted above, a clear (and well supported) biogeographic pattern arises from our study, the South American endemism of advanced notosuchians. This contrasts with inferences made from most previous phylogenetic studies in which advanced notosuchians (and sphagesaurids in particular) have been proposed as closely related to two non-South American notosuchians. First, *Chimaerasuchus paradoxus* from China was depicted as a close relative of either *Notosuchus* [67] or *Sphagesaurus huenei* [5], [15], [17]–[18], [56], [58]. Second, *Pakasuchus kapilimai* was proposed as a close relative of *Adamatinasuchus* and *Mariliasuchus* [19], a result that was interpreted as evidence that these lineages of notosuchians evolved in South America and Africa while these land-masses were still connected to each other.

In contrast to these interpretations, our phylogenetic results (as well those in [20]) support the monophyly of advanced notosuchians (including Sphagesauridae), clustering only crocodyliforms that are known from the Late Cretaceous of South America (Figure 47). The geographic distribution of this clade is limited to the central half of South America, including the early appearing but poorly known *Coringasuchus* (see [84]: Figures 1–2). As a major component of advanced notosuchians, Sphagesauridae plays a pivotal role in understanding the taxonomic, morphological, and ecological diversity of this endemic clade. Although some authors regarded sphagesaurids as restricted to the highly fossiliferous Bauru Group of Brazil [84], the inclusion of *Yacarerani* in this clade in our study (as well as in [20]) shows that this group inhabited other areas of South America.

A noteworthy aspect of our results is the existence of strong character support for the endemic clade of advanced notosuchians and sphagesaurids (especially if we ignore the putative alternative positions of the highly fragmentary *Coringasuchus*). These two groups have high support values (e.g., Bremer = 4; bootstrap = 71–75%; jackknife = 79–81%) and provide a robust phylogenetic framework for inferring the endemic nature of this group. Most importantly, placing any of the non-South American notosuchians within this clade leads to markedly suboptimal hypotheses. In particular, forcing *Chimaerasuchus* or *Pakasuchus* within this group results in trees that are 5 and 9 steps longer than the MPTs, respectively. These tests demonstrate that the inclusion of Asian or African forms as part of advanced notosuchians (or sphagesaurids) is clearly rejected within the context of our phylogenetic analysis. A similar biogeographic pattern is present for the two clades that also seem to have radiated at the same time in South America, peirosaurids and baurusuchids (Figure 47; see Document S1).

One of the outstanding aspects of the endemic clade of advanced notosuchians is their numerical abundance and diversity in the deposits where they are found. In both the Adamantina and Bajo de la Carpa formations these crocodyliforms clearly outnumber all other groups of vertebrates (including dinosaurs) [1], [84], and some advanced notosuchians (e.g., *Mariliasuchus*, *Notosuchus*) are among the most abundant taxa known from these units. The causes underlying the abundance and diversity of notosuchians in these particular deposits and their rarity or absence in other deposits from the Late Cretaceous of South America is intriguing and have not been fully explored. Carvalho *et al.* [84] provided a general explanation for the distribution and diversity of Notosuchia, relating their presence to the extension of environments characterized by marked seasonality and aridity during the warm Cretaceous climate. Whether or not this explanation is adequate for the entire clade Notosuchia, it seems to apply perfectly to the distribution of advanced notosuchians, all of which are found in red beds of fluvial and/or eolian origins deposited in warm and semiarid environments [37], [84], [95]. These similarities indicate these taxa seem to have thrived in this type of environment, although we currently do not know if these deposits (i.e., Adamantina, Bajo de la Carpa, and Cajones formations) were formed at the same time or not.

#### Jaw motion in advanced notosuchians and sphagesaurids

The members of the radiation of advanced notosuchians have a considerable diversity in terms of their body size, robustness, rostral anatomy, dentition, and development of body armor. They include small bodied and gracile forms, characterized by a relatively short rostrum and reduced body armor (e.g., *Mariliasuchus*, *Adamantinasuchus*, *Yacarerani*) as well as large and robust forms with a relatively elongated snout and extensive body armor (e.g., *Armadillosuchus*). Many of these forms are found in the same deposits (e.g., Adamantina Formation) and given their disparity in body size and rostral and dental anatomy, they provide an example of ecological diversity and niche partitioning within this clade, suggesting advanced notosuchians underwent an adaptive radiation during the Late Cretaceous of South America.

With the exception of the basal and poorly known *Morrinhosuchus*, all these forms share a set of common derived features in their dentition and lower jaw that indicates the existence of a similar jaw occlusion motion (highly modified from the generalized orthal jaw motion of most crocodyliforms). These features include the narrow and elongated mandibular symphysis (char. 363.1; Figure 40), the distinctive morphology of the retroarticular process (char. 378.1, 379.1; Figure 46), the oblique orientation of posterior tear-drop shaped teeth (char. 137.1; Figures 40, 44), and the placement and orientation of the wear facets on the molariform crowns (char. 395.1; Figure 33). In particular, the wear facets are informative for inferring jaw motion during occlusion. These are indicative of tooth-tooth occlusion [3], [5] and are remarkably extensive and obliquely oriented (Figures 33E–G, 40E–H, 44D), suggesting the presence of fore-aft jaw motion that included a marked lateral component during occlusion, as well as the presence of alternated unilateral occlusion pattern. These inferences were originally made for *Sphagesaurus huenei* [5], but new discoveries show that this pattern is actually present in most advanced notosuchians [3], [7], [13], [15], [61], [64].

The alternated unilateral occlusion pattern inferred for these forms is a unique and highly modified functional aspect of this group in comparison with all other crocodyliforms. The inferred jaw motion of advanced notosuchians implies major changes in the jaw mechanics and architecture of the adductor musculature with respect to the plesiomorphic condition of crocodyliforms [3]. Moreover, this derived jaw motion pattern is conserved in taxa that differ in many aspects of their anatomy (see above), indicating the alternated unilateral occlusion pattern likely was flexible enough to be apt for different feeding habits [3].

As mentioned above, the highly modified feeding apparatus of advanced notosuchians includes a major fore-aft motion of the lower jaw during the unilateral occlusion of the upper and lower toothrows. The presence of fore-aft motion in the lower jaw, however, is not unique of advanced notosuchians. Closely related outgroups of this clade, such as *Chimaerasuchus*, the *Pakasuchus*+*Malawisuchus* clade, and to some extent *Simosuchus*, have dental and mandibular features that indicate the existence of fore-aft jaw motion [3], [4], [19], [67], [79], although there is no indication of a marked lateral component or unilateral occlusion in these taxa [3]. Therefore, the evolution of fore-aft jaw motion was an early modification during the evolution of Notosuchia, which likely appeared jointly with the multicusped dentition during the initial radiation of Ziphosuchia in the Aptian (Early Cretaceous; Figure 47).

Within this context, the presence of ziphodont dentition and carnivorous habits of baurusuchids (and sebecids) is optimized as a derived modification convergently acquired with other crocodyliforms (e.g. peirosaurids, *Hsisosuchus*). Alternative hypotheses clustering in a monophyletic group all heterodont notosuchians with inferred fore-aft movement of the lower jaw (i.e. excluding baurusuchids and sebecids) are markedly suboptimal and imply a minimum of eight extra steps.

### Outstanding problems and uncertain affinities of particular taxa

Our phylogenetic analysis includes some robustly supported nodes, such as those of advanced notosuchians and sphagesaurids discussed in the phylogenetic sections and the first section of the discussion. However, our analysis also includes uncertainties regarding the phylogenetic position of some taxa. We already mentioned the labile position of some extremely fragmentary taxa, such as *Coringasuchus*, *Pehuenchesuchus*, *Pabwehshi*, *Labidiosuchus*, and *Morrinhosuchus*. We discuss here the low support and the relevant evidence of two other taxa, *Chimaerasuchus paradoxus* and *Comahuesuchus brachybuccalis*. These have been retrieved in different positions in recent analyses and are of particular interest because of different reasons, so their alternative positions in suboptimal trees are discussed below.

#### The affinities of *Chimaerasuchus*

This taxon was originally described as the first Laurasian notosuchian [111] and still represents the only record of this clade outside Gondwana during the Cretaceous, so its position is relevant from a biogeographical point of view. *Chimaerasuchus* has been consistently retrieved within Notosuchia in all phylogenetic analyses that included this taxon [5], [10], [11], [15]–[18], [21], [26], [56], [58], [67], [112], with the sole exception of one study that placed basally within Crocodyliformes [113]. Its phylogenetic position, however, has varied in the different analyses published to date. *Chimaerasuchus* is a challenging taxon to place phylogenetically due to its highly autapomorphic anatomy and its relatively fragmentary type material, consisting of a rostrum, a badly preserved lower jaw, and isolated postcranial elements [67]. The absence of critical anatomical regions in *Chimaerasuchus*, such as the palate and the skull roof hampers our understanding of its affinities as they usually provide informative phylogenetic variation within (and outside) Notosuchia.

Most previous studies (with the exception of [10]) placed *Chimaerasuchus* as closely related to advanced notosuchians, either with *Notosuchus* or with some sphagesaurid taxa. Within the context of our analysis the inclusion of *Chimaerasuchus* within advanced notosuchians is markedly suboptimal and implies a minimum of five extra steps and reaching eleven extra steps if it is forced within Sphagesauridae. Characters that cause this increase in tree length include features of the mandibular and dental morphology. For instance the mandibular symphysis of advanced notosuchians differ from that of *Chimaerasuchus* in that the former have an anteriorly tapering smypyhsis (char. 154.0; Figure 40), with a distinct anterior region with parallel lateral margins (char. 363.1; Figure 40). Additionally, the posterior region of the dentaries of advanced notosuchians has a broad shelf so that the posterior teeth are markedly inset medially from the lateral surface of the dentaries (char. 193.1; Figure 36B). An important difference in the mandible is also present in the articular. Although *Chimaerasuchus* has been originally interpreted as having an elongated articular glenoid facet for the quadrate condyles [111], the holotype lacks a markedly elongated articular that characterizes advanced notosuchians (char. 104.1; Figure 46G–I). Several differences in the dentition of *Chimaerasuchus* and advanced notosuchians also implies extra step is the former is positioned within the latter. *Chimaerasuchus* lacks a transitional tooth located at the premaxilla-maxilla suture (char. 381.0; Figure 44H), the maxillary teeth are set in alveoli (fig. 164.0; Figure 44H) rather than in an alveolar groove, and its posterior teeth lack an obliquely oriented distal carina, apicobasal ridges, and its enamel surface is smooth (chars. 137.0, 386.0, 392.0; Figure 33). A final difference with functional implications is that the tooth-tooth wear facets of posterior teeth in *Chimaerasuchus* are subhorizontal and oriented parallel to the longitudinal plane of the skull (char. 395.0; Figure 33L), rather than being oriented along a plane that is oblique to the longitudinal and sagittal planes of the skull as in advanced notosuchians (Figure 33E–G). Based on these morphological differences and the marked increase in tree length noted above, the exclusion of *Chimaerasuchus* from advanced notosuchians (and in particular its exclusion from Sphagesauridae as originally proposed by Marinho & Carvalho [6], [7]) is strongly supported in our analysis.

**Figure 48:**
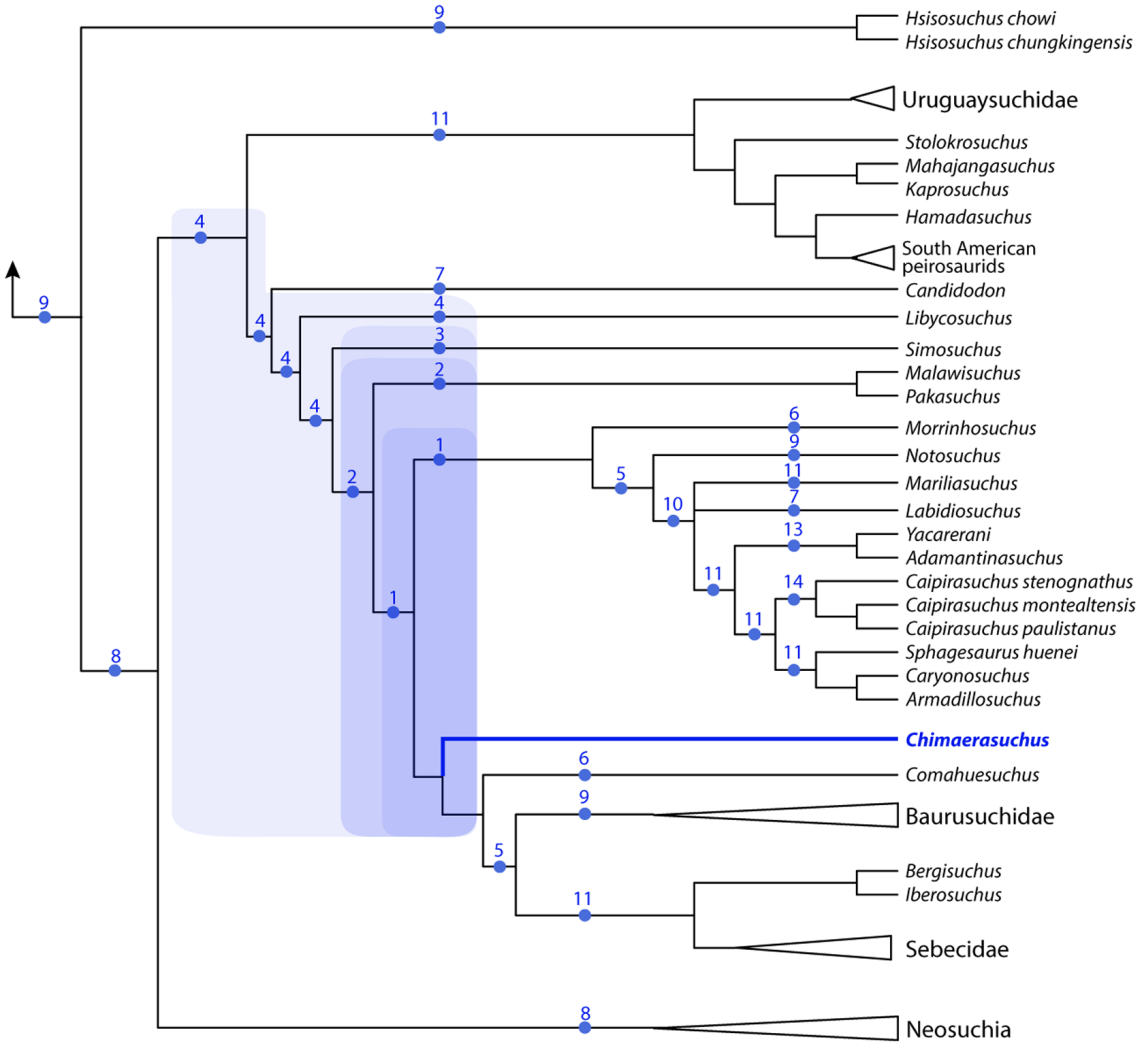
Alternative phylogenetic placements of *Chimaerasuchus paradoxus*. The relationships shown are based on the reduced strict consensus of Notosuchia and most immediate outgroups. Black circles represent the alternative positions of *Chimaerasuchus* and the values above each circle indicate the number of extra steps implied when this taxon is forced in each postion. Shaded area indicates the optimal or marginally suboptimal (up to two extra steps) positions of *Chimaerasuchus*.

The position retrieved in our analysis for *Chimaerasuchus* (as the sister group of *Comahuesuchus* and sebecosuchians; Figures 31, 47) is nonetheless poorly supported in our phylogenetic analysis. Conducting multiple constrained analyses (forcing alternative positions of *Chimaerasuchus* prior to conducting a heuristic tree search) reveals several plausible alternative positions for *Chimaerasuchus*, as well as regions of the tree in which this taxon can only be placed with a marked increase in tree length (Figure 48). From these analyses it becomes clear that, within the context of our dataset, *Chimaerasuchus* can be positioned with one or two extra steps as closely related to (but not within) advanced notosuchians or in a slightly more basal position (close to the *Pakasuchus*+*Malawisuchus* clade or immediately basal to it).

However, forcing this taxon as a basal member of Notosuchia implies four extra steps (Figure 48). The characters that increase in number of steps when a basal position of *Chimaerasuchus* is constrained reflect its derived similarities with derived ziphosuchians. This include the pattern or ornamentation on cranial elements composed by grooves and ridges (char. 0.1; Figure 43F), the absence of an ossified narial septum (char. 66.1), the palatal shelves of premaxilla that do not meet posterior to incisive foramen (char. 7.1; Figure 44H), the presence of unsculpted region along alveolar margin on the lateral surface of maxilla (char. 107.1; Figure 34H–I), the absence of enlarged maxillary teeth (char. 79.0), and presence of tubercular rounded denticles in posterior teeth (char. 120.2; Figure 33K).

Finally, and most importantly, when *Chimaerasuchus* is forced anywhere outside Notosuchia implies at least eight extra steps. The characters involved in this increase in tree length are mostly focused on its rostral anatomy. *Chimaerasuchus* shares with notosuchians a similar pattern of ornamentation of skull bones (char. 1.1; Figure 34H–I), the presence of an incisive foramen at the premaxilla-maxilla contact (char. 270.0; Figure 44H), confluent external nares with a large perinarial fossa (char. 226.1/2), a small neurovascular foramen at the premaxilla-maxilla suture on the lateral surface of the snout (char. 135.1; Figure 34H), an unscuplted surface of the maxilla above the alveolar margin bearing large aligned neurovascular foramina (char. 107.1; char. 138.1; Figure 34H–I), the presence of a well-developed anterior jugal shelf (char. 121.1; Figure 34I), and enlarged premaxillary teeth (char. 78.1; Figure 44H). Furthermore, some postcranial characters also support its inclusion within Notosuchia, such as the presence of rod-like anterior cervical neural spines, dorsally projected cervical prezygapophyses, dorsal vertebrae with dorsoventrally leveled transverse process and postzygapophyses, and humerus with a vertically oriented insertion area of m. subscapularis and a deep circular depression on its posterior surface for the insertion of m. scapulohumeralis caudalis. Although some of these characters are found in other crocodyliforms, the simultaneous presence of these characters is exclusively shared by *Chimaerasuchus* and notosuchians. The partially known anatomy of *Chimaerasuchus*, therefore, is most parsimoniously interpreted as indicative of notosuchian affinities. However, as noted above, more complete remains of this taxon (especially including the palate and skull roof) are necessary to test its position.

This indicates that the notosuchian affinities of *Chimaerasuchus* are strongly supported by our phylogenetic dataset and that this taxon is most parsimoniously depicted in the neighboring nodes (shaded branches in Figure 48) of advanced notosuchians and other strongly heterodont notosuchians with multicusped posterior teeth (e.g., *Pakasuchus*, *Malawisuchus*) that must have appeared during the initial radiation of Notosuchia inferred to occur during the Aptian (Figure 47). Although this is not surprising given the multicusped dental anatomy of *Chimaerasuchus*, as discussed above, character support for its position is not only found in the presence of multicusped teeth.

#### The affinities of *Comahuesuchus*

This taxon was originally described as a member of Notosuchia and a monotypic family was erected for it [78], given the presence of a bizarre, short and broad, rostral anatomy. Subsequent discoveries of an adult specimen provided new anatomical information of this form, including its derived palatal anatomy [55]. Phylogenetic studies that included *Comahuesuchus* resulted in disparate positions for this taxon, as closely related to *Sphagesaurus*+sebecosuchians [5], [21], [56], [58], *Anatosuchus* [47], [114], *Mariliasuchus* [17], [24], to some sphagesaurids [18], or as closely related to some advanced notosuchians, namely some sphagesaurids and *Mariliasuchus* and/or *Notosuchus* [10], [11], [12], [16], [20], [55].

Except for the proposed affinities of *Anatosuchus*, all other hypotheses related *Comahuesuchus* to some advanced notosuchians (and baurusuchids in some cases). The palatal anatomy of *Comahuesuchus* [55] shares derived features with advanced notosuchians and baurusuchids, such as the presence of rod-like palatine bars. The inclusion of *Comahuesuchus* within the clade formed by advanced notosuchians and sebecosuchia (or baurusuchids if Sebecidae is placed in its alternative position [23]) is robustly supported in our analysis. Trees that place *Comahuesuchus* outside this clade are markedly suboptimal, implying a minimum of six extra steps (and 13 extra steps if it is placed more basally than *Pakasuchus*+*Malawisuchus*).

Another alternative position of *Comahuesuchus* that is rejected in our analysis is a deeply nested placement within advanced notosuchians, such as its sister group relationship with *Mariliasuchus* [17] or with some sphagesaurids [18]. These topologies imply a minimum of ten extra steps and are therefore strongly contradicted by the analyzed character data.

However, there is some degree of uncertainty in our analysis regarding two plausible placements of *Comahuesuchus*, either as a basal form of the lineage leading to baurusuchids (and sebecids) or as the sister group of advanced notosuchians. The former position is that of the optimal topologies (Figures 31, 47) whereas the latter resembles the position retrieved for this taxon by Andrade and collaborators [10], [20]. Within the context of our dataset, this alternative placement implies only two extra steps and is found in 30–33% of the trees derived from the bootstrap and jackknife replicates.

The optimal placement of *Comahuesuchus* is supported by some characters of the dentition and lower jaw, such as the presence of an enlarged maxillary tooth (char. 79.1; Figure 44G), maxillary dentition set in discrete alveoli (char. 164.0; Figure 44G), the presence of a dorsal projection of the alveolar margin at the level of a large caniniform (char. 159.2; Figure 36D), whose mesial margin has denticles similar as those of ziphodont forms (char. 120.0; MUC-PV 202; Figure 33M), an anteriorly convex rostral margin of the dentaries in ventral view (char. 154.1), and posterior peg of splenials dorsally displaced from the ventral surface of the mandibular symphysis (char. 370.1; Figure 45H). Additionally, there is a large foramen on the palate at the premaxilla-maxilla surface (char. 284.1; Figure 44G) and the antorbital fenestra is completely obliterated (char. 67.2).

The putative affinities of *Comahuesuchus* with advanced notosuchians, instead, are supported by characters of the rostrum and mandible, including the presence of a laterally concave nasal-premaxilla suture (char. 127.0; Figure 43D), a flat surface of the anterior region of the jugal bearing a large jugal foramen (char. 230.1), a short and broad prefrontal oriented posteromedially-anterolaterally (char. 111.1), a transversely oriented splenial-dentary suture on the ventral surface of the mandibular symphysis (char. 185.1), and the anterior opening of the mandibular nerve located on the anteroposterior midpoint of the splenial (char. 368.2; Figure 45H).

In summary, there is uncertainty on the position of the bizarre *Comahuesuchus* in our analysis but, contrasting with the disparate results of previous studies, the two most plausible placements of this taxon are restricted to a basal position within two closely related lineages of notosuchians that radiated in the Late Cretaceous of South America: sebecosuchians and advanced notosuchians. Further remains of this incompletely known taxon would certainly benefit our understanding on its debated affinities.

## Conclusions

The discovery of *Caipirasuchus stenognathus* and the phylogenetic analysis presented here provide important insights for understanding the diversity and evolution of notosuchians in the Cretaceous of Gondwana. The new taxon shares numerous derived features present in *Caipirasuchus paulistanus*, a recently described taxon that also comes from the Adamantina Formation of Brazil [11]. These two taxa are also remarkably similar to “*Sphagesaurus*” *montealtensis* [10] and the phylogenetic analyses cluster the three taxa in a monophyletic group, providing support for the referral of these species to the genus *Caipirasuchus* [12]. The new specimen described here has an exquisitely preserved dentition and skull that provides new and relevant information to understand the morphological diversity of sphagesaurids; a diverse but still poorly understood clade of notosuchian crocodyliforms.

Our phylogenetic analysis is based on extended character and taxon sampling, adding a significant amount of new phylogenetic data on the evolution of notosuchians (with particular emphasis on the craniomandibular and dental anatomy). The calibration of the phylogenetic hypotheses against geological time reveals two major episodes in the evolutionary history of Notosuchia: an initial radiation inferred to occur at least by the Aptian and a subsequent stage in the Turonian-Santonian during which radiated three major clades of South American notosuchians.

An important result of our phylogenetic analysis is the topology of derived notosuchians, retrieving a monophyletic group of advanced notosuchians that includes well-known taxa such as *Notosuchus*, *Mariliasuchus*, and a monophyletic Sphagesauridae (which includes eight taxa). The monophyly of these two groups contrasts with most previous phylogenetic studies but agrees with a recent study [20] that also retrieved these two groups as monophyletic, although with different internal resolutions. Our phylogenetic analysis identifies strong character support for the monophyly of both advanced notosuchians and Sphagesauridae. The latter clade has outstanding similarities in the dentition and lower jaw that provide a clear phylogenetic signal supporting their monophyly.

The recognition of the monophyly of advanced notosuchians and Sphagesauridae has multiple implications for understanding the evolution of notosuchian crocodyliforms. First, the temporal and spatial distribution of this group leads to the recognition of a remarkable radiation of advanced notosuchians that are exclusively found in the Late Cretaceous of South America, rejecting previous hypotheses that allied some advanced notosuchians or sphagesaurids with notosuchians recovered in other land masses (e.g., Africa, Asia).

Second, despite the restricted temporal and geographic distribution of the endemic clade of advanced notosuchians, its diversity is remarkably high in comparison with that of other clades of notosuchian crocodyliforms. This clade is taxonomically diverse (including up to 13 taxa) and includes considerable variation in terms of body size, rostral anatomy, and dental morphology, suggesting advanced notosuchians underwent an adaptive radiation during the Late Cretaceous in South America.

Third, all the members of this clade known from well-preserved remains have dental and mandibular characters that indicate the existence of a highly apomorphic functional mechanics of the lower jaw. The feeding mechanics inferred for most of these forms include not only the presence of fore-aft movements of the lower jaw, but also an important lateral component during jaw movement and the presence of an alternate unilateral occlusion pattern.

Several critical issues on the phylogeny of derived notosuchians are identified and discussed. These include the recognition of nodes that are well supported and provide a new arrangement of notosuchians, but also include several poorly known taxa with labile phylogenetic position that should be the focus of future research based on more complete specimens (e.g., *Coringasuchus*, *Labidiosuchus*, *Morrinhosuchus*, *Pehuenchesuchus*, *Pabwehshi*). In particular, two important notosuchians of uncertain affinities are *Chimaerasuchus* and *Comahuesuchus*. The former is the only notosuchian taxon known from outside Gondwana and has uncertain affinities within strongly heterodont notosuchians but can be confidently placed outside advanced notosuchians and sphagesaurids (contra most previous studies). Its inclusion in Notosuchia, however, is well supported although more complete remains, including the palate and skull roof will be critical to thoroughly test its affinities. *Comahuesuchus* is a bizarre notosuchian that in our analysis can be either placed as a basal form of the lineage leading to baurusuchids and sebecids or, less parsimoniously, as the sister group of advanced notosuchians. However, its previously proposed affinities with *Anatosuchus*, *Mariliasuchus* and/or sphagesaurids are rejected by our analysis.

## Supporting Information

Dataset S1 Data matrix in nexus format.
(TXT)

Document S1 Phylogenetic Dataset and results.
(PDF)
